# Motixafortide and G-CSF to mobilize hematopoietic stem cells for autologous transplantation in multiple myeloma: a randomized phase 3 trial

**DOI:** 10.1038/s41591-023-02273-z

**Published:** 2023-04-17

**Authors:** Zachary D. Crees, Michael P. Rettig, Reyka G. Jayasinghe, Keith Stockerl-Goldstein, Sarah M. Larson, Illes Arpad, Giulio A. Milone, Massimo Martino, Patrick Stiff, Douglas Sborov, Denise Pereira, Ivana Micallef, Gemma Moreno-Jiménez, Gabor Mikala, Maria Liz Paciello Coronel, Udo Holtick, John Hiemenz, Muzaffar H. Qazilbash, Nancy Hardy, Tahir Latif, Irene García-Cadenas, Abi Vainstein-Haras, Ella Sorani, Irit Gliko-Kabir, Inbal Goldstein, Debby Ickowicz, Liron Shemesh-Darvish, Shaul Kadosh, Feng Gao, Mark A. Schroeder, Ravi Vij, John F. DiPersio

**Affiliations:** 1 Division of Oncology, Washington University School of Medicine in St. Louis, St. Louis, MO USA; 2 Division of Hematology-Oncology, UCLA School of Medicine, Los Angeles, CA USA; 3 Division of Hematology, Department of Internal Medicine, Faculty of Medicine, University of Debrecen, Debrecen, Hungary; 4 Unità di Trapianto Emopoietico, Azienda Ospedaliero Universitaria ‘Policlinico-San Marco’, Catania, Italy; 5 Unit of Stem Cell Transplantation and Cellular Therapies, Grande Ospedale Metropolitano Bianchi-Melacrino-Morelli, Reggio Calabria, Italy; 6 Loyola University Medical Center, Maywood, CA USA; 7 Huntsman Cancer Institute, University of Utah School of Medicine, Salt Lake City, UT USA; 8 Sylvester Comprehensive Cancer Center, University of Miami Health System, Miami, FL USA; 9 Division of Hematology, Mayo Clinic, Rochester, MN USA; 10 Department of Hematology, Ramon y Cajal University Hospital, Madrid, Spain; 11 Center Hospital of Southern Pest, National Institute of Hematology and Infectious Diseases, Budapest, Hungary; 12 Hospital University 12 De Octubre, Madrid, Spain; 13 Department I of Internal Medicine, Medical Faculty and University Hospital of Cologne, University of Cologne, Cologne, Germany; 14 Division of Hematology-Oncology, University of Florida, Gainesville, FL USA; 15 Department of Stem Cell Transplantation and Cellular Therapy, University of Texas MD Anderson Cancer Center, Houston, TX USA; 16 Marlene and Stewart Greenebaum Comprehensive Cancer Center, University of Maryland School of Medicine, Baltimore, MD USA; 17 Division of Hematology-Oncology, University of Cincinnati, Cincinnati, OH USA; 18 Department of Hematology, Hospital de la Santa Creu i Sant Pau, Barcelona, Spain; 19 BioLineRx, Ltd., Modi’in, Israel; 20 StatExcellence, Ltd., Kyriat Mozkin, Israel; 21 Division of Public Health Sciences, Washington University School of Medicine in St. Louis, St. Louis, MO USA

**Keywords:** Stem-cell research, Randomized controlled trials, Myeloma, Haematopoietic stem cells, Multipotent stem cells

## Abstract

Autologous hematopoietic stem cell transplantation (ASCT) improves survival in multiple myeloma (MM). However, many individuals are unable to collect optimal CD34^+^ hematopoietic stem and progenitor cell (HSPC) numbers with granulocyte colony-stimulating factor (G-CSF) mobilization. Motixafortide is a novel cyclic-peptide CXCR4 inhibitor with extended in vivo activity. The GENESIS trial was a prospective, phase 3, double-blind, placebo-controlled, multicenter study with the objective of assessing the superiority of motixafortide + G-CSF over placebo + G-CSF to mobilize HSPCs for ASCT in MM. The primary endpoint was the proportion of patients collecting ≥6 × 10^6^ CD34^+^ cells kg^–1^ within two apheresis procedures; the secondary endpoint was to achieve this goal in one apheresis. A total of 122 adult patients with MM undergoing ASCT were enrolled at 18 sites across five countries and randomized (2:1) to motixafortide + G-CSF or placebo + G-CSF for HSPC mobilization. Motixafortide + G-CSF enabled 92.5% to successfully meet the primary endpoint versus 26.2% with placebo + G-CSF (odds ratio (OR) 53.3, 95% confidence interval (CI) 14.12–201.33, *P* < 0.0001). Motixafortide + G-CSF also enabled 88.8% to meet the secondary endpoint versus 9.5% with placebo + G-CSF (OR 118.0, 95% CI 25.36–549.35, *P* < 0.0001). Motixafortide + G-CSF was safe and well tolerated, with the most common treatment-emergent adverse events observed being transient, grade 1/2 injection site reactions (pain, 50%; erythema, 27.5%; pruritis, 21.3%). In conclusion, motixafortide + G-CSF mobilized significantly greater CD34^+^ HSPC numbers within two apheresis procedures versus placebo + G-CSF while preferentially mobilizing increased numbers of immunophenotypically and transcriptionally primitive HSPCs. Trial Registration: ClinicalTrials.gov, NCT03246529

## Abstract (web summary)

The phase 3 GENESIS trial reports the superiority of the novel CXCR4 inhibitor motixafortide with G-CSF in mobilizing hematopoietic progenitor cells for autologous stem cell transplantation in multiple myeloma.

## Main

Multiple myeloma (MM) is the second most common hematologic malignancy^1^, historically associated with median overall survival (OS) of 24–30 months. However, the development of high-dose chemotherapy and autologous stem cell transplantation (ASCT), immunomodulatory drugs (IMiDs), proteasome inhibitors (PIs), anti-CD38 monoclonal antibodies (mAbs) and other novel therapies has greatly expanded therapeutic options for newly diagnosed MM. Currently, median OS exceeds 45–82 months with ASCT playing a central role in the treatment paradigm for MM^2,3^.

Autologous stem cell transplantation in MM has been shown to improve event-free survival and OS compared with conventional chemotherapy alone in previously untreated standard-risk MM^4,5^. However, the effectiveness of ASCT relies, in part, on the ability to collect sufficient hematopoietic stem and progenitor cells (HSPCs), typically from peripheral blood (PB). A minimum of ≥2 × 10^6^ CD34^+^ cells kg^–1^ are necessary, while infusion of optimal numbers of ≥5–6 × 10^6^ CD34^+^ cells kg^–1^ is associated with improved engraftment, disease-free survival and OS compared with lower transplant doses^6–8^. Clinically, CD34 expression remains the most common immunophenotypic cell surface marker defining human HSPCs. However, multicolor fluorescence-activated cell sorting (FACS) and single-cell RNA sequencing (scRNA-seq) have illustrated the heterogeneous nature of CD34^+^ HSPCs, identifying immunophenotypically and transcriptionally distinct CD34^+^ subsets ranging from primitive hematopoietic stem cells (HSCs) capable of long-term self-renewal and multilineage potential to relatively differentiated, lineage-committed progenitors^9,10^.

Granulocyte colony-stimulating factor (G-CSF) is widely considered the standard agent for PB HSPC mobilization. Nevertheless, despite the use of G-CSF in mobilization of HSPCs to the PB and after multiple days of apheresis, 40–50% of patients with MM remain unable to collect optimal numbers of HSPCs for ASCT^11,12^. The addition of chemotherapy to G-CSF may incrementally increase mobilization, but also prolongs HSPC mobilization with multiple apheresis days and chemotherapy-related toxicities^13,14^. Meanwhile, protracted mobilization substantially increases the financial and logistical burden to both patients and the healthcare system^14,15^.

The interaction between CXCL12 and its receptor, CXCR4, is critically involved in the retention of HSPCs within the bone marrow and blockade of CXCR4 mobilizes HSPCs to PB^16^. Previous studies have shown the low-affinity (inhibitory constant (Ki), 652 nM), short-acting CXCR4i, plerixafor + G-CSF enhanced mobilization of CD34^+^ HSPCs to PB compared with G-CSF^12,17^. However, despite up to eight injections of G-CSF, four injections of plerixafor and four apheresis procedures, 15–35% of patients remained unable to mobilize optimal HSPC numbers^12,18^. Preclinical and clinical data suggest that CXCR4 expression on CD34^+^ HSPC subsets is variable, with relatively lower CXCR4 expression on primitive CD34^+^ HSCs and multipotent progenitors (MPPs) compared with higher expression on certain lineage-committed CD34^+^ progenitors^19^. These studies suggest that optimization of CXCR4 blockade may increase CD34^+^ HSPC mobilization and mobilize differential HSPC subsets^19^.

Motixafortide (BL-8040) is a selective cyclic-peptide inhibitor of CXCR4 with high affinity (Ki, 0.32 nM), long receptor occupancy and extended clinical activity (>48 h)^20–22^. In a phase 1, two-part study (NCT02073019), motixafortide administered to healthy subjects was safe, well tolerated and led to a sustained five- to seven-fold increase in PB CD34^+^ HSPCs, enabling collection of a median of 11.2 × 10^6^ CD34^+^ cells kg^–1^ in one leukapheresis. In another, single-arm, open-label, dose-escalation study (NCT01010880), motixafortide administered before ASCT to patients with MM undergoing standard HSPC mobilization was safe, well tolerated and led to significant increases in PB CD34^+^ HSPCs.

Therefore, this phase 3 study (GENESIS) was designed to compare the safety and efficacy of motixafortide + G-CSF versus placebo + G-CSF in patients with MM undergoing HSPC mobilization before ASCT (NCT03246529). In addition, immunophenotypic and transcriptional profiling was performed via multicolor FACS and scRNA-seq of CD34^+^ HSPCs mobilized on the GENESIS trial, as well as a contemporaneous, prospectively enrolled cohort of demographically similar patients with MM mobilized with plerixafor + G-CSF (protocol no. 201103349) and three cohorts of healthy, allogeneic HSPC donors (allo-donors) undergoing single-agent mobilization with motixafortide, plerixafor or G-CSF alone (NCT02639559, NCT00241358, protocol no. 201106261, respectively).

## Results

### Patient demographics were comparable across study cohorts

**Fig. 1:**
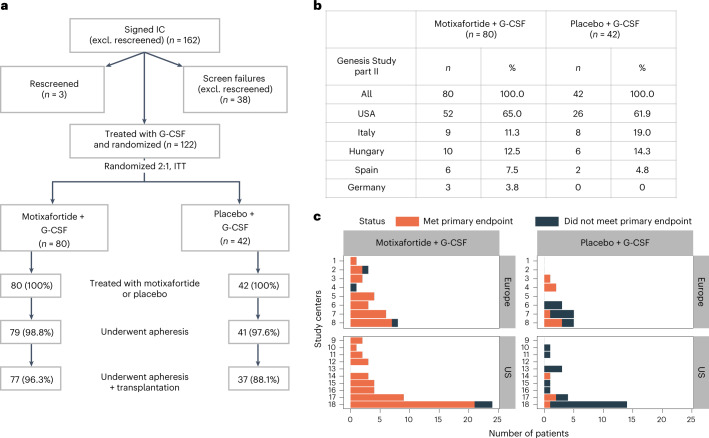
GENESIS trial enrollment. **a**, A total of 162 patients signed informed consent (IC). Screen failures were due to patients not meeting study eligibility criteria; 124 patients began G-CSF mobilization, two elected to withdraw consent before randomization, leaving a total of 122 patients who were randomized (2:1) to either motixafortide + G-CSF or placebo + G-CSF and were included in the ITT analysis. In the motixafortide + G-CSF arm, one patient did not perform apheresis due to an adverse event unrelated to study drug. In the placebo + G-CSF arm, one patient elected not to undergo apheresis due to personal concerns regarding the COVID-19 pandemic. Both these patients were included as mobilization failures in the ITT analysis (that is, did not meet the primary endpoint) but were not remobilized on study. In total, 98.8% (79 of 80) of patients in the motixafortide + G-CSF arm and 97.6% (41 of 42) of patients in the placebo + G-CSF arm received all study-related mobilization injections and underwent apheresis on protocol without any events of treatment arm crossover. **b**, Patients were enrolled across 18 centers and five countries, with the majority treated in the United States. **c**, Enrollment numbers are presented by individual study center, grouped by geographic region (United States and Europe) and mobilization regimen (motixafortide + G-CSF or placebo + G-CSF), with the proportion of patients at each site meeting the primary endpoint (collection of ≥6 × 10^6^ CD34^+^ cells kg^–1^ within two apheresis days) shown in red and the proportion not meeting the primary endpoint shown in black.

**Extended Data Fig. 1:**
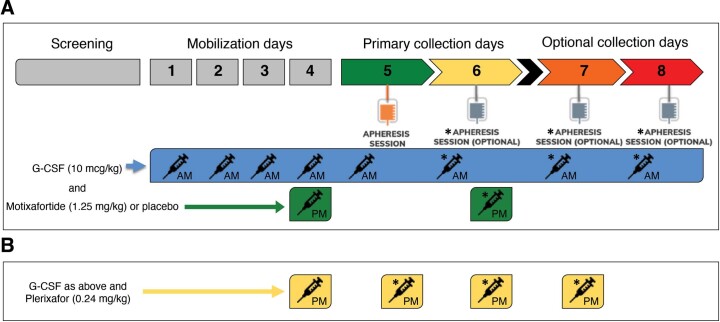
Mobilization and Apheresis Schema. All patients underwent HSPC mobilization and collection on days 1–5 (and days 6–8, if needed). In the AM of days 1–5 (and days 6–8, if needed), all patients received G-CSF (10 mcg/kg) via subcutaneous injection indicated by the syringe in blue. (**a**) GENESIS Trial: In addition to G-CSF, in the PM of day 4 (and day 6, if needed), patients received either motixafortide (1.25 mg/kg) or placebo via subcutaneous injection indicated by the syringe in green. All patients began apheresis (4 blood volumes) on day 5 (and days 6–8, if needed) with the goal of collecting ≥6 × 10^6^ CD34+ cells/kg. Patients collecting to goal completed mobilization. (**b**) Correlative Study: A contemporaneous, cohort of demographically similar MM patients were prospectively enrolled on a parallel protocol and mobilized with G-CSF, as above, plus plerixafor (0.24 mg/kg) via subcutaneous injection on the PM of day 4 (and day 5–7, if needed) indicated by the syringe in yellow. All patients received plerixafor in addition to G-CSF, regardless of PB CD34+ cell count prior to apheresis. All patients began apheresis on day 5 (and days 6–8, if needed) with the goal of collecting sufficient CD34+ cells for ASCT, per institutional practice.

From 22 January 2018 to 30 October 2020, a total of 122 patients from 18 sites in five countries were enrolled and randomized 2:1 to receive either motixafortide + G-CSF (80 patients) or placebo + G-CSF (42 patients) for HSPC mobilization (Fig. 1 and Extended Data Fig. 1a). Demographics between the two treatment arms of the GENESIS trial were similar (Table 1). In addition, the cohort of contemporaneous patients with MM enrolled prospectively and mobilized with plerixafor + G-CSF shared demographics comparable to those of patients on the GENESIS trial (Extended Data Fig. 1b and Extended Data Table 1).

**Table 1:** GENESIS trial demographics

	Motixafortide + G-CSF (*n* = 80)	Placebo + G-CSF (*n* = 42)
Median age (±s.d.), years	63.5 (9.4)	62.0 (9.6)
Male sex, *n* (%)	55 (68.8)	24 (57.1)
Race/ethnicity, *n* (%)		
African	9 (11.3)	2 (4.8)
Asian	2 (2.5)	0(0)
Caucasian	65 (81.3)	40 (95.2)
Hispanic/Latino	1 (1.3)	0(0)
Other/NOS	3 (3.8)	0(0)
Median time from diagnosis to consent, months	4.0	4.5
IMWG response at screening, *n* (%)		
sCR	4 (5.0)	2 (4.8)
CR	12 (15.0)	7 (16.7)
VGPR	33 (41.3)	23 (54.8)
PR	31 (38.8)	10 (23.8)
Median no. of induction cycles (±s.d.)	4 (0.9)	4 (0.8)
Lenalidomide-containing induction, *n* (%)	57 (71.3)	28 (66.7)
Anti-CD38 antibody-containing induction, *n* (%)	0(0)	1 (2.4)
Previous radiotherapy, *n* (%)	9 (11.3)	4 (9.5)

Similar demographics were observed for patients treated with motixafortide + G-CSF (*n* = 80) compared with placebo + G-CSF (*n* = 42). IWMG, International Myeloma Working Group. NOS, not otherwise specified; sCR, stringent complete remission;VGPR, very good partial remission.

### Motixafortide + G-CSF rapidly mobilized high numbers of HSPCs

**Extended Data Fig. 2:**
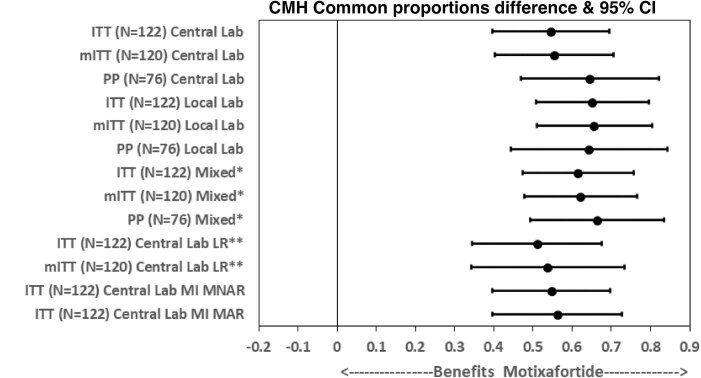
Pre-specified Principal and Sensitivity Analyses of the Primary Endpoint. Local and central lab CD34+ HSPC assessments of the apheresis product from each patient were analyzed for the primary endpoint using Cochran-Mantel-Haenszel (CMH) weights on an intent-to-treat (ITT) basis using N = 122 biologically independent samples examined over 1 experiment/sample. In addition, a pre-specified sensitivity analysis was performed on a modified intent-to-treat (mITT) basis using N = 120 biologically independent samples examined over 1 experiment/sample and on a per protocol (PP) basis using N = 76 biologically independent samples examined over 1 experiment/sample. The data are presented as CMH common proportions differences for each analysis with 2-sided 95% confidence intervals (CI) indicated by the error bars. Mixed* analyses accounted for missing central lab values by replacing them with non-missing local lab values. Logistic regression (LR**), multiple imputation missing not at random (MI MNAR) and missing at random (MI MAR) analyses were also performed.

Enumeration of CD34^+^ HSPCs in the apheresis product was performed by both local and central laboratories. According to the prespecified protocol, all clinical decisions were made based on local laboratory results, including the determination that the patient met the collection goal for the primary endpoint and determining the number of CD34^+^ cells kg^–1^ infused for ASCT. Local and central laboratory assessments were included in the prespecified statistical analysis plan, with statistically significant results observed via both assessments favoring the increased effectiveness of motixafortide + G-CSF compared with placebo + G-CSF (Extended Data Fig. 2).

**Fig. 2:**
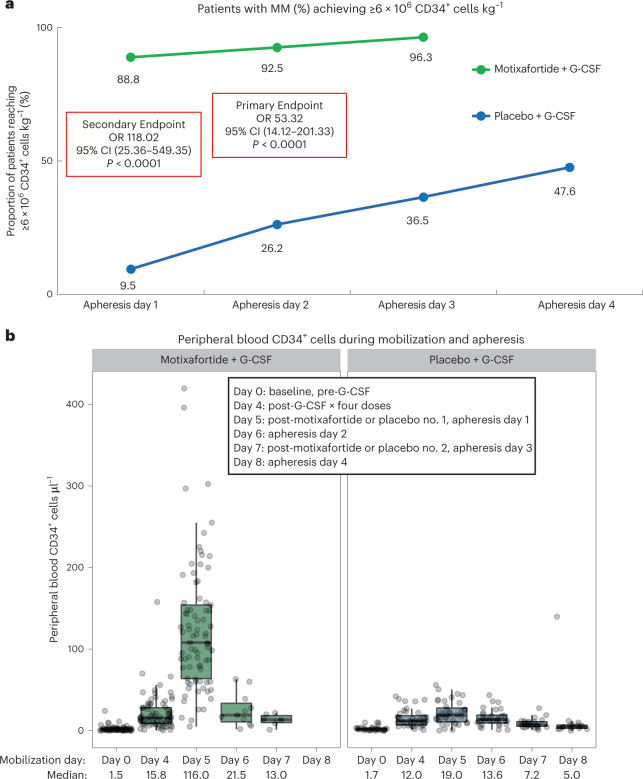
GENESIS trial efficacy. **a**,**b**, Patients with MM were mobilized with either motixafortide + G-CSF or placebo + G-CSF with the goal of collecting ≥6 × 10^6^ CD34^+^ cells kg^–1^. **a**, The proportion of patients meeting the primary endpoint of collecting to goal within two apheresis days and the key secondary endpoint of collecting to goal in one apheresis day are shown, along with collection rates after three and four apheresis procedures. No patients in the motixafortide + G-CSF arm underwent a fourth apheresis on protocol. The primary endpoint and prespecified secondary efficacy endpoint were analyzed using the CMH test and are presented as OR with two-sided 95% CIs and *P* values. **b**, The numbers of PB CD34^+^ cells μl^–1^ in each cohort are presented by mobilization day with standard box and whisker plots, where the measure of center is the median value for PB CD34^+^ cells μl^–1^ with the exact median value of CD34^+^ cells μl^–1^ noted below the *x* axis by day and cohort; the box represents the interquartile range and lines represent the minimum/maximum (±1.5 × interquartile range). Each patient contributed a single biologically independent sample examined over one independent experiment per sample/patient. Total sample counts associated with motixafortide + G-CSF were: day 0 (*n* = 80), day 4 (*n* = 80), day 5 (*n* = 80), day 6 (*n* = 11), day 7 (*n* = 6) and day 8 (*n* = 0). Total sample counts associated with placebo + G-CSF were: day 0 (*n* = 39), day 4 (*n* = 40), day 5 (*n* = 39), day 6 (*n* = 32), day 7 (*n* = 27) and day 8 (*n* = 17).

Mobilization with motixafortide + G-CSF resulted in 92.5% of patients meeting the primary endpoint of collecting ≥6 × 10^6^ CD34^+^ cells kg^–1^ within two apheresis days versus 26.2% with placebo + G-CSF, by local laboratory assessment using an intention-to-treat (ITT) analysis (OR 53.3, 95% CI 14.12–201.33, *P* < 0.0001) (Fig. 2a). Furthermore, 88.8% of patients mobilized with motixafortide + G-CSF met the key prespecified secondary endpoint of collecting ≥6 × 10^6^ CD34^+^ cells kg^–1^ in one apheresis day versus 9.5% with placebo + G-CSF (OR 118.0, 95% CI 25.36–549.35, *P* < 0.0001).

The baseline level of PB CD34^+^ HSPCs before G-CSF administration was similar between the motixafortide + G-CSF and placebo + G-CSF cohorts, at 1.5 and 1.7 CD34^+^ cells µl^–1^, respectively (Fig. 2b). Following four doses of G-CSF and before motixafortide or placebo administration, PB CD34^+^ counts remained similar between the motixafortide + G-CSF and placebo + G-CSF cohorts, at 15.8 and 12.0 CD34^+^ cells µl^–1^, respectively. However, following administration of either motixafortide or placebo but before apheresis, the median number of PB CD34^+^ HSPCs in the motixafortide + G-CSF cohort (*n* = 74) was 116.0 versus 19.0 cells µl^–1^ in the placebo + G‑CSF cohort (*n* = 40) (*P* < 0.001), with PB CD34^+^ cells µl^–1^ correlating well with apheresis yields.

Meanwhile, 96.3% of patients mobilized with motixafortide + G-CSF collected ≥2 × 10^6^ CD34^+^ cells kg^–1^ within one apheresis day versus 64.3% with placebo + G-CSF (OR 18.9, 95% CI 4.47–80.04, *P* < 0.0001). The median number of CD34^+^ HSPCs mobilized in one apheresis day with motixafortide + G-CSF was 10.8 × 10^6^ versus 2.25 × 10^6^ cells kg^–1^ with placebo + G-CSF. The total number of CD34^+^ HSPCs infused for ASCT was determined independently by each investigator according to local practice, with a median of <6 × 10^6^ cells kg^–1^ infused for ASCT in both arms (minimum 2 × 10^6^ CD34^+^ cells kg^–1^ required according to the protocol). Median time to neutrophil engraftment (TNE) was 12 days in both arms (hazard ratio (HR) not estimable, *P* = 0.9554). Median time to platelet engraftment (TPE) was 18 days with motixafortide + G-CSF and 17 days with placebo + G-CSF (HR 0.95, 95% CI 0.2–5.7, *P* = 0.9554). Graft durability at day 100 post ASCT was 92.2% in the motixafortide + G-CSF arm and 91.9% in the placebo + G-CSF arm (OR 1.04, 95% CI 0.2–4.5, *P* = 0.96). Progression-free survival (PFS) and overall survival (OS) were comparable between the two cohorts and consistent with contemporary outcomes (Extended Data Table 2).

### Motixafortide + G-CSF was safe and well tolerated

Treatment-emergent AEs (TEAEs) were defined as starting with the first dose of G-CSF through 30 days following the last apheresis procedure or the first dose of conditioning chemotherapy, whichever occurred first. Overall, TEAEs were reported in 93.8% (grade 3, 27.5%) of patients with motixafortide + G-CSF versus 83.3% (grade 3, 4.8%) with placebo + G-CSF (Table 2). The most common TEAEs related to motixafortide were transient, grade 1/2 local injection site reactions and systemic reactions. Local injection site reactions most commonly included pain (50%), erythema (27.5%) and pruritis (21.3%). Systemic reactions most commonly included flushing (32.5%), pruritis (33.8%), urticaria (12.5%) and erythema (12.5%). Meanwhile, bone pain was commonly observed in the placebo + G-CSF arm (31.0%). No grade 4 TEAEs or deaths occurred during the mobilization period of the study.

**Table 2:** GENESIS trial safety and toxicity

TEAEs (frequency >10%)	Motixafortide + G-CSF		Placebo + G-CSF	
	Any grade	Grade 3	Any grade	Grade 3
Total, % (*n*)	93.8 (75 of 80)	27.5 (22 of 80)	83.3 (35 of 42)	4.8 (2 of 42)
Local injection site reactions, % (*n*)				
Pain	50 (40 of 80)	6.3 (5 of 80)	4.8 (2 of 42)	0
Erythema	27.5 (22 of 80)	0	0	0
Pruritis	21.3 (17 of 80)	0	0	0
Systemic injection reactions, % (*n*)				
Flushing	32.5 (26 of 80)	7.5 (6 of 80)	0	0
Pruritis	33.8 (27 of 80)	11.3 (9 of 80)	0	0
Urticaria	12.5 (10 of 80)	1.3 (1 of 80)	0	0
Erythema	12.5 (10 of 80)	0	0	0
Other, % (*n*)				
Bone pain	17.5 (14 of 80)	0	31.0 (13 of 42)	0
Back pain	17.5 (14 of 80)	0	14.3 (6 of 42)	0
Nausea	13.8 (11 of 80)	0	11.9 (5 of 42)	0
Hypokalemia	13.8 (11 of 80)	0	11.9 (5 of 42)	0
Catheter site pain	11.3 (9 of 80)	0	14.3 (6 of 42)	0

All TEAEs occurring at a frequency of >10% in either motixafortide + G-CSF or placebo + G-CSF during the period from the first dose of G-CSF through 30 days following the last apheresis procedure or the first dose of conditioning chemotherapy, whichever occurred first. No grade 4 TEAEs or deaths events occurred during this period.

### Motixafortide + G-CSF reduced healthcare resource utilization

As a prespecified analysis, healthcare resource utilization was assessed comparing motixafortide + G-CSF with placebo + G-CSF. Patients mobilized with motixafortide + G-CSF received 5.26 G-CSF injections per patient versus 8.12 in the placebo + G-CSF cohort (*P* < 0.0001). Additionally, patients mobilized with motixafortide + G-CSF required an average of 1.23 apheresis procedures per patient to mobilize optimal CD34^+^ HSPCs versus 3.24 with placebo + G-CSF (*P* < 0.0001). No patients mobilized with motixafortide + G-CSF underwent a fourth day of apheresis for primary mobilization and only 1% (*n* = 1) required remobilization, whereas 63.5% of patients mobilized with placebo + G-CSF required a fourth day of apheresis and 23.8% (*n* = 10) required remobilization with plerixafor + G-CSF.

### Motixafortide + G-CSF mobilized high numbers of primitive HSCs

**Fig. 3:**
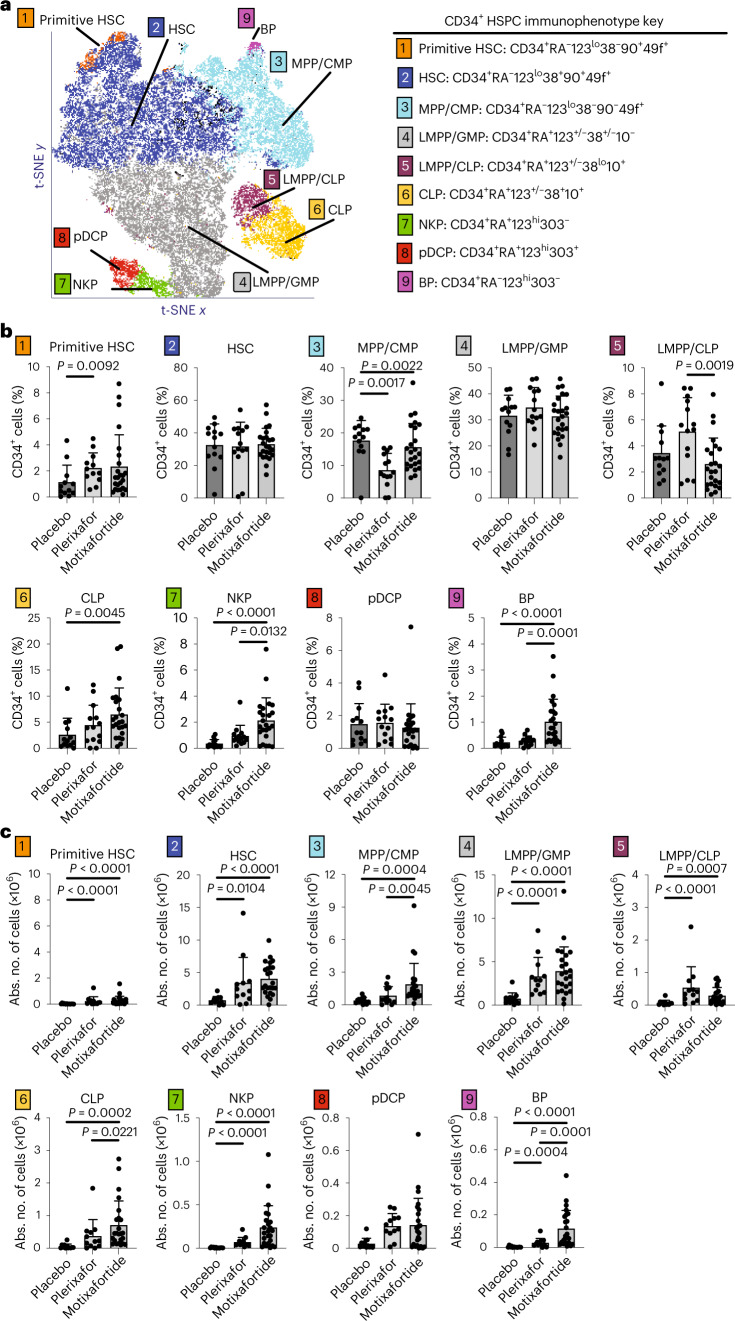
Immunophenotyping with percentages and quantitation of CD34^+^ HSPC subsets. **a**–**c**, CD34^+^ HSPCs from day 1 apheresis products, collected following treatment of patients with MM with G-CSF plus either placebo, plerixafor or motixafortide, were purified by immunomagnetic selection and evaluated by multicolor FACS. Each patient (*n* = 51) contributed a single biologically independent sample examined over one independent experiment per sample/patient. **a**, *t*-Distributed stochastic neighbor-embedding (t-SNE) projection of merged flow cytometry file showing nine HSPC subsets based on defined cell surface markers. **b**, Percentage of CD34^+^ cells within each HSPC subset is shown for patients with MM treated with G-CSF plus either placebo (*n* = 13), plerixafor (*n* = 14) or motixafortide (*n* = 24). **c**, Absolute numbers (Abs. no.) of each HSPC subset are shown for patients with MM treated with G-CSF plus either placebo (*n* = 12), plerixafor (*n* = 12) or motixafortide (*n* = 24). **b**,**c**, Data presented as mean ± s.d. Mean HSPC subset yields were compared using ANOVA followed by a post hoc Tukey–Kramer test for pairwise comparisons among groups. Exact two-sided *P* values for significant differences are listed.

**Extended Data Fig. 3:**
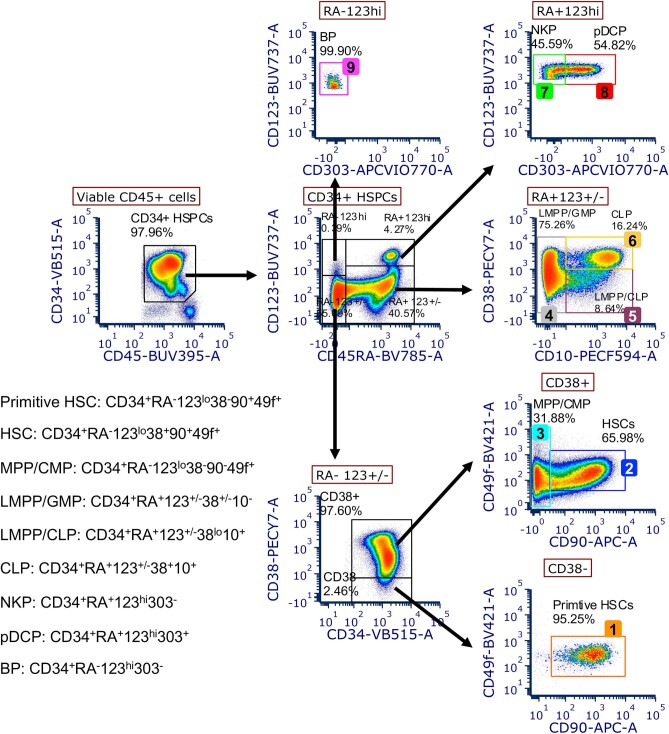
Flow Cytometry Gating Strategy. CD34^+^ HSPCs from day 1 apheresis products collected following treatment of patients with MM with G-CSF plus either placebo, plerixafor or motixafortide were purified by immunomagnetic selection and evaluated by multicolor FACS. Gating strategy used to define nine different CD34+ HSPC subsets is shown.

Extended immunophenotyping via multicolor FACS of CD34^+^ HSPCs from the day 1 apheresis product of patients (*n* = 51) mobilized with placebo + G-CSF (*n* = 13), plerixafor + G-CSF (*n* = 14) and motixafortide + G-CSF (*n* = 24) as a prespecified correlative analysis demonstrated nine distinct CD34^+^ HSPC subsets, ranging from primitive HSCs to lineage-committed progenitors (Fig. 3a and Extended Data Fig. 3). Compared with placebo + G-CSF, motixafortide + G-CSF significantly increased percentages of common lymphoid progenitors (CLPs), natural killer cell precursors (NKPs) and basophil precursors (BPs) (Fig. 3b). When compared with plerixafor + G-CSF, motixafortide + G-CSF significantly increased percentages of multipotent progenitors and common myeloid progenitors (MPPs/CMPs), NKPs and BPs, with fewer lymphomyeloid primed progenitors (LMPPs/CLPs) (Fig. 3b). Quantitation of absolute numbers of HSPC subset yields demonstrated significantly increased quantities of eight out of nine HSPC subsets in the motixafortide + G-CSF products versus placebo + G-CSF, with 10.5-fold higher absolute numbers of primitive HSCs (Fig. 3c). Compared with plerixafor + G-CSF, motixafortide + G-CSF significantly increased numbers of MPPs/CMPs, CLPs and BPs (Fig. 3c). Taken together, these data suggest that motixafortide induced pan-mobilization of multiple HSPC subsets capable of broad multilineage hematopoietic reconstitution, with notable increases in the absolute number of primitive HSCs and MPPs/CMPs.

**Extended Data Fig. 4:**
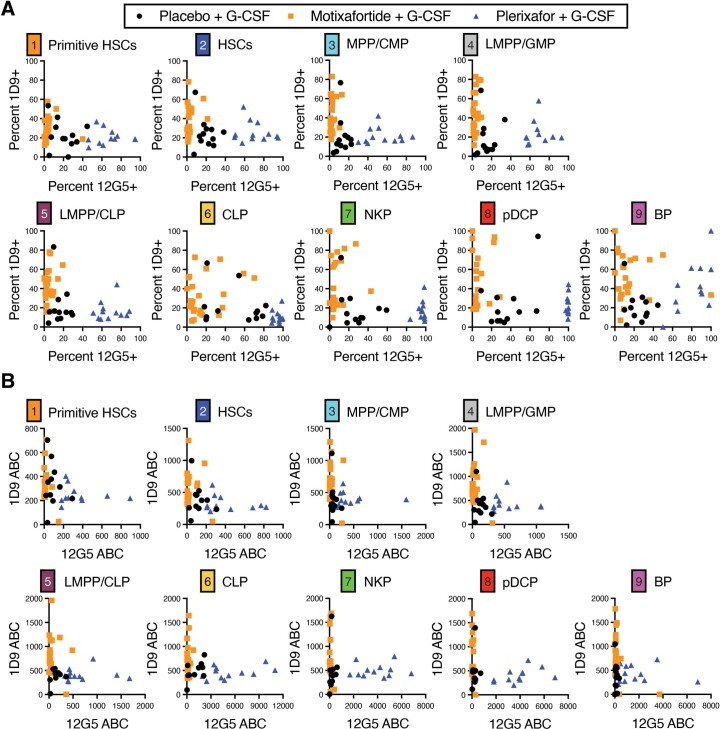
CXCR4 Expression on CD34+ HSPC Subsets from Day 1 of Apheresis. CD34^+^ HSPCs from day 1 apheresis products collected following treatment of patients with MM with G-CSF and either placebo (n = 12), plerixafor (n = 12) or motixafortide (n = 24) were purified by immunomagnetic selection and evaluated by flow cytometry. The expression of CXCR4 was determined by flow cytometry using anti-CXCR4 mAb clones 12G5 and 1D9. (**A**) Percent of HSPC subsets positive for 12G5+ and 1D9+ antibodies. (**B**) Mean Fluorescence Intensity (MFI) of antibody binding capacity (ABC) of 12G5 and 1D9 on HSPC subsets.

CD34^+^ HSPCs from apheresis were split and stained with two different antibodies to CD184 (CXCR4), clones 12G5 and 1D9. The 12G5 antibody recognizes an epitope involving the first and second extracellular domains of CXCR4 and competes with motixafortide and plerixafor for CXCR4 binding^23,24^. In contrast, the 1D9 antibody binds the N terminus of CXCR4 and is unaffected by motixafortide or plerixafor bound to CXCR4 (ref. ^24^). Binding of 1D9 to CD34^+^ HSPCs was similar among all three arms (*P* = 0.45–0.75). In contrast, both the percentage of 12G5^+^ cells and total 12G5 antibody-binding capacity were significantly lower in the motixafortide + G-CSF cohort versus placebo + G-CSF (*P* < 0.0001) and plerixafor + G-CSF (*P* < 0.0001) for all HSPC subsets, consistent with extended CXCR4 occupancy by motixafortide (Extended Data Fig. 4). Despite extended CXCR4 occupancy by motixafortide, there was no impact on rehoming of HSPCs to the bone marrow with comparable engraftment kinetics and graft durability (Extended Data Table 2). These findings are consistent with previously reported data, and may be due to inhibition by motixafortide of CXCL12-induced CXCR4 internalization in a dose-dependent manner, leading to extended CXCR4 half-life on the cell surface and ultimately net upregulation of CXCR4 expression^20,25^.

### HSPC number and subsets infused impact engraftment

The number of CD34^+^ cells kg^–1^ infused for ASCT was determined independently by each investigator according to local practice, with a median of <6 × 10^6^ CD34^+^ cells kg^–1^ infused in both cohorts and similar TPE and TNE (Extended Data Table 2).

**Extended Data Fig. 5:**
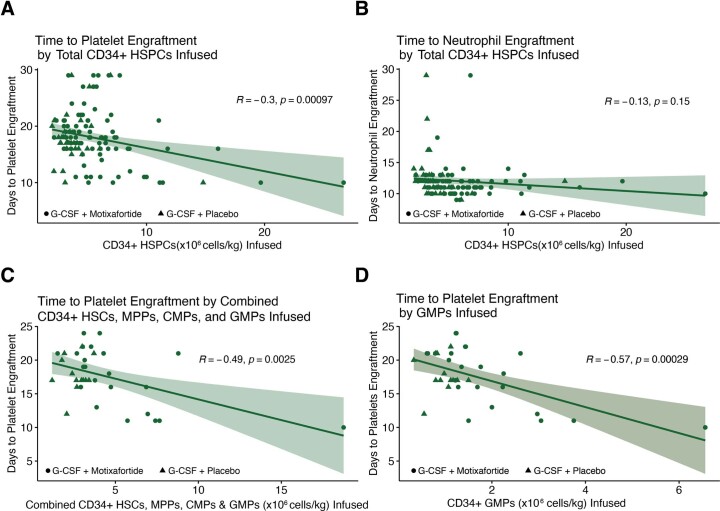
Engraftment Kinetics by Total CD34+ HSPCs Infused and by Specific CD34+ HSPCs Subsets Infused. Pearson correlation with green line representing the regression curve and the shaded green area representing the 2-sided 95% CI presented with respective p-values. All statistical analyses were 2-sided. (**a**) time to platelet engraftment in days by total CD34+ HSPCs infused, (**b**) time to neutrophil engraftment in days by total CD34+ HSPCs infused, (**c**) time to platelet engraftment in days by total combined HSC, MPP, CMP and GMP CD34+ subset HSPCs infused and (**d**) time to platelet engraftment in days by total GMP CD34+ subset HSPCs infused. Each motixafortide+G-CSF patient indicated by circles and each placebo+G-CSF patient indicated by triangles.

However, a post hoc pooled analysis of all patients (*n* = 114) revealed an inverse correlation between increasing levels of CD34^+^ cells kg^–1^ infused and TPE (*R* = −0.30, *P* = 0.00097; Extended Data Fig. 5a). Sensitivity analyses determined that infusion of ≥7 × 10^6^ CD34^+^ cells kg^–1^ was associated with faster median TPE of 14 versus 18 days with <7 × 10^6^ CD34^+^ cells kg^–1^ (HR 0.57, 95% CI 0.34–0.94, *P* = 0.0276). A dose–response relationship was also observed, including (1) TPE of 11 days with ≥8 × 10^6^ CD34^+^ cells kg^–1^ infused versus 18 days with <8 × 10^6^ CD34^+^ cells kg^–1^ (HR 0.28, 95% CI 0.15–0.53, *P* = 0.0001) and (2) TPE of 10 days with ≥9 × 10^6^ CD34^+^ cells kg^–1^ infused versus 18 days with <9 × 10^6^ CD34^+^ cells kg^–1^ (HR 0.28, 95% CI 0.14–0.57, *P* = 0.0004). TNE was not impacted by total CD34^+^ cells kg^–1^ infused (*R* = −0.13, *P* = 0.15) (Extended Data Fig. 5b).

In addition, a post hoc pooled analysis of 37 patients (motixafortide + G-CSF, *n* = 24; placebo + G-CSF, *n* = 13) with extended CD34^+^ immunophenotyping via multicolor FACS revealed that infusion of higher numbers of combined CD34^+^ HSC, MPP, CMP and granulocyte monocyte progenitor (GMP) subsets was associated with more rapid TPE (*R* = −0.49, *P* = 0.0025) (Extended Data Fig. 5c). Also, infusion of higher numbers of GMPs alone was associated with more rapid TPE (*R* = −0.57, *P* = 0.00029) (Extended Data Fig. 5d). Sensitivity analyses determined that infusion of higher numbers (>75th percentile) of GMPs was specifically associated with more rapid TPE of 13 versus 19 days with lower numbers of GMPs (*P* = 0.0116). TNE was not impacted by specific CD34^+^ HSPC subsets infused (all *P* > 0.05).

### Motixafortide mobilized transcriptionally primitive HSPCs

Single-cell transcriptional profiling was performed via scRNA-seq on CD34^+^ HSPCs from the day 1 apheresis products of patients with MM (*n* = 12) mobilized with placebo + G-CSF (*n* = 4), plerixafor + G-CSF (*n* = 4) and motixafortide + G-CSF (*n* = 4), along with CD34^+^ HSPCs from the apheresis product of healthy allo-donors (*n* = 6) mobilized with G-CSF alone (*n* = 2), plerixafor alone (*n* = 2) and motixafortide alone (*n* = 2). When compared with the MM cohorts, allo-donor patients were generally younger (median age of MM cohort, 62–68 years versus allo-donor cohort, 55 years) and lacked a history of MM or recent chemotherapy exposure. Otherwise, the cohorts were demographically similar.

**Fig. 4:**
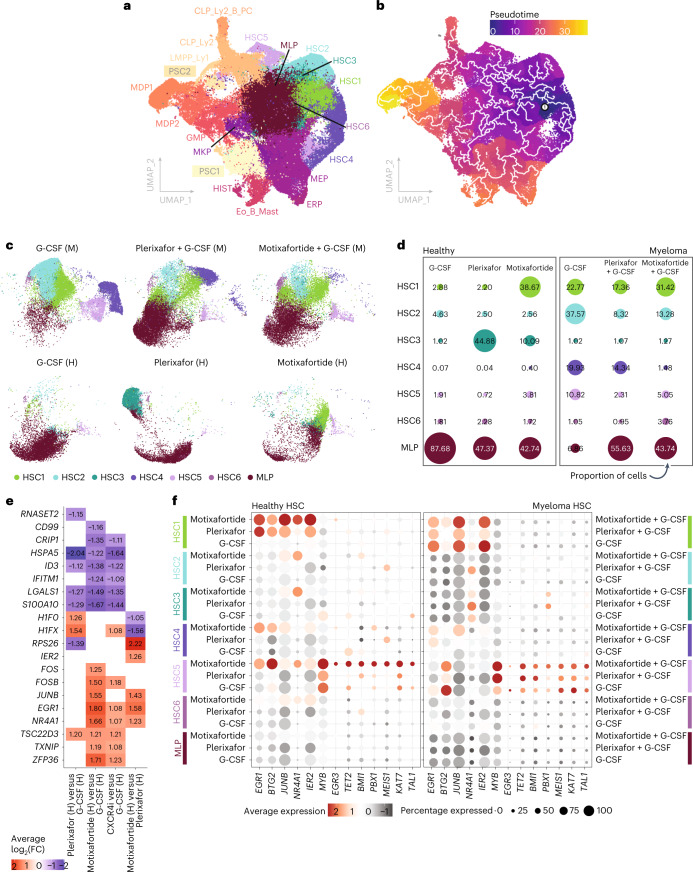
Single-cell transcriptional profiling and trajectory mapping of CD34^+^ HSPCs with differentially expressed gene subanalysis of HSC1–6 and MLP populations. **a**, UMAP plot of annotated single-cell clusters across entire cohort. Cells colored by abbreviations of each transcriptional annotation as defined. **b**, UMAP plot of single-cell clusters with Monocle3 trajectory mapping overlayed and colored by pseudotime. Overlayed trajectory indicates transitions between distinct transcriptional states. Pseudotime is a measure of progress along the overlayed trajectory, with the white spot labeled ‘1’ indicating the earliest state on the transcriptional path. **c**, UMAP of early progenitor populations (HSC1–6, MLP) across each cohort and mobilization regimen, with each cell colored by cell-type annotation and separated by mobilization regimen and cohort (M, multiple myeloma; H, healthy allo-donor). **d**, Average cell-type proportions across each sample within each treatment group and cohort. Spot size indicates relative average expression, with the exact value overlayed. **e**, Highlighted genes found to be differentially expressed between early progenitor populations in the healthy allo-donor cohort across the listed mobilization regimens. Each column represents a different DEG analysis between the two groups, and values for each DEG listed show the average log_2_(fold change (FC)) in the first cohort relative to the second cohort in each column. Each element in heatmap is colored by average log_2_(FC), with red and blue denoting increased and decreased expression, respectively. **f**, Expression of a subset of DEGs and genes (from the literature) across stem cell and early progenitor clusters. Each spot is colored by average expression, its size indicating total number of cells expressing the gene of interest in the mobilization regimen and cohort labeled.

**Extended Data Fig. 6:**
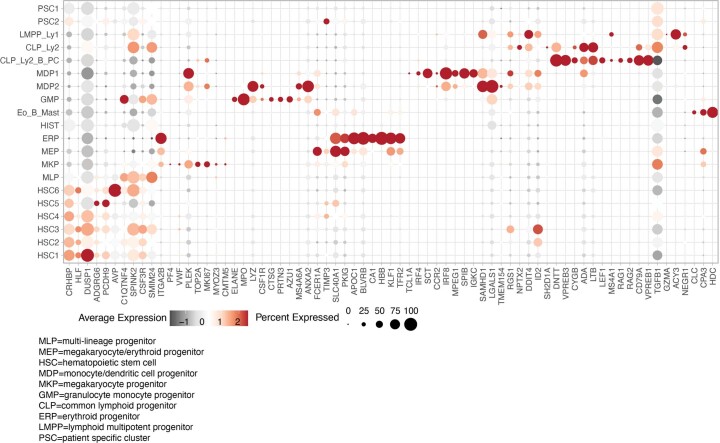
Gene Markers for CD34+ Cell Type Assignments. Cell type markers from the literature used to annotate single-cell clusters. Dot plot shows the average expression of each gene for each cell-type population and size shows the percent of cells expressing that gene of interest.

A total of 144,982 purified CD34^+^ HSPCs were sequenced across all 18 samples (range 2,086–20,062 cells per patient, average 2,767 genes per cell). Uniform manifold approximation and projection (UMAP) clustering was performed, with cell identity determined by cross-referencing of gene expression profiles with previously published datasets and genes reported as reliable markers of lineage commitment^9,26**–**29^. A total of 20 transcriptionally distinct CD34^+^ HSPC subsets were identified, including (1) six transcriptionally unique HSC subclusters (HSC1–6), (2) a large multilineage progenitor (MLP) cluster and (3) ten lineage-biased lymphoid (CLP_Ly1/2), myeloid (GMP), monocytic/dendritic (MDP1/2), megakaryocytic/erythrocytic (MEP, MKP and ERP), histiocytic (HIST) and eosinophil/basophil/mast cell (Eo_B_Mast) progenitors (Fig. 4a and Extended Data Fig. 6). Transcriptional trajectory analysis over pseudotime predicted the HSC1 population as the most transcriptionally primitive HSC population, differentiating into HSC2–6 as well as MLP. Subsequent lineage-biased populations appear to differentiate from HSC2–6 and MLP (Fig. 4b).

**Extended Data Fig. 7:**
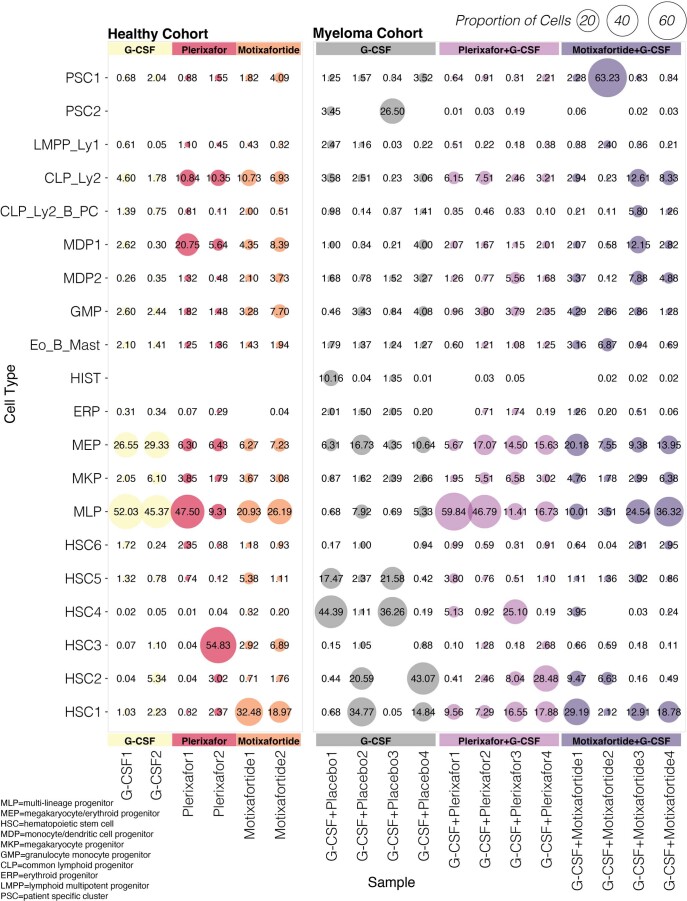
Transcriptionally Defined HSPC Subsets with Distribution by Cohort and Mobilization Regimen. Average cell-type proportions of each transcriptionally defined HSPC subset annotated by sample. Samples are grouped by mobilization regimen and cohort.

The proportion of each transcriptionally defined HSPC cluster in healthy allo-donors (mobilized with G-CSF, plerixafor or motixafortide alone) and in patients with MM (mobilized with placebo + G-CSF, plerixafor + G-CSF or motixafortide + G-CSF) was evaluated (Extended Data Fig. 7). Notably, healthy allo-donors receiving motixafortide alone mobilized a higher proportion of transcriptionally primitive HSC1 cells (25.73%) relative to both plerixafor (1.35%) and G-CSF (1.63%). In addition, allo-donors receiving CXCR4i mobilization with either motixafortide or plerixafor mobilized a higher proportion of MDP1 and MDP2 cells relative to those mobilized with G-CSF alone (MDP1, G-CSF 1.46% versus CXCR4i 9.78% and MDP2, G-CSF 0.31% versus CXCR4i 1.98%). By contrast, within the MM cohorts similar proportions of HSC2–6, CLPs and GMPs were mobilized across the three mobilization regimens. However, the large proportion of MLPs mobilized by all regimens in the allo-donor cohorts (33.56%) was notably lower in patients with MM mobilized with placebo + G-CSF (3.66%). However, this large population of MLPs was preserved when either motixafortide or plerixafor was added to G-CSF mobilization in patients with MM (26.18%), suggesting that MLPs may be uniquely reduced in the G-CSF mobilized HSPC graft of patients with MM and that the addition of CXCR4i with either motixafortide or plerixafor to G-CSF may preserve this population within the HSPC graft in those patients. Taken as a whole, these data suggest that the mobilization regimen used in both allo-donors and patients with MM has an important impact on the proportion of various HSPC subsets mobilized. In addition, the presence of underlying MM, recent exposure to MM-targeting therapies, advanced age or a combination of factors may further impact mobilization of particular HSPC subsets.

Differential gene expression (DEG) analysis across all subsets identified unique transcriptional profiles for each CD34^+^ HSPC subset, with specific differences based on mobilization regimen (motixafortide versus plerixafor) and donor type (allo-donor versus patient with MM). Within the HSC1–6 and MLP populations in healthy allo-donors, the HSC1 population was preferentially mobilized at higher numbers by motixafortide compared with either plerixafor or G-CSF (Fig. 4c,d). When comparing motixafortide with G-CSF or plerixafor-mobilized HSCs in the allo-donor cohort, we found *EGR1*, *JUNB*, *NR4A1*, *IER2* and *RPS26* to be notably upregulated, which has been associated with enhanced quiescence and self-renewal (Fig. 4e,f)^30–32^. By plotting the expression of these markers relative to single-cell populations, most of the expression of these genes is driven by HSC1 and HSC5. Moreover, these genes have markedly higher expression in HSCs mobilized by the long-acting CXCR4i motixafortide relative to either the short-acting CXCR4i plerixafor or G-CSF alone. In addition, *KAT7* (HBO1), *BMI1*, *PBX1* and *MEIS1* and a related network of genes, which have been associated with HSC maintenance, quiescence and self-renewal, were highly expressed in the HSC5 population mobilized by motixafortide relative to plerixafor or G-CSF^28^. Similarly, HSC2–6 cells mobilized in allo-donors with motixafortide, but not plerixafor or G-CSF, expressed increased levels of *JUNB* and *NR4A1*. By contrast, gene expression profiles of the HSC1–6 and MLP populations were more homogenous within the MM cohorts, potentially reflecting the impact of recent MM-targeting induction therapies on the bone marrow niche, as well as increased age and/or a pre-existing diagnosis of MM. Nevertheless, motixafortide + G-CSF preferentially mobilized a higher proportion of the transcriptionally primitive HSC1 population (31.42%) relative to either plerixafor + G-CSF (17.36%) or placebo + G-CSF (22.77%) in patients with MM.

**Extended Data Fig. 8:**
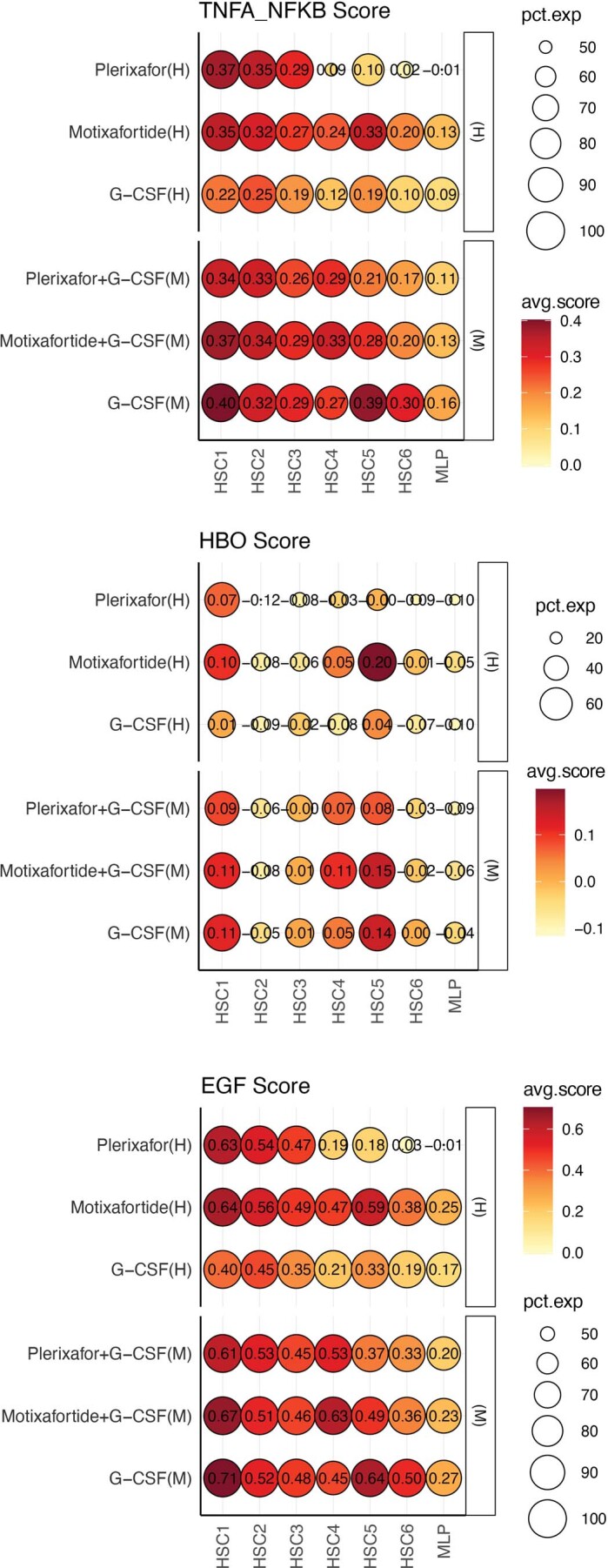
TNFα/NFκB, EGF and HBO1 (KAT7) Gene Scores. For three different pathways a list of genes was curated, derived from GSEA and the literature, to evaluate changes in expression of related pathways (Methods). Each Gene Score dot plot shows the average gene expression score indicated by heat map color and overlayed with the numerical score. Dot size indicates the percentage of cells expressing each transcriptional program. Each plot is also separated by mobilization regimen (left axis); by multiple myeloma (M) and healthy allo-donor cohorts (H) (right axis); and by the HSC1-6 or MLP population of interest (bottom axis).

Gene set enrichment analysis of upregulated genes in the primitive HSC1 population relative to other HSC/MLP populations indicated increased EGF- and TNF-α/NFκB-related signaling, which have been associated with hematopoietic regeneration and/or regenerative potential. Meanwhile, HSC5 expressed upregulated HBO1-related genes associated with self-renewal and quiescence relative to other HSC/MLP populations. To further evaluate EGF, TNF-α/NFκB and HBO1 pathway signaling within each HSC1–6 and MLP subset across patient/treatment/disease characteristics (allo-donor versus MM) and mobilization regimen, pathway expression scores were generated for each pathway (Extended Data Fig. 8 and Methods). These data demonstrated numerically higher average levels of EGF-, TNF-α/NFκB- and HBO1-related gene expression with the use of the long-acting CXCR4i motixafortide as compared with short-acting CXCR4i with plerixafor or G-CSF alone, within most HSC1–6 and MLP subsets in healthy allo-donors, whereas the differences in gene expression scores for EGF-, TNF-α/NFκB- and HBO1-related genes in HSC1–6 and MLP subsets in the MM cohorts were relatively similar across mobilization regimens. In summary, extended CXCR4i with motixafortide mobilizes HSC populations, with upregulated gene expression profiles associated with enhanced self-renewal, quiescence and regeneration in healthy allo-donors.

## Discussion

The effectiveness of ASCT relies, in part, on the ability to collect an adequate number of HSPCs. The ‘ideal’ HSPC mobilization regimen would be rapid, robust, reliable, well tolerated and capable of safely mobilizing an optimal number of CD34^+^ HSPCs in nearly 100% of patients in one apheresis day. Although G-CSF remains the most widely used mobilization agent, 40–50% of patients remain unable to collect an optimal number of HSPCs with G-CSF alone despite multiple injections and up to four apheresis days^11,12^. In addition, 15–35% of patients remain unable to collect optimal numbers of cells despite up to eight injections of G-CSF, four injections of plerixafor and four apheresis days^12,18^. Therefore, the ideal mobilization regimen remains elusive. In addition, progressive improvements in transplant-related care have enabled ASCT to be safely performed in patients with MM ≥65 years of age, increasing from 11% of ASCTs in 2000 to 36% of ASCTs in 2019 (ref. ^33^). Similarly, advances in induction therapy have established three-drug (IMiDs, PIs and glucocorticoids) and four-drug induction regimens (IMiD, PI, glucocorticoids and anti-CD38 mAbs) as the standard of care for newly diagnosed transplant-eligible patients with MM^34,35^. However, increased age, exposure to lenalidomide and four-drug induction therapy are all associated with impaired HSPC mobilization, further emphasizing the unmet need for more effective HSPC mobilization regimens^36–38^.

In this international, phase 3, multicenter, randomized, double-blind, placebo-controlled trial, patients were representative of the typical MM population undergoing ASCT in the current era, with a median age of 63 years and 70% of patients receiving lenalidomide-containing induction therapy. Despite the presence of these risk factors for poor HSPC mobilization, 92.5% of patients treated with one injection of motixafortide + G-CSF collected ≥6 × 10^6^ CD34^+^ cells kg^–1^ within two apheresis days versus 26.2% mobilized with placebo + G-CSF (*P* < 0.0001). In addition, 88.8% of patients mobilized with motixafortide + G-CSF collected ≥6 × 10^6^ CD34^+^ cells kg^–1^ in one apheresis day versus 9.5% with placebo + G-CSF (*P* < 0.0001), with a median of 10.8 × 10^6^ CD34^+^ cells kg^–1^ collected in one apheresis with motixafortide + G-CSF. By comparison, in a contemporary cohort of demographically similar patients with MM prospectively enrolled in parallel to the GENESIS trial and mobilized with plerixafor + G-CSF, 50.0% of patients collected optimal numbers of CD34^+^ cells kg^–1^ in one apheresis (median, 5.47 × 10^6^ CD34^+^ cells kg^–1^). Meanwhile, motixafortide + G-CSF was well tolerated with rapid and durable engraftment kinetics. Motixafortide + G-CSF as upfront HSPC mobilization also significantly reduced both G-CSF usage and the number of apheresis days required to collect the optimal number of HSPCs.

Randomized controlled trials (RCTs) comparing the efficacy of G-CSF alone, chemotherapy + G-CSF, plerixafor + G-CSF and motixafortide + G-CSF to mobilize optimal HSPC numbers (≥6 × 10^6^ CD34^+^ cells kg^–1^) in the current era of MM therapy are lacking. In addition, comparisons between the present study and previous trials should be undertaken with caution, acknowledging the limitations of cross-trial comparisons. The largest previous RCT comparing plerixafor + G-CSF with placebo + G-CSF in patients with MM was published in 2009, with a median patient age of 58 years and only 5.9% of patients receiving lenalidomide before HSPC mobilization. In the context of relatively younger patients with minimal lenalidomide exposure compared with current practice, 17.3% of patients in the placebo + G-CSF arm and 54.2% in the plerixafor + G-CSF arm mobilized to goal in one apheresis^12^. Other contemporary studies commonly utilize pre-apheresis PB CD34^+^ counts to screen for poor mobilizers, and add pre-emptive plerixafor mobilization in patients predicted to fail mobilization with G-CSF alone, making direct comparisons difficult^39,40^. Meanwhile, other studies targeted lower mobilization goals (≥2–3 × 10^6^ CD34^+^ cells kg^–1^), limited premobilization lenalidomide exposure, used alternative growth factors or allowed more than four apheresis days to collect to goal, again making direct comparisons difficult^13,41,42^. Nevertheless, most RCTs and nonrandomized interventional trials in patients with MM since the development of CXCR4is for HSPC mobilization have reported that currently available regimens frequently yield suboptimal CD34^+^ numbers despite multiple injections and multiple days of apheresis^12,13,39,40^. Thus it appears, based on these data, that a single injection of motixafortide added to G-CSF substantially improves on currently approved mobilization regimens in terms of the rapidity, robustness and reliability of HSPC mobilization for ASCT in patients newly diagnosed with MM following modern induction therapy in the current era.

There is also an increasing appreciation of the immunophenotypic and transcriptional heterogeneity of CD34^+^ HSPCs, ranging from primitive HSCs capable of long-term self-renewal and multilineage potential down to differentiated, lineage-committed progenitors^19^. Moreover, the impact of extended CXCR4i with motixafortide relative to short-acting CXCR4i with plerixafor on graft composition remains incompletely understood. Previous studies evaluating PB HSPCs mobilized by plerixafor compared with those mobilized by G-CSF or collected from bone marrow in allo-donors demonstrated that plerixafor increased mobilization of strongly CXCR4^+^, lineage-committed plasmacytoid dendritic cell progenitors (pDCPs) comprising nearly 25% of the HSPC graft^19^. We observed that motixafortide mobilized a similar proportion of pDCPs compared with plerixafor; but also mobilized a significantly higher number of combined HSCs, MPPs and CMPs compared with plerixafor. This unique impact of extended CXCR4i with motixafortide relative to short-acting CXCR4i with plerixafor may be due to the observation that these more primitive HSPC subsets are less strongly CXCR4^+^ at baseline, and thus are preferentially mobilized at higher numbers by extended CXCR4i with motixafortide. Also of note, scRNA-seq suggests that extended CXCR4i with motixafortide in healthy allo-donors mobilizes transcriptionally unique subsets of HSCs (HSC1 and HSC5; Fig. 4), which exhibit upregulated transcriptional programing associated with enhanced self-renewal, regeneration and quiescence (*EGR1*, *JUNB*, *BTG2*, *NR4A1*, *MYB*, *IER2*, *EGR3*, *BMI1*, *PBX1*, *MEIS1* and *KAT7* (HBO1)). Recent work by Desterke et al. revealed that quiescence markers (*EGR1*, *JUNB, BTG2* and *NR4A1*) and other genes (*MYB* and *IER2*) were upregulated in long-lived HSCs^30^. The self-renewal transcription factor *EGR3*, which suppresses cell cycle progression, is upregulated in motixafortide-treated clusters and is also highly expressed in primitive HSCs during leukemic transformation^31,32^. Shepard et al. observed that HSCs in JAK2 mutant myeloproliferative neoplasms harbored defective self-renewal properties, while robust self-renewal capacity and HSC repopulation was noted when *BMI1*, *PBX1* or *MEIS1* were overexpressed within mutant myeloproliferative neoplasms^43,44^. Additionally, Yang et al. observed that the histone lysine acetyltransferase *KAT7* (HBO1) and a related network of genes is necessary for HSC maintenance and self-renewal^28^.

The clinical implications of the observed immunophenotypic and transcriptional heterogeneity within CD34^+^ HSPC subsets mobilized with various regimens remain unclear. This study was not designed to evaluate how differences in the total number of CD34^+^ cells infused, or the number of specific HSPC subsets infused, might impact clinical outcomes. In addition, given that extended immunophenotyping was performed on only a subset of patients mobilized at Washington University in St. Louis (*n* = 37), comparative analyses between cohorts are probably underpowered. Nevertheless, in a post hoc pooled analysis, a significant association was observed between increasing total number of CD34^+^ cells infused and faster TPE. These data suggest that infusion of higher numbers of CD34^+^ cells (≥7 × 10^6^ CD34^+^ cells kg^–1^) may result in faster TPE. This observation is supported by previous publications suggesting that infusion of >6 × 10^6^ CD34^+^ cells kg^–1^ is associated with improved long-term platelet recovery^45^. In addition, we observed that patients infused with the upper quartile (>75th percentile) of GMPs alone had significantly faster TPE (13 versus 19 days). These data suggest that the number of GMPs within the HSPC graft specifically contributed to platelet engraftment kinetics, which may indicate that the CD34^+^ HSPC subset immunophenotypically defined in this study as GMPs (CD45RA^+^/^−^CD123loCD38^+^/^−^CD10^–^) contains a population of megakaryocytic progenitors capable of rapidly reconstituting platelet engraftment. Based on our gating strategy, this observation is consistent with a recent report demonstrating that unipotent megakaryocyte progenitors lie within the CD34^+^ CD38^+^ CD45RA^–^ population^46^.

In conclusion, the upfront use of a single injection of motixafortide added to G-CSF resulted in rapid, robust and reliable mobilization of optimal numbers of CD34^+^ HSPCs in patients with MM undergoing ASCT. Moreover, extended CXCR4i with motixafortide preferentially mobilized increased numbers of immunophenotypically and transcriptionally primitive HSCs. Future studies may consider similar regimens for HSPC-based, gene-edited platforms where the optimal HSPC collection goal is typically much higher (10–15 × 10^6^ CD34^+^ cells kg^–1^) than that of ASCT (5–6 × 10^6^ CD34^+^ cells kg^–1^), given losses of HSPC-viability-associated gene/base editing^47,48^. In addition, regimens mobilizing higher proportions of primitive HSCs may be particularly advantageous for HSPC-based, gene-edited therapies given that the long-term effectiveness of such therapies relies on the successful ability of modified HSPCs to establish stable, long-term engraftment.

## Methods

### GENESIS trial design

All patients treated on the GENESIS trial (Clinicaltrials.gov: NCT03246529) provided written informed consent before enrollment. Institutional Review Board (IRB) approval of the study protocol, investigator brochure, amendments, informed consent and any other documents provided to the subject was performed by the following: Washington University IRB; University of Miami IRB; Mayo Clinic IRB; University of Oregon Oregon Health & Science University IRB; University of Kansas Medical Center IRB; University of Maryland IRB; Western Cooperative Group IRB; The Loyola University Chicago Health Sciences Campus IRB; UCLA IRB; University of Utah IRB; University of Cincinnati IRB; The Weill Cornell Medical College IRB; UT M.D. Anderson Cancer Center IRB; Ethics Committee Catania 1 Azienda Ospedaliero-Universitaria ‘Policlinico-Vittorio Emanuele’ Catania; Ethics Committee South Reggio Calabria Division Grande Ospedale Metropolitano ‘Bianchi-Melacrino- Morelli’; University of Cologne Ethics Committee; University of Debrecen, Clinical Center, Clinic of Internal Medicine, Hematology Scientific Council for Health – Ethical Committee for Clinical Pharmacology, Central Hospital of Southern Pest, National Institute of Hematology and Infectious Diseases Scientific Council for Health – Ethical Committee for Clinical Pharmacology. Please see Reporting Summary for additional details for each IRB. The study was conducted in accordance with the Guideline for Good Clinical Practice ICH E6(R2)—ICH Harmonized Guideline Integrated Addendum to ICH E6 (R1) (International Conference on Harmonization of Technical Requirements for the Registration of Pharmaceuticals for Human Use), Step 5, 14 June 2017; the Declaration of Helsinki: Seoul, 2008; the US Code of Federal Regulations (Title 21, CFR Part 11, 50, 54, 56 and 312) and/or EU Directives; and/or local country regulations and guidelines.

The GENESIS trial included a preplanned, lead-in, single-arm, open-label period previously reported^49,50^, demonstrating that motixafortide + G-CSF was safe, well tolerated and resulted in 82% (9 of 11) of patients with MM mobilizing ≥6 × 10^6^ CD34^+^ cells kg^–1^ within two apheresis days. The preplanned review of these Part 1 data led the independent data monitoring committee (DMC) to recommend transitioning to Part 2 of the study. In the prospective, phase 3, double-blind, placebo-controlled, multicenter Part 2 of the study, 122 patients undergoing ASCT were enrolled at 18 sites across five countries and were randomized 2:1 to receive either motixafortide + G-CSF or placebo + G-CSF for HSPC mobilization before ASCT for MM. Key eligibility criteria included: patients 18–78 years of age with a confirmed diagnosis of MM; an Eastern Cooperative Oncology Group performance status of 0–1; and adequate organ function undergoing first ASCT in first or second complete response (CR) or partial response (PR) (according to IMWG Response Criteria). Sex and/or gender were recorded for demographic purposes, as self-reported by the participant. Patients were excluded if they had undergone prior HCT or failed previous HSPC collection attempts (see full inclusion and exclusion criteria in supplemental GENESIS trial protocol). Randomization of eligible patients was performed using an interactive web response system and was conducted in permuted blocks with stratification by response status (CR versus PR) and baseline platelet count (<200 × 10^9^ l^–1^ or ≥200 × 10^9^ l^–1^). Randomization was performed by the clinical research coordinator, who was not involved in direct patient care. All patients, investigators and providers/staff were blinded to treatment assignment. All patients received G-CSF (10 mcg kg^–1^) on days 1–5 (and 6–8, if needed). Patients received either motixafortide (1.25 mg kg^–1^, subcutaneous injection) or placebo on day 4 (and 6, if needed). Apheresis began on day 5 (four blood volumes), with the primary and secondary endpoints of collecting ≥6 × 10^6^ CD34^+^ cells kg^–1^ in up to two or one apheresis days, respectively. Apheresis continued on days 6–8 if needed. Total CD34^+^ cells kg^–1^ were analyzed locally to determine whether patients met the primary endpoint, and all samples were subsequently sent for assessment by a central laboratory. Patients who did not collect ≥2 × 10^6^ CD34^+^ cells kg^–1^ by day 8 proceeded to rescue mobilization. To assess the impact of each mobilization regimen on PB CD34^+^ cells and the relationship to collection of CD34^+^ HSPCs via apheresis, peripheral blood CD34^+^ HSPC counts were assessed using both local and central laboratories at the following time points: day 0 (baseline), before first dose of G-CSF; day 4, before first dose of motixafortide/placebo but after four doses of G-CSF; and day 5 (apheresis day 1), after first administration of motixafortide or placebo and after five administrations of G-CSF. The number of CD34^+^ cells infused for ASCT was determined independently by each investigator according to local practice; however, a minimum of ≥2 × 10^6^ CD34^+^ cells kg^–1^ was required. Although initial power calculations called for 177 patients to be enrolled in Part 2 of the study, a preplanned interim analysis after 122 patients were enrolled was performed by an independent unblinded statistician and these results were communicated only to the independent DMC for review, leading the DMC to recommend halting the study due to statistically significant efficacy favoring the motixafortide + G-CSF mobilized cohort (prespecified threshold *P* ≤ 0.0108678).

### Correlative study design

All patients who participated in correlative studies provided written informed consent before enrollment on their respective protocols. GENESIS trial patients were enrolled and mobilized as previously described. In addition, a demographically similar, contemporaneous cohort of patients (*n* = 14) undergoing mobilization with plerixafor + G-CSF (regardless of PB CD34^+^ cell count preapheresis) for ASCT for MM were prospectively enrolled on a parallel tissue-banking protocol (no. 201103349). All patients with MM received G-CSF (10 mcg kg^–1^) on days 1–5 (and 6–8, if needed). Patients then received either motixafortide (1.25 mg kg^–1^) or placebo on day 4 (and 6, if needed) via subcutaneous injection, or plerixafor (0.24 mg kg^–1^) on day 4 (and 5–7, if needed) via subcutaneous injection. Apheresis began on day 5 (and 6–8, if needed). Three healthy allo-donor cohorts underwent single-agent mobilization with either (1) G-CSF alone (10 mcg kg^–1^) on days 1–5 followed by apheresis beginning on day 5 (protocol no. 201106261); (2) plerixafor alone (0.24 mg kg^–1^) on day 1 followed by apheresis within 4 h of plerixafor administration (NCT00241358); or (3) motixafortide alone (1.25 mg kg^–1^) on day 1 followed by apheresis within 3 h of motixafortide administration (NCT02639559). Study samples were obtained from the apheresis product on the first day of apheresis in all patients, and CD34^+^ cells were analyzed as detailed below for correlative studies.

### Post hoc engraftment kinetics

Although the number of CD34^+^ cells kg^–1^ infused for ASCT on the GENESIS trial was determined independently by each investigator according to local practice, a minimum of ≥2 × 10^6^ CD34^+^ cells kg^–1^ was required. A post hoc pooled analysis was performed using Pearson correlation to evaluate TPE (platelet count ≥20 × 10^9^ l^–1^ without platelet transfusion ×7 days) and TNE (absolute neutrophil count ≥ 0.5 × 10^9^ l^–1^ ×3 days) based on the total number of CD34^+^ cells kg^–1^ infused without regard to mobilization regimen, as well as the total number of specific CD34^+^ HSPC subsets infused. CD34^+^ HSPC immunophenotyping was performed via multicolor FACS, as detailed below.

### Multicolor FACS

Peripheral blood CD34^+^ HSPC counts were assessed using a Stem Cell Enumeration Kit (BD Biosciences, no. 344563) at both local and central laboratories. For correlative studies, CD34^+^ HSPCs from *n* = 51 patients (placebo + G-CSF, *n* = 13; plerixafor + G-CSF, *n* = 14; motixafortide + G-CSF, *n* = 24) were purified from apheresis product collected on the first day of apheresis via CD34^+^ immunomagnetic selection using an AutoMACS device (Miltenyi Biotech). CD34^+^ HSPCs were washed in PBS and stained for 15 min at room temperature with a LIVE/DEAD Fixable Aqua Dead Cell Stain kit (Invitrogen). Cells were then washed in PBS supplemented with 0.5% bovine serum albumin and 2 mM EDTA and incubated for 10 min at room temperature with human Fc Block and Brilliant Stain Buffer (BD Biosciences). Samples were then incubated for 30 min at room temperature with pretitrated saturating dilutions of the following fluorochrome-labeled antibodies (clone, source designated, catalog no. and dilution in parentheses): CD45-BUV395 (HI30, BD Biosciences, no. 563792, 1:60); CD123-BUV737 (7G3, Biosciences, no. 741769, 1:120); CD49f-BV421 (GoH3, BioLegend, no. 313624, 1:300); CD14-BV650 (M5E2, BioLegend, no. 301836, 1:120); CD45RA-BV785 (HI100, BioLegend, no. 304140, 1:120); CD34-VioBright515 (REA1164, Miltenyi, 1:120); CD10-PECF594 (HI10a, Biosciences, no. 562396, 1:484); CD38-PE-Cy7 (HIT2, BioLegend, no. 303516, 1:120); CD90-APC (5E10, BioLegend, no. 328114, 1:60); CD303-APCVIO770 (REA693, Miltenyi Biotech, no. 130-120-517, 1:150); CD184-PE (1D9, BD, no. 551510, 1:242); and CD184-PE (12G5, Biosciences, no. 555974, 1:150). Fluorescence minus-one controls were used to assess background fluorescence intensity and set gates for negative populations. After washing twice, samples were analyzed on a ZE5 (Bio-Rad) flow cytometer. Single-stain compensation controls were obtained using UltraComp eBeads (Thermo Fisher Scientific) and data were analyzed using FCS Express (DeNovo Software). The antibody-binding capacity per cell of the different CD34^+^ HSPC subsets was determined for CD184 clones 12G5 and 1D9 using saturating concentrations of antibody and the Quantum Simply Cellular (QSC, Bangs Laboratories) system for fluorescence quantitation according to the manufacturers recommendations.

### Single-cell library preparation and sequencing

CD34^+^ HSPCs from *n* = 12 patients with MM (placebo + G-CSF, *n* = 4; plerixafor + G-CSF, *n* = 4; motixafortide + G-CSF, *n* = 4), and from *n* = 6 healthy allogeneic HSPC donors (G-CSF, *n* = 2; plerixafor, *n* = 2; motixafortide, *n* = 2), were purified from apheresis products collected on the first day of apheresis via CD34 immunomagnetic selection. Transcriptional profiling was performed by 10×, 5’ scRNA sequencing. For sample preparation on the 10X Genomics platform, the Chromium Next GEM Single Cell 5’ Kit v.2, 16 rxns (no. PN-1000263), Chromium Next GEM Chip K Single Cell Kit, 48 rxns (no. PN-1000286) and Dual Index Kit TT Set A, 96 rxns (no. PN-1000215) were used. The concentration of each library was accurately determined through quantitative PCR utilizing the KAPA library Quantification Kit according to the manufacturer’s protocol (KAPA Biosystems/Roche), to produce cluster counts appropriate for the Illumina NovaSeq6000 instrument. Normalized libraries were sequenced on a NovaSeq6000 S4 Flow Cell using XP workflow and a 151 × 10 × 10 × 151 sequencing recipe according to the manufacturer’s protocol. A median sequencing depth of 50,000 reads per cell was targeted for each gene expression library.

### scRNA-seq data preprocessing

For each sample we obtained the unfiltered feature-barcode matrix by passing demultiplexed FASTQs to Cell Ranger v.6.0.1 ‘count’ command using default parameters and the prebuilt GRCh38 genome reference (no. GRCh38-2020-A), and Chemistry flag (Single Cell 5’ PE) for scRNA (data files available via the gene expression omnibus (accession no. GSE223972). Seurat 4.1.0 was used for all subsequent analyses. Cells were further filtered to maintain only those cells with <20% human mitochondrial DNA content and a minimum of at least 200 and maximum of 20,000 genes expressed. We constructed a Seurat object using the unfiltered feature-barcode matrix for each sample. Each sample was scaled and normalized using Seurat’s ‘SCTransform’ function to correct for batch effects (with parameters ’vars.to.regress = c(‘nCount_RNA’, ‘percent.mito’)’, variable.features *n* = 2,000). Any merged analysis or subsequent subsetting of cells/samples underwent the same scaling and normalization method. Cells were clustered using the original Louvain algorithm, and the top30 principal component analysis dimensions via functions ‘FindNeighbors’ and ‘FindClusters’ (with parameters: resolution = 0.5). The resulting merged and normalized matrix was used for subsequent analysis.

### scRNA-seq cell-type annotation

Cell types were assigned to each cluster by manually reviewing the expression of a comprehensive set of marker genes derived from several publications^9,26**–**29^.

### scRNA-seq Monocle trajectory analysis

Monocle3 (https://cole-trapnell-lab.github.io/monocle3/) was used for pseudotime analysis. Analysis was completed following the standard tutorial for construction of single-cell trajectories (https://cole-trapnell-lab.github.io/monocle3/docs/trajectories/).

### scRNA-seq DEG analysis

For cluster-level differential expression we used the ‘FindMarkers’ or ‘FindAllMarkers’ Seurat function as appropriate, with a minimum percentage of 0.3 (parameter min.pct = 0.3). The resulting DEGs were then filtered for adjusted *P* < 0.05 and sorted by FC. All differential expression analyses were carried out using the ‘SCT’ assay.

### Gene expression scores

Gene expression scores for each cell were annotated using the AddModuleScore function in Seurat. Gene sets were extracted from www.gsea-mdsigdb.org. The TNFA_NFKB_Score gene set was derived from the HALLMARK_TNFA_SIGNALING_VIA_NFKB pathway. EGF_Score was derived from NAGASHIMA_EGF_SIGNALING_UP and MIT_EGF_RESPONSE_40_HELA. HBO1_score was derived from Yang et al^28^, including the following genes: *KAT7*, *MPL*, *TEK*, *GFI1B*, *EGR1*, *TAL1*, *GATA2*, *ERG*, *PBX1, MEIS1*, *HOXA9* and *GATA1*. For all gene sets, the percentage of cells expressing each gene in the set was calculated for each treatment group. Any genes with <10% expression across a given treatment group were filtered out and not used in the final gene score annotation.

### Healthcare resource utilization

As a prespecified analysis, healthcare resource utilization items were collected alongside the GENESIS trial in each treatment arm, including (1) the number of motixafortide and G-CSF doses, (2) the number of apheresis procedures used in primary mobilization and (3) the proportion of patients requiring rescue mobilization due to inadequate primary mobilization.

### Statistics

The primary endpoint was the proportion of patients mobilizing ≥6 × 10^6^ CD34^+^ cells kg^–1^ within up to two apheresis sessions in preparation for auto-HCT following G-CSF and a single administration of BL-8040/placebo. Prespecified secondary endpoints included the proportion of patients mobilizing ≥6 × 10^6^ CD34+ cells kg^–1^ in one apheresis, the proportion of patients mobilizing ≥2 × 10^6^ CD34^+^ cells kg^–1^ in one apheresis, TPE, TNE, graft durability, OS and PFS. The primary endpoint, prespecified secondary efficacy endpoints and graft durability were analyzed using the Cochran–Mantel–Haenszel (CMH) test with calculation of OR, two-sided 95% CI and *P* values. Time to engraftment, PFS and OS secondary endpoints were analyzed using Cox’s proportional hazards model, and time-to-event analyses (Kaplan–Meier method) with calculation of HR, two-sided 95% CI and *P* values. Analyses of all prespecified primary and secondary endpoints were performed on an ITT basis unless otherwise stated. *P* < 0.05 was considered statistically significant unless otherwise stated. Pearson correlation was performed to assess associations between total CD34^+^ cells kg^–1^ infused and specific CD34^+^ subset cells kg^–1^ infused and TPE/TNE. Sensitivity analyses were performed using time-to-event analyses (Kaplan–Meier method) to determine thresholds for both total CD34^+^ HSPCs infused and specific HSPC subsets infused above which TPE was significantly faster. Mean HSPC subset yields in apheresis were compared using analysis of variance, followed by post hoc Tukey–Kramer test for pairwise comparisons among groups. Two-sided statistical analyses were used.

### Reporting summary

Further information on research design is available in the Nature Portfolio Reporting Summary linked to this article.

## Online content

Any methods, additional references, Nature Portfolio reporting summaries, source data, extended data, supplementary information, acknowledgements, peer review information; details of author contributions and competing interests; and statements of data and code availability are available at 10.1038/s41591-023-02273-z.

### Supplementary information

Supplementary Information Clinical trial protocol.

Reporting Summary

Supplementary Table 1 Antibodies for correlative CD34^+^ HSPC immunophenotyping. BDB, BD Biosciences; BL, BioLegend; MB, Miltenyi Biotech; PBMCs, peripheral blood mononuclear cells; hPBMCs, human PBMCs; mPBMCs, murine PBMCs; hALL, human acute lymphoblastic leukemia; hAML, human acute myeloid leukemia.

## Figures and Tables

**Extended Data Table 1:**
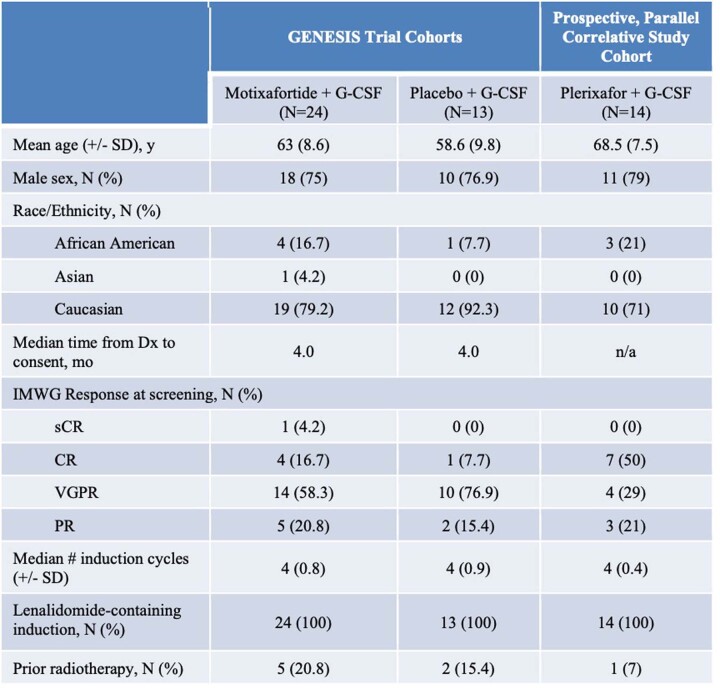
Correlative Study Demographics for GENESIS Trial and Contemporaneous MM Cohort

Among MM patients whose CD34+ HSPCs underwent additional correlative immunophenotypic and transcriptional profiling, similar demographics were observed between those mobilized on the GENESIS Trial with motixafortide+G-CSF (N = 24) or placebo+G-CSF (N = 13), and those mobilized with plerixafor+G-CSF (N = 14) on the prospectively enrolled, parallel correlative study.

**Extended Data Table 2:**
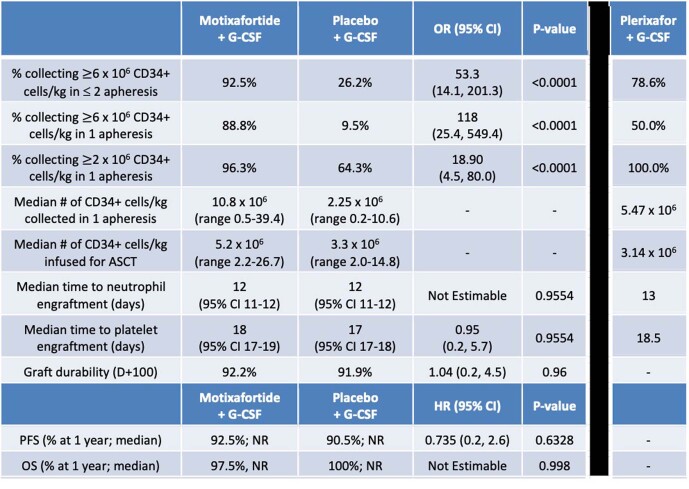
GENESIS Trial Primary and Secondary Endpoints

Primary and secondary endpoints for the GENESIS Trial stratified by mobilization regimen. The proportion of patients collecting to the stated CD34+ cell/kg targets are presented with odds ratio (OR), 2-sided 95% confidence intervals (CI) and p-values based on CMH analysis on an intent-to-treat basis. Median numbers of CD34+ cells/kg collected and infused are presented as median values with range (minimum, maximum); but without comparative statistics as this was not a pre-specified analysis. Time-to-engraftment was assessed by Kaplan-Meier analysis and is presented as the median time to event with 2-sided 95% CI and p-values. Graft durability was assessed by CMH, as previously described, and presented with OR, 2-sided 95% CI and p-value. Progression free survival (PFS) and overall survival (OS) was assessed using Cox’s proportional hazard’s model and is presented as proportion not meeting event at 1-year with hazard ratio (HR), two-sided CI and p-values. A hierarchical method for multiple endpoint testing was utilized to control for multiple comparisons. The final column represents the apheresis and clinical outcomes observed in the contemporaneous MM cohort mobilized with plerixafor+G-CSF on a separate, prospective protocol conducted in parallel to the GENESIS Trial. Statistical comparisons between primary/secondary endpoints for patients mobilized on the GENESIS Trial and those mobilized with plerixafor+G-CSF were not pre-planned; nor performed.

## Data Availability

All data and/or supporting documents related to the paper that Nature reviewers and/or editors will request for the purposes of evaluating this paper and verifying its contents will be provided through Egnyte system, at any time. Data will also be available to researchers and/or scientists in alignment with the International Committee of Medical Journal Editors’ policy on clinical data sharing. Specifically, the authors will provide access to individual deidentified participant-level data that underlie the data presented in this paper, including data dictionaries, the study protocol and other relevant information, to any researcher who provides a methodologically sound proposal for academic purposes beginning 6 months and ending 5 years after article publication. These data can be requested via email to iritg@biolinerx.com, and will be made available to requesting parties through the Egnyte system following approval. This request and availability mechanism for accessing the clinical dataset will similarly apply to requests for the ‘minimum dataset’ necessary to interpret, verify and extend the research in the article. All requests for clinical data will be reviewed by the sponsor (BioLineRx) to verify whether the request is subject to any intellectual property or confidentiality obligations. The gene count matrices for scRNA-seq are available via Gene Expression Omnibus (accession no. GSE223972). References (GRCh38 genome reference, namely refdata-gex-GRCh38-2020-A) used for single-cell analysis of human genomes are available from public sources: https://support.10xgenomics.com/single-cell-gene-expression/software/release-notes/build.

## Code availability

Code relevant for single-cell analysis can be found on GitHub (https://github.com/reykajayasinghe/WashU_Genesis_Study). Additionally, software and code used for the analyses presented in this study are enumerated within the Reporting Summary section on software and code and detailed here: Commercially available R, SPSS, SAS and Xcel statistical and graphics software packages were used for data analysis and graphics; Monocle3 (https://cole-trapnell-lab.github.io/monocle3/) was used for pseudotime analysis. Analysis was completed following the standard tutorial for construction of single-cell trajectories (https://cole-trapnell-lab.github.io/monocle3/docs/trajectories/); Bioconda, https://bioconda.github.io/; Bioconductor v.3.9, https://bioconductor.org/; Cell Ranger v.6.0.1, 10X Genomics, https://support.10xgenomics.com/single-cell-gene-expression/software/pipelines/latest/what-is-cell-ranger; data.table_1.12.6, R Development Core Team, https://cran.r-project.org/package=data.table; dendsort_0.3.3, https://cran.r-project.org/package=dendsort; dplyr_0.8.5, R Development Core Team, https://cran.r-project.org/package=dplyr; ggplot2_3.3.2, R Development Core Team, https://CRAN.R-project.org/package=ggplot2; gridExtra_2.3, R Development Core Team, https://cran.r-project.org/package=gridExtra; magrittr_1.5, R Development Core Team, https://cran.r-project.org/package=magrittr; Matrix_1.2-17, R Development Core Team, https://CRAN.R-project.org/package=Matrix; pheatmap_1.0.12, R Development Core Team, https://cran.r-project.org/package=pheatmap; Python v3.7, Python Software Foundation, https://www.python.org/; R v.3.6, R Development Core Team, https://www.r-project.org/; RColorBrewer_1.1-2, R Development Core Team, https://CRAN.R-project.org/package=RColorBrewer; reshape2_1.4.3, R Development Core Team, https://cran.r-project.org/package=reshape2; SAS v.9.4 for statistical analyses; Seurat v.3.1.2 and v.4.0.3, https://cran.r-project.org/web/packages/Seurat; Single-cell analysis code (https://github.com/reykajayasinghe/WashU_Genesis_Study); sva v.3.40.0, https://bioconductor.org/packages/release/bioc/html/sva.html; stringr_1.4.0, R Development Core Team, https://cran.r-project.org/package=stringr; Subread v.2.0.1, https://sourceforge.net/projects/subread/; Tidyverse, https://www.tidyverse.org/; viridis_0.5.1, R Development Core Team, https://github.com/sjmgarnier/viridis; viridisLite_0.3.0, R Development Core Team, https://github.com/sjmgarnier/viridis.
