# Supplementary material for: Motixafortide and G-CSF to mobilize hematopoietic stem cells for autologous transplantation in multiple myeloma: a randomized phase 3 trial
Source: Nat Med. 2023 Apr 17;29(4):869–79. doi: 10.1038/s41591-023-02273-z (PMC10115633; doi:10.1038/s41591-023-02273-z)
Supplement: Supplementary file 1 — Clinical trial protocol. [file 41591_2023_2273_MOESM1_ESM.pdf]

nature medicine

Article https://doi.org/10.1038/s41591-023-02273-z

Motixafortide and G-CSF to mobilize hematopoietic stem cells for autologous transplantation in multiple myeloma: a randomized phase 3 trial

In the format provided by the authors and unedited

**Investigational Product:** BL-8040	Abbreviated Title: BL-8040 with G-CSF for Stem Cell Mobilization in Multiple Myeloma	**Protocol No.** BL-8040.SCM.301
Phase: III		Version 6.1<br>21 April 2020

CLINICAL STUDY PROTOCOL

A PHASE III, RANDOMIZED, DOUBLE-BLINDED, PLACEBO-CONTROLLED, MULTI-CENTRE STUDY EVALUATING THE SAFETY, TOLERABILITY AND EFFICACY OF COMBINATION TREATMENT OF BL-8040 AND G-CSF AS COMPARED TO PLACEBO AND G-CSF FOR THE MOBILIZATION OF HEMATOPOIETIC STEM CELLS FOR AUTOLOGOUS TRANSPLANTATION IN SUBJECTS WITH MULTIPLE MYELOMA – THE GENESIS STUDY

Protocol No.	BL-8040.SCM.301
Study Phase:	III
Sponsors:	BioLineRx, Ltd.<br>Modi'in Technology Park<br>2 HaMa'ayan Street<br>Modi'in 7177871, Israel<br>Tel: +972-8-642-9100; Fax: +972-8-642-9101
IND No.	113776
EudraCT No.	2018-001715-79
Principal Investigator(s):	**John F DiPersio, MD, PhD**<br>Virginia E and Samuel J Golman Professor<br>Chief, Division of Oncology<br>Deputy Director, Siteman Cancer Center<br>Washington University School of Medicine<br><br>**Zachary D. Crees, MD**<br>Department of Medicine<br>Washington University School of Medicine
VP Research and Development:	Ella Sorani, PhD
VP Clinical Development:	Abi Vainstein-Haras, MD
Protocol Version and Date:	Version 6.1, 21 April 2020

Confidentiality Statement:

This protocol is a confidential communication document of BioLineRx, Ltd. The recipient of this document agrees not to disclose the information contained herein to others without prior written authorization of BioLineRx, Ltd. except that this document may be disclosed to appropriate Institutional Review Boards/Independent Ethics Committees or duly authorized representatives of Regulatory authorities, under the condition that they maintain confidentiality.

PROTOCOL SIGNATURE PAGE

Protocol Title	A Phase III, Randomized, Double-Blinded, Placebo-Controlled, Multi-Centre Study Evaluating the Safety, Tolerability and Efficacy of Combination Treatment of BL-8040 and G-CSF as compared to Placebo and G-CSF for thE MobilizatioN of HematopoiEtic Stem Cells for Autologous TransplantatIon in SubjectS with Multiple Myeloma – **The GENESIS Study**
Protocol Identification	BL-8040.SCM.301, version 6.1 (dated 21 April 2020)
Study Phase	III
Sponsor	BioLineRx Ltd., Israel

Sponsor Representatives

We, the undersigned, have read this protocol and agree that it contains all necessary information required to conduct the trial and that the protocol is in compliance with International Conference on Harmonization (ICH) and Good Clinical Practice (GCP) guidelines and applicable local regulations.

Abi Vainstein-Haras, MD
VP Clinical Development

[signature] April 21st 2020

Signature Date

Ella Sorani, PhD
VP Research and Development

[signature] 21/April/2020

Signature Date

Principal Investigator

By signing below, I, the Investigator, approve the protocol and agree to conduct the clinical trial according to all stipulations of the protocol as specified in both the clinical and administrative sections, CRF and any protocol-related documents (subject to any amendments agreed to in writing between the Sponsor and Principal Investigator). I agree to comply with the ICH-GCP, World Medical Association Declaration of Helsinki (and relevant updates) and applicable local regulations. I agree to ensure that the confidential information contained in this document will not be used for any purpose other than the evaluation or conduct of the clinical investigation without the prior written consent of BioLineRx Ltd. I understand that the study may be terminated or enrollment suspended at any time by sponsor, or by me, at my center, if it becomes necessary in my opinion, to protect the best interests of the study subjects.

Name Investigator Signature Date

Center's Name City, Country

TABLE OF CONTENTS

TABLE OF CONTENTS 4
LIST OF TABLES 7
TABLE OF FIGURES 7
LIST OF APPENDICES 8
PROTOCOL SYNOPSIS 9
GLOSSARY 39
1. INTRODUCTION 42
1.1 THERAPEUTIC INDICATION 42
1.2 INVESTIGATIONAL THERAPY 42
1.2.1 BL-8040 42
1.2.2 G-CSF (Filgrastim) 44
1.2.3 Transplantation 45
1.3 STUDY RATIONALE 46
2. STUDY OBJECTIVES AND ENDPOINTS 47
2.1 STUDY OBJECTIVES 47
2.1.1 Primary Objective 47
2.1.2 Secondary Objectives 47
2.1.3 Exploratory Objectives 47
2.1.4 Safety and Tolerability Objectives 48
2.1.5 Pharmacokinetic Endpoint 48
2.1.6 Pharmacoeconomic Endpoints 48
2.2 STUDY ENDPOINTS/OUTCOMES 48
2.2.1 Efficacy Endpoints 48
2.2.2 Exploratory Biomarkers 49
2.2.3 Safety and Tolerability Outcomes 49
2.2.4 Pharmacokinetic Endpoint 49
2.2.5 Pharmacoeconomic Endpoints 49
3. STUDY DESIGN 49
3.1 PART 1 49
3.2 PART 2 50
3.3 BOTH PARTS 50
4. STUDY POPULATION 52
4.1 NUMBER OF SUBJECTS 52
4.2 INCLUSION CRITERIA 52
4.3 EXCLUSION CRITERIA 54
4.4 SUBJECT IDENTIFICATION 56
4.5 SCREENING FAILURES 56
4.6 REMOVAL, EARLY WITHDRAWAL OF SUBJECTS FROM THERAPY OR ASSESSMENT 56
4.7 HANDLING OF WITHDRAWALS 56
4.8 SPONSOR'S TERMINATION OF STUDY 57
5. STUDY PROCEDURES AND ASSESSMENTS 57

5.1 SCREENING PERIOD (FROM THE TIME OF INFORMED CONSENT FORM (ICF) SIGNATURE UNTIL DAY 0 (BASELINE VISIT)) 57
5.2 TREATMENT PERIOD 59
5.2.1 Baseline Visit (Visit Day 0) 59
5.2.2 Randomization (Part 2 only) 59
5.2.3 Schedule of Treatment Period (Day 1 to Day 8) 60
5.2.4 Apheresis procedure 67
5.2.5 Rescue Procedure 67
5.2.6 Post Treatment Period 67
5.2.7 Visit 11-13: Graft Durability and Core Study Termination Visit 69
5.2.8 Long Term Disease Assessment Follow-Up Post Transplantation 72
5.3 SAFETY ASSESSMENTS 72
5.3.1 Adverse Events (AEs) 72
5.3.2 Concomitant Medications 73
5.3.3 Vital Signs 73
5.3.4 Electrocardiogram 74
5.3.5 During Parts 1 and 2 75
5.3.6 Laboratory Assessments 76
5.3.7 Assessment of Anti-Drug Antibodies (ADA), Complement Activation and Mast Cell Activation 78
5.4 EFFICACY ASSESSMENTS 78
5.4.1 CD34+ Cell Enumeration in the Apheresis Product 78
5.4.2 CD34+ Cell Enumeration in Peripheral Blood (PB) 78
5.4.3 Assessment of Engraftment by CBC 79
5.5 DISEASE STATUS ASSESSMENT BY BONE MARROW BIOPSIES 79
5.6 PK ASSESSMENTS 79
5.7 BLOOD SAMPLING AND PROCESSING 79
5.8 PHARMACOECONOMIC ASSESSMENTS 80
6. INVESTIGATIONAL PRODUCT 80
6.1 IDENTITY OF INVESTIGATIONAL PRODUCT 80
6.2 STUDY DRUG ADMINISTRATION AND DOSAGE 80
6.2.1 BL-8040 or Placebo 80
6.2.2 G-CSF 82
6.3 MANUFACTURING OF STUDY MEDICATION 83
6.3.1 BL-8040 83
6.3.2 G-CSF 83
6.4 PACKAGING AND LABELING OF STUDY MEDICATION 83
6.5 DISTRIBUTION AND SHIPMENT OF STUDY MEDICATION 83
6.6 STORAGE, DISPENSING AND RETURN OF THE INVESTIGATIONAL MEDICINAL PRODUCT 84
6.6.1 BL-8040 or Placebo 84
6.6.2 G-CSF (Filgrastim) 84
6.7 ACCOUNTABILITY AND COMPLIANCE OF INVESTIGATIONAL MEDICINAL PRODUCT 84

6.8 CONCOMITANT THERAPY 85
6.8.1 Acceptable Concomitant Medications 85
6.8.2 Prohibited Concomitant Medications 86
7. SAFETY AND PHARMACOVIGILANCE 87
7.1 ADVERSE EVENT (AE) 87
7.2 SERIOUS ADVERSE EVENTS (SAEs) 89
7.3 DEFINITION OF AN UNEXPECTED ADVERSE EVENT 90
7.4 EXCEPTIONS IN THE REPORTING OF SAE 90
7.5 NOTIFICATION OF SERIOUS ADVERSE EVENT (SAE) 91
7.6 TIMEFRAME FOR REPORTING REQUIRED EVENTS 92
8. STATISTICAL METHODOLOGY 93
8.1 GENERAL 93
8.2 RANDOMIZATION 93
8.3 INTERIM ANALYSIS, TYPE-I ERROR AND MULTIPLICITY ADJUSTMENT 93
8.4 SAMPLE SIZE JUSTIFICATION 94
8.4.1 Sample Size Justification for Part 1 (Open labeled period for ascertaining the BL-8040 dose optimization) 94
8.4.2 Sample Size Justification for Part 2 (Randomized, Placebo-Controlled Part) 94
8.5 DATA ANALYSES SETS 95
8.6 SEQUENCE OF PLANNED ANALYSES 95
8.7 INTERIM ANALYSES 97
8.8 EFFICACY ENDPOINTS AND ANALYSES 98
8.8.1 Primary Efficacy Endpoint and Principal Statistical Analysis 98
8.8.2 Secondary Efficacy Endpoints and Statistical Analysis 99
8.8.3 Additional Secondary Efficacy Endpoints and Between Study Groups Descriptive Comparability Assessment 100
8.8.4 Exploratory Endpoints 101
8.9 SAFETY AND TOLERABILITY ANALYSES 102
8.9.1 Adverse Events 102
8.9.2 Safety Laboratory Data 102
8.9.3 Vital Signs 103
8.9.4 Physical Examinations 103
8.9.5 12-Lead ECG 103
8.9.6 Tolerability Assessment 104
8.10 PHARMACOKINETIC ASSESSMENTS 104
8.11 MORE DETAILED STATISTICAL ANALYSIS PLAN (SAP) 104
8.12 STATISTICAL SOFTWARE 104
9. ETHICS 105
9.1 INSTITUTIONAL REVIEW BOARD (IRB) OR INDEPENDENT ETHICS COMMITTEE (IEC) 105
9.2 ETHICAL CONDUCT OF THE STUDY 105
9.3 SUBJECT INFORMATION AND CONSENT 105
9.4 SUBJECT INSURANCE 106
9.5 INFORMING THE GENERAL PRACTITIONER 106

9.6 PERSONAL DATA PROTECTION 106
9.7 DATA AND SAFETY MONITORING 106
9.8 PROTOCOL EXCEPTIONS AND DEVIATIONS 106
9.9 PROTOCOL AMENDMENTS 106
10. QUALITY CONTROL AND QUALITY ASSURANCE 107
10.1 AUDITS AND INSPECTIONS 107
10.2 STUDY MONITORING 107
10.3 QUALITY LABORATORY STANDARDS 108
10.4 STUDY DOCUMENTATION 108
10.4.1 Source Document 109
10.4.2 Recording of Data on Case Report Form (CRF) 109
10.4.3 Investigator Site File 110
10.5 CLINICAL TRIAL SUPPLIES 110
10.6 DATA MANAGEMENT 110
11. STUDY ADMINISTRATION 111
11.1 REQUIRED DOCUMENTS PRIOR TO STUDY INITIATION 111
11.2 STUDY COMPLETION 111
11.3 CLINICAL STUDY REPORT (CSR) 112
11.4 RETENTION OF STUDY RECORDS 112
11.5 CONFIDENTIALITY AND PUBLICATION OF STUDY RESULTS 113
11.5.1 General 113
11.5.2 Published Data 114
12. REFERENCES 115
13. APPENDICES 117

LIST OF TABLES

Table 1: Laboratory Assessments 77
Table 2: Injected Volume based on Patient's Weight 82
Table 3: Severity of Adverse Events According to CTCAE (Version 5) 88
Table 4: Relationship of Adverse Event to Treatment 89
Table 5: BL-8040-SCM.301 (GENESIS) Schedule of Assessments – Treatment Period 117
Table 6: BL-8040-SCM.301 (GENESIS) Schedule of Assessments – Post Treatment Period 121

TABLE OF FIGURES

Figure 1: Stem Cell Mobilization Procedure 52

LIST OF APPENDICES

Appendix A: Schedule of Assessments 117
Appendix B: Eastern Cooperative Oncology Group Performance Status 123
Appendix C: International Myeloma Working Group (IMWG) Uniform Response Criteria 124
Appendix D: CIBMTR (Engraftment Guideline) 125

PROTOCOL SYNOPSIS

Study Title	**A Phase III, Randomized, Double-Blinded, Placebo-Controlled, Multi-Centre Study Evaluating the Safety, Tolerability and Efficacy of Combination Treatment of BL-8040 and G-CSF as compared to Placebo and G-CSF for the Mobilization of Hematopoietic Stem Cells for Autologous Transplantation in Subjects with Multiple Myeloma – The GENESIS Study**
Protocol No.	BL-8040.SCM.301
EudraCT:	2018-001715-79
Clinical Sites	Approximately 35 sites in the USA, Canada and Europe
Study Phase	III
Therapeutic Indication	Hematopoietic Stem Cell Mobilization for Autologous Transplantation in Subjects with Advanced Hematologic Malignancies (Multiple Myeloma (MM).
Study Objectives	**Primary Objective:**<br>To demonstrate the superiority of one dose of BL-8040 + G-CSF over placebo + G-CSF to mobilize ≥6.0 x 10^6 CD34+ cells/kg in up to 2 apheresis sessions in preparation for autologous hematopoietic cell transplantation (auto-HCT) in MM subjects.<br>**Secondary Objectives:**<br>• To demonstrate the superiority of one dose of BL-8040 + G-CSF over placebo + G-CSF to mobilize ≥2.0 x 10^6 CD34+ cells/kg in 1 apheresis session in preparation for autologous hematopoietic cell transplantation (auto-HCT) in MM subjects.<br>• To demonstrate the superiority of one dose of BL-8040 + G-CSF over placebo + G-CSF to mobilize ≥6.0 x 10^6 CD34+ cells/kg in 1 apheresis session in preparation for autologous hematopoietic cell transplantation (auto-HCT) in MM subjects.<br>• To descriptively assess the comparability between the effects of BL-8040 + G-CSF and placebo + G-CSF in time to neutrophil engraftment, platelet engraftment and the later of the two.<br>• To descriptively assess the comparability between the effects of BL-8040 + G-CSF and placebo + G-CSF on graft durability at 60 days, 100 days, and 6 and 12-months post-transplantation.<br>• To demonstrate that the use of (one or two doses) BL-8040 + G-CSF over placebo + G-CSF results in a reduction in resource use and cost savings.<br>**Exploratory Objectives:**<br>• To demonstrate the superiority of BL-8040 + G-CSF over placebo + G-CSF in increasing CD34+ cell concentration in the peripheral blood on apheresis Day 1 or Day 1 and 2, if applicable.<br>• To descriptively assess the comparability between the effects of BL-8040 + G-CSF and placebo + G-CSF in graft failure post-transplantation.<br>• To descriptively assess the comparability between the effects of BL-8040 + G-CSF and placebo + G-CSF in overall survival with up to 5 years follow-up.

Study Objectives	• To descriptively assess the comparability between the effects of BL-8040 + G-CSF and placebo + G-CSF in relapse-free survival with up to 5 years follow-up.<br>• To descriptively assess the comparability between the effects of BL-8040 + G-CSF and placebo + G-CSF in the mobilization of Multiple Myeloma cells in the apheresis product.<br>• To descriptively compare the CD34+ cells collected per kg of actual body weight (ABW) used in this study to the theoretical amount of CD34+ cells collected based on ideal body weight (IBW)<br>**Safety and Tolerability Objectives:**<br>To demonstrate that the combination of BL-8040 + G-CSF is safe and tolerable as compared to Placebo + G-CSF.<br><br>**Pharmacokinetic Objectives:**<br>• BL-8040 exposure as measured by C_{max} and AUC (See Section 5.6).<br>• Effect of covariates such as body weight and age on BL-8040 exposure.<br>• Exposure-response analysis, if warranted.<br><br>**Pharmacoeconomic Objectives:**<br>The resource utilization up until engraftment, 100 days after transplantation, multiplied with per country unit costs to obtain an average cost per patient per treatment arm.
Study Design	A randomized, double-blinded, placebo-controlled, multi-center, 2-part study to evaluate the effect of BL-8040 in combination with G-CSF on the collection of CD34+ cells for auto-HCT.<br>This Phase III study will be composed of two sequential parts:<br>**Part 1:**<br>This lead-in period is designed to ascertain the dose of BL-8040 and will enroll a total of up to 30 subjects to an open labeled treatment to assess the efficacy, safety and tolerability of treatment with G-CSF 10 µg/kg/day and BL-8040 1.25 mg/kg, per study protocol, to achieve the goal collection of $\geq 6 \times 10^6$ CD34+ cells/kg.<br>An independent data monitoring committee (DMC) will then assess the accumulated data of this lead-in period and recommend whether to continue to Part 2 of the study with BL-8040 1.25 mg/kg. The review of the accumulated data of Part 1 will be conducted in 3 sequential steps:<br>• Initially, the accumulated data of 10 subjects will be summarized and reviewed by the DMC, which may recommend that Sponsor either study an additional cohort of 10 subjects, lower the dose, discontinue the study or continue to Part 2 of the study.<br>• Similarly, after reviewing the accumulated data of a total of 20 subjects, the DMC may recommend that the Sponsor either study an additional cohort of 10 subjects, lower the dose, discontinue the study or continue to Part 2 of the study.<br>• After reviewing the accumulated data of a total of 30 subjects, the DMC will make recommendations regarding study continuation.

Study Design	The following decision rules will be taken into consideration by the DMC:<br>• The primary efficacy endpoint of the study is the proportion (%) of subjects mobilizing ≥6.0 x 106 CD34+ cells/kg with up to two apheresis sessions in preparation for auto-HCT after G-CSF + single administration of BL-8040 or Placebo + G-CSF.<br>• After the review of the accumulated data of 10 subjects, enroll an additional 10 subjects if the number of subjects meeting the primary efficacy endpoint is less than or equal to 6.<br>• After the review of the accumulated data of 20 subjects, enroll an additional 10 subjects if the number of subjects meeting the primary efficacy endpoint is less than or equal to 12.<br>• After the review of the accumulated data of 30 subjects, a decision of early termination of the study should be taken if the number of subjects meeting the primary endpoint is less than or equal to 16.<br><br>Furthermore, the DMC will have the freedom to employ more conservative decision rules based on integrative risk and benefit assessments of the accumulated information of the study at each decision point.<br>All subjects treated during Part 1 will continue to be followed concomitantly to the conduct of Part 2 and according to the same visit schedule until study termination.<br><br>**Part 2:**<br>Following the successful completion of Part 1, a total of 177 subjects will be randomized into Part 2 of the study, which will employ a double-blinded, placebo-controlled design to assess the efficacy, safety and tolerability of BL-8040 + G-CSF as compared to placebo + G-CSF.<br>Subjects to be enrolled during Part 2 of the study will be randomized in a 2:1 ratio to receive either BL-8040 or placebo, respectively, plus G-CSF. Randomization will be stratified based on remission status (Complete Remission (CR) vs. Partial Remission (PR))[a] and baseline platelet count (<200 x 10^9/L or ≥ 200 x 10^9/L). The apheresis product will be processed and stored according to local practice guidelines at each study center.<br>Subjects will undergo mobilization with G-CSF ~10 µg/kg (and maximum of 15 µg/kg) subcutaneously (SC) daily in the morning (8:00AM ± 2 hours) for up to 8 days. Beginning on the evening of Day 4 (8:00PM ± 2 hours), subjects will receive either a single injection of BL-8040 or placebo SC. In the morning of Day 5, a 5th dose of G-CSF will be administered within 1hr prior to apheresis (12 hours ± 2 hours from BL-8040/placebo injection). Subjects will then undergo first apheresis per institutional protocol (4 blood volumes ±10% /apheresis). In the event that the subject does not reach the collection goal for mobilization (≥6.0 x 10^6 CD34+ cells/kg), on Day 6, a 6th dose of G-CSF will be administered within 1 hr prior to the second apheresis session (36 hours ± 2 hours from the BL-8040/placebo injection) in an effort to reach a total of ≥6 x 10^6 CD34+ cells/kg.

[a] Response criteria for the purposes of study stratification will be defined in accordance with the International Myeloma Working Group (IMWG) Uniform Response Criteria see Appendix C. Additionally, we will combine Stringent CR and CR into one "CR" group and VGPR and PR into one "PR" group. Partial Response (PR) including PR and Very Good PR (VGPR) and Complete Response (CR) which includes CR and Stringent CR (SCR)

Study Design	If the subject does not reach the collection goal for mobilization (≥6.0 x 10^6 CD34+ cells/kg), a second dose of BL-8040/placebo will be administered in the evening of Day 6 (48 hours ± 2 hours from the initial BL-8040/placebo injection). In the morning of Day 7, a 7th dose of G-CSF will be administered within 1 hr prior to the third apheresis session (12 hours ± 2 hours from the second BL-8040/placebo injection). If the subject still does not reach the collection goal for mobilization (≥6.0 x 10^6 CD34+ cells/kg), a fourth and final apheresis will be performed on Day 8 (36 hours ± 2 hours from the second BL-8040/placebo injection), with an 8th dose of G-CSF administered within 1 hour prior to the apheresis, in an effort to collect a total of ≥6 x 10^6 CD34+ cells/kg, but at least ≥ 2 x 10^6 CD34+ cells/kg from the combined collections. Subjects who fail to collect the minimum required CD34+ cells/kg will be subsequently managed per investigator preference in accordance with standard of care guidelines to receive rescue medication. They may reattempt mobilization per standard of care after at least 7 day's "rest/wash-out" period (7 days from the last dose of BL-8040/placebo). Subjects who fail to collect enough CD34+ cells in four apheresis sessions will continue to be followed according to the protocol schedule of visits.<br><br>One interim analysis for potential early termination of new subject recruitment into the study will be conducted at the time when a total of 65% of the originally planned sample size will be randomized and have completed the stem cell mobilization procedure.
Study Procedures	**<u>Visit Procedures</u>**<br><br><u>Screening (Day -28 to Day 0)</u><br><br>From the time of Informed Consent Form (ICF) signature until Day 0 (Baseline Visit).<br><br>During Screening, the subject must be thoroughly informed about all aspects of the study, including scheduled study visits and activities, and must sign the informed consent form (ICF) prior to any study activity.<br><br>Prior to any study activities/evaluation, the subject will be allocated a subject number by the Investigator using an Interactive Web Response System (IWRS), which will be used throughout the study, and assessed for eligibility criteria.<br><br>A window of up to 28 days is allowed for the screening period procedures. Urinary and blood laboratory assessments, as well as ECG, must be completed within 10 days (+2 days) before G-CSF administration, except blood serology, UPEP, SPEP, immunofixation and FLC. Potential subjects will be assessed for fulfilment of the entry requirements as detailed in the Inclusion and Exclusion criteria sections of the synopsis.<br><br>The following procedures will be performed:<br>• ICF process<br>• Inclusion/exclusion criteria review<br>• Collect demographics and medical history<br>• Review prior and concomitant medications<br>• Complete physical examination<br>• IWRS<br>• Adverse Events (AEs) recording starting from ICF signature

Study Procedures	• 12 lead electrocardiogram (ECG)<br>• Vital signs (blood pressure, heart rate, respiratory rate, temperature [°C] and room air SPO2)<br>• Eastern Cooperative Oncology Group (ECOG) performance status<br>• Coagulation Test: Prothrombin Time (PT)/International Normalized Ratio (INR) and Activated Partial Thromboplastin Time (aPTT)<br>• Complete Blood Count (CBC) with differential<br>• Blood chemistry<br>• Serum pregnancy test for all female patients of childbearing potential<br>• Blood Serology Assessment<br>• Monoclonal Protein (M) in serum (SPEP) and serum immunofixation<br>• Monoclonal Protein (M) in urine (UPEP) and urine immunofixation-24 hours urine collection is required<br>• Serum Free Light Chain (FLC)<br>• Urinalysis<br>• Bone marrow biopsy – only for CR/sCR patients<br><br>Subjects who fail the screening procedures may be re-screened. Re-screening should include all screening procedures listed in the protocol schedule of events, including re-consent signature.<br><br>*Of note: Screening Visit procedures can be performed on the same calendar day as Baseline Visit provided those are performed within 3 days before G-CSF administration.*<br><br><u>Baseline Visit, Day 0 (-3 days to Day 0)</u><br><br>Baseline visit should occur within 28 days after the screening visit and within 3 days prior to G-CSF administration. Only subjects who fully comply with the inclusion/exclusion criteria for the study will be eligible for participation.<br><br>*<u>Of note</u>: Baseline visit Day 0 and Visit Day 1 can be done on the same calendar day providing activities related to Baseline are done before G-CSF administration.*<br><br>The following procedures will be performed at the Baseline Visit. A window of three days (-3 days to Day 0) is allowed for the procedures and assessments of this visit:<br><br>• AE recording<br>• Inclusion/Exclusion criteria review<br>• Concomitant Medications review<br>• Directed Physical Examination (if Baseline Visit Day 0 and Visit Day 1 are performed on the same day, Directed PE will be done prior to G-CSF administration)<br>• Vital signs (supine blood pressure, heart rate, respiratory rate, temperature [°C] and room air SPO2)<br>• 12 Lead ECG<br>• CBC with differential<br>• Blood chemistry

Study Procedures	• CD34+ enumeration in PB<br>• Weight (in kg)<br>• Height (in cm)<br>• Pregnancy test – serum or urine for all female patients of childbearing potential<br><br>**Treatment Period**<br>Visit Days 1-3<br>Morning (8:00AM ± 2 hrs): Subjects will undergo mobilization with G-CSF ~10 µg/kg (dose will be calculated according to local SoC and should not exceed 15 µg/kg) subcutaneously (SC).<br>The following procedures will be performed:<br>• AE recording<br>• Concomitant Medications review<br>• Resource use and cost element data collection only if G-CSF is administered at the site (in-patient)<br>• Enrollment with IWRS on Day 1 only[a]<br><br>Visit Day 4<br>Morning (8:00AM ± 2 hours): Subjects will undergo mobilization with G-CSF ~10 µg/kg (dose will be calculated according to local SoC and should not exceed 15 µg/kg) subcutaneously (SC).<br>The following procedures must be done before G-CSF injection, unless otherwise specified.<br>• AE recording<br>• Concomitant Medications review<br>• CBC with differential<br>• Blood chemistry<br>• CD34+ enumeration in PB[b]<br>• Resource use and cost element collection (before and after G-SCF administration)<br>• Randomization using IWRS (only during Part 2, after G-SCF administration)

[a] During Part 1, the IWRS system will be used for eligibility confirmation and stratification parameters entry as no randomization is conducted in Part 1. During Part 2, the IWRS system will provide treatment allocation.

[b] Local assessment in both parts. For Part 2, sample will be analyzed at local and central laboratory.

Study Procedures	<u>Evening of Day 4 (8:00PM ± 2 hours)</u>: Subjects will receive either a single injection of BL-8040 1.25 mg/kg or placebo SC. During Part 1, only BL-8040 (no placebo) will be administered. Premedication should be administered within 1 hour prior to BL-8040/Placebo administration.<br><br>All evening procedures should be done before BL-8040/placebo injection, unless otherwise specified.<br>▪ Directed Physical Exam<br>▪ AE recording<br>▪ Triplicate 12 Lead ECG:<br>a. Pre-dose (up to 1 hour before BL-8040/placebo administration) and<br>b. 30-45 minutes post-dose<br>▪ Vital signs (blood pressure, heart rate, respiratory rate, temperature [°C] and room air SPO2)<br>▪ CBC with differential<br>▪ Blood draw for PK Assessment:<br>a. Pre-dose (up to 1 hour before BL-8040/placebo administration)<br>b. Post dose[a] (40-60 minutes and 2-6 hours)<br>▪ Blood draw for ADA assessment:<br>a. Pre BL-8040/Placebo dosing up to 1 hour before<br>▪ Blood draw for assessment of Complement Activation<br>a. Pre BL-8040/placebo dosing up to 1 hour before<br>b. 2 hours post dosing[a] ±15 minutes<br>▪ Blood draw for assessment of serum tryptase (test for Mast Cell Activation)<br>a. Pre BL-8040/placebo dosing up to 1 hour before dosing<br>b. 2 hours post dosing[a] ±15 minutes<br>c. Only in case of anaphylaxis, unscheduled sample for tryptase will be collected within 2 hours from the event.<br><br><u>Visit Day 5</u><br>*PK assessment*<br>Blood draw for PK assessment: 12 ± 2 hrs post second dose of BL-8040/Placebo administration and before apheresis<br><br><u>Morning (8:00AM ± 2 hours)</u>: A 5th dose of G-CSF will be administered prior to first apheresis. Subjects will then undergo apheresis per institutional protocol (4 blood volumes ±10%/apheresis).

[a] Post dose defined as end time of administration

Study Procedures	The following days of collections and treatments will be conducted provided the threshold of ≥6 x 10^6 CD34+ cells/kg for mobilizations was not reached:<br>The below procedures should be done <u>before</u> G-CSF injection:<br>▪ Directed Physical Exam<br>▪ AE recording<br>▪ Concomitant Medications review<br>▪ Weight<br>▪ Triplicate 12 Lead ECG (up to 1 hour prior to G-CSF)<br>▪ Vital signs<br>▪ CBC with differential<br>▪ Coagulation test<br>▪ Blood chemistry<br>▪ CD34+ enumeration in PB[a]<br>▪ Resource use and cost element data collection (Part 2)<br><br>The below procedures should be done <u>after</u> G-CSF injection:<br>▪ Apheresis - four blood volumes ±10% apheresis – (12 hours ± 2 hours post first dose of BL-8040/Placebo administration) - See section 5.2.4<br>▪ CD34+ enumeration in apheresis product[a]<br>▪ Lymphocyte subsets in apheresis product (Part 2)<br>▪ Multiple Myeloma cells in the apheresis product (Part 2)<br>▪ Resource use and cost element data collection (Part 2)<br><br><u>Visit Day 6</u><br>***This visit will be conducted only for:***<br>▪ ***Subjects who did not reach the goal of collection of ≥6 x 10^6 CD34+ cells/kg for mobilization after one apheresis.***<br>▪ ***<u>All</u> subjects with moderate or severe renal impairment at Screening as defined by GFR <50 mL/min/1.73² calculated by MDRD equation regardless of the amount of cells collected in the first apheresis.***<br><br>**For all patients who <u>did not</u> reach the collection goal in the first apheresis**, a 6th dose of SC G-CSF ~10 µg/kg (and maximum of 15 µg/kg) will be administered prior to the second apheresis in an effort to reach a total of ≥6 x 10^6 CD34+ cells/kg. The following procedures should be performed:<br><u>Morning 8:00AM ± 2 hrs</u>: Subjects will be administered with G-CSF ~10 µg/kg (and maximum of 15 µg/kg) SC

[a] Local assessment in both parts. For Part 2, sample will be analyzed at local and central laboratory.

Study Procedures	The below procedures should be done <u>before</u> G-CSF injection:<br>▪ AE recording<br>▪ Concomitant Medications review<br>▪ CBC with differential<br>▪ Blood chemistry<br>▪ CD34+ enumeration in PB[a]<br>▪ Resource use and cost element data collection (Part 2)<br><br>The below procedures should be done <u>after</u> G-CSF injection:<br>▪ Apheresis - four blood volumes ±10% apheresis - See section 5.2.4<br>▪ CD34+ enumeration in apheresis product[a]<br>▪ Lymphocyte subsets in apheresis product (Part 2)<br>▪ Multiple Myeloma Cells in the apheresis product (Part 2)<br>▪ Resource use and cost element data collection (Part 2)<br><br>**For subjects with moderate or severe renal impairment at Screening**, as defined by GFR <50 mL/min/1.73^2 calculated by MDRD equation, the below procedures should be done:<br>▪ CBC with differential<br>▪ Blood chemistry<br>▪ Vital signs<br>▪ 12 Lead ECG local assessment<br>The results of serum creatinine and GFR, as well as any other clinically significant change, should be reviewed and assessed by the investigator. In case of abnormal clinically significant changes, it should be reported as AE and, in addition to the study procedures, subjects should be followed on a weekly basis until recovery or stabilization by the following procedures:<br>▪ Biochemistry with GFR assessment<br>▪ CBC with differential<br>▪ Vital signs<br>▪ 12 Lead ECG local assessment<br>▪ AE assessment<br>For subjects that did not reach the collection goal and present with BL-8040/placebo related clinically significant changes according to investigator assessment, a <u>second</u> administration will **not** be administered in the evening. The subjects will then be considered as early terminated and Visit 13 (Termination/Early Termination) procedures should be followed.<br>***<u>Of note</u>:*** For patients with moderate or severe renal impairment at Screening, who also did not reach the collection goal, all of the above-listed procedures should be performed (CBC and biochemistry should be done only once).

[a] Local assessment in both parts. For Part 2, sample will be analyzed at local central laboratory.

Study Procedures	***Only if the subject does not reach the collection goal for mobilization (≥6.0 x 10^6 CD34+ cells/kg), a second dose of BL-8040/placebo (in Part 1, only BL-8040 will be administered) will be administered in the evening.***<br>Evening (8:00PM ± 2 hrs):<br>Premedication should be administered within 1 hour prior to BL-8040/Placebo administration.<br>The below procedures should be done before BL-8040/placebo injection:<br>▪ Directed Physical Exam<br>▪ AE recording<br>▪ Triplicate 12 Lead ECG Day 4 evening:<br>a. Pre-dose (up to 1 hour before BL-8040 administration) and<br>b. 30-45 minutes post-dose<br>▪ Vital signs<br>▪ CBC with differential<br>▪ IWRS for cases of a 2nd dose of BL-8040/Placebo<br>▪ Subjects will receive SC injection of BL-8040/placebo<br>▪ Blood draw for PK Assessment:<br>a. Pre-dose (up to 1 hour before BL-8040/placebo administration)<br>b. Post dose[a] (40-60 minutes and 2-6 hours)<br>▪ Resource use and cost element collection (Part 2)<br><br>Visit Day 7<br>This visit will be conducted only in subjects who did not reach the goal of collection after two apheresis procedures and received a second dose of BL-8040/placebo on Visit Day 6.<br><br>*PK assessment*<br>Blood draw for PK assessment: 12 ± 2 hrs post second dose of BL-8040/Placebo administration and before apheresis<br><br>Morning (8:00AM ± 2 hours): A 7th dose of SC G-CSF ~10 µg/kg (and maximum of 15 µg/kg) will be administered prior to a third apheresis.<br>The below procedures should be done before G-CSF injection:<br>▪ Directed Physical Exam<br>▪ Review AE<br>▪ Concomitant Medications review<br>▪ Triplicate 12 Lead ECG<br>▪ Vital signs<br>▪ CBC with differential<br>▪ Coagulation Test<br>▪ Blood chemistry

[a] Post dose defined as end time of administration

Study Procedures	▪ CD34+ enumeration in PB[a]<br>▪ Resource use and cost element data collection (Part 2)<br><br>The below procedures should be done <u>after</u> G-CSF injection:<br>▪ Apheresis - four blood volumes ±10% apheresis - 12 hours ± 2 hours post second dose of BL-8040/Placebo administration) See section 5.2.4<br>▪ CD34+ enumeration in apheresis product[a]<br>▪ Lymphocyte subsets in apheresis product (Part 2)<br>▪ Multiple Myeloma Cells in the apheresis product (Part 2)<br>▪ Resource use and cost element data collection (Part 2)<br><br><u>Visit Day 8</u><br>This visit will be conducted only for:<br>▪ All subjects who did not reach the collection goal for mobilization ($\geq$6.0 x 10^6 CD34+ cells/kg) after three apheresis procedures. In this case an 8th dose of G-CSF will be administered prior to fourth apheresis.<br>▪ All subject with moderate or severe renal impairment at Screening as defined by GFR of <50 mL/min/1.73^2 calculated by MDRD equation, who received a second dose of BL-8040/placebo, regardless of the amount of cells collected in the apheresis.<br><br>**For subjects who <u>did not</u> reach the goal of collection,** the following procedures should be performed:<br><u>Morning (8:00AM ± 2 hours)</u>: A 8th dose of SC G-CSF ~10 µg/kg (and maximum of 15 µg/kg) will be administered prior to a third apheresis.<br>The below procedures should be done <u>before</u> G-CSF injection:<br>▪ Directed physical examination<br>▪ Review AEs<br>▪ Review of Concomitant medications<br>▪ Vital signs<br>▪ CBC with differential<br>▪ Blood chemistry<br>▪ CD34+ enumeration in PB[a]<br>▪ Resource use and cost element data collection (Part 2)

[a] Local assessment in both parts. For Part 2, sample will be analyzed at local and central laboratory.

Study Procedures	The below procedures should be done after G-CSF injection:<br>▪ Apheresis - four blood volumes ±10% apheresis<br>▪ CD34+ enumeration in apheresis product[a]<br>▪ Lymphocyte subsets in apheresis product (only during Part 2)<br>▪ Multiple Myeloma Cells in the apheresis product (only during Part 2)<br>▪ Resource use and cost element data collection (Part 2)<br><br>**For subjects with moderate or severe renal impairment at Screening** as defined by GFR of <50 mL/min/1.73[2] calculated by MDRD equation, the below procedures should be done:<br>▪ CBC with differential<br>▪ Blood chemistry<br>▪ Vital signs<br>▪ 12 Lead ECG local assessment<br>The results of serum creatinine and GFR, as well as any other clinically significant change, should be reviewed and assessed by the investigator. In case of abnormal clinically significant changes, it should be reported as AE and in addition to the study procedures, subjects should be followed on a weekly basis until recovery or stabilization by the following procedures:<br>▪ Biochemistry with GFR assessment<br>▪ CBC with differential<br>▪ Vital signs<br>▪ 12 Lead ECG local assessment<br>▪ AE assessment<br><br>**Rescue procedure**<br>Subjects who fail to collect the cells required per local SoC after four apheresis sessions will be managed per investigator preference in accordance with standard of care guidelines. They may re-attempt mobilization per standard of care after at least 7 days' "rest/wash-out" period from the last dose of BL-8040/placebo. Subjects who failed to collect enough CD34+ cells in four apheresis sessions will continue to be followed according to the protocol schedule visits.<br>All cost element and resource use related to rescue procedure will be collected and captured in the eCRF.

[a] Local assessment in both parts. For Part 2, sample will be analyzed at local and central laboratory.

Study Procedures	<u>Visit 9: Conditioning Treatment Pre-Transplantation</u><br><br>Following successful mobilization and collection of adequate CD34+ cells for auto-HCT, study participants will be admitted to the hospital for preparative (conditioning) chemotherapy. Conditioning must occur within 5 weeks after the last apheresis session<br><br>The procedures and evaluations at Visit 9 should be done within 3 days before beginning of conditioning.<br><br>The pre-transplant conditioning chemotherapeutic regimens (± total body irradiation (TBI)) used in this trial may include:<br>▪ Melphalan (200 or 140 mg/m^2)<br>▪ BEAM<br>▪ BuCy<br>▪ Arsenic/Melphalan<br>▪ Busulfan/Melphalan<br><br>Selection of the appropriate conditioning regimen shall be at the discretion of the Investigator. Local Standard of Care guidelines will be followed.<br><br>The procedures and evaluations at Visit 9 should be done within 3 days before beginning of conditioning:<br>▪ Complete Physical Exam<br>▪ Concomitant Medications review<br>▪ Vital signs<br>▪ CBC with differential<br>▪ Blood Chemistry<br>▪ Blood draw for ADA assessment<br>▪ Pregnancy test (urine/serum)<br>▪ Resources use and cost elements data collection<br><br><u>Visit 10: Transplantation and Immediate Post-Transplantation Follow-Up</u><br><br>Transplantation will occur ~24 hours following the completion of the conditioning regimen and no later than 5 weeks after the last apheresis session.<br><br>Transplantation should be performed using the collected CD34+ cells. A minimum of >2x10^6 CD34+ cells/kg must be transplanted; however, more cells may be transplanted up to the full amount collected at the Investigator's discretion. Excess CD34+ cells may be stored for later use, per Investigator preference.<br><br>Local guidelines for transplantation will be followed.<br><br>Post-transplantation follow-up begins the day after cell transplantation and lasts until the time of neutrophil and platelet engraftment (whichever comes later). This will be assessed by daily CBC of PB (unless engraftment period is conducted as an outpatient - in that case only neutrophils and platelets count will be collected and documented in the CRF together with any usage of medications during this period).

Study Procedures	Engraftment is defined as:<br>• Neutrophil engraftment: Defined as the first of 3 consecutive days of ANC ≥0.5 x 10^9/L or ≥1.0 x 10^9/L for 1 day following the conditioning-regimen-associated nadir.<br>• Platelet engraftment: Defined as the first of 3 consecutive measurements of platelet count ≥20 x 10^9/L following the conditioning regimen associated nadir and without platelet transfusion support for 7 days<br>• *Of note: For subjects receiving transfusion for any reason other than platelet count <20 x 10^9/L, who already met the platelet engraftment criteria as above, this transfusion will not affect the platelets engraftment assessment date (e.g. transfusion for any specific procedures or as part of SoC in specific institutions, etc.)*<br>• Actual engraftment days will be calculated according to CIBMTR guidelines (See Appendix D).<br><br>The procedures and evaluations at Visit 10 are as follows:<br>Day of transplantation - Before Transplant:<br>▪ Directed Physical Exam<br>▪ Concomitant Medications review<br>▪ Vital signs<br>▪ CBC with differential<br>▪ Blood chemistry<br>▪ Resource use and cost elements data collection<br><br>Day 1-29 post-transplant:<br>Procedures to be done daily beginning the day after transplantation until engraftment:<br>▪ Neutrophil and platelets assessment<br>a. For in-patient, CBC with differential will be collected daily and recorded accordingly until engraftment<br>b. For patient that are followed up as out-patient, assessment will be done according to local SoC and recorded within the CRF<br>▪ Recording administration of G-CSF, other growth factors or blood and platelets transfusion will be fully documented within CRF<br>Resources use and cost elements data collection (only for inpatients)<br><br>Visits 11-16<br>Following transplantation, subjects will be monitored for graft durability in PB on the following visit days:<br>▪ Visit 11: Day 30 Post Transplantation (±3 days)<br>▪ Visit 12: Day 60 Post Transplantation (±7 days)<br>▪ Visit 13: Day 100 Post Transplantation, Termination/ Early Termination (± 7 days)

Study Procedures	Long Term Assessment Follow-up Post Transplantation<br>▪ Visit 14: Day 180 (6 months) Post Transplantation (± 14 days)<br>▪ Visit 15: Day 270 (9 months) Post Transplantation (± 14 days)<br>▪ Visit 16: Day 360 (12 months) Post Transplantation (± 14 days)<br><br>Graft durability is defined as maintenance of at least 2 of the following 3 criteria:<br>• Platelet count ≥50,000 (50 x 10^9/L) without transfusion for at least 2 weeks.<br>• Hemoglobin level ≥10 g/dL with no erythropoietin support or transfusions for at least 1 month.<br>• Absolute neutrophil count (ANC) ≥1,000 (1.0 x 10^9/L) for 1 week.<br><br>Bone marrow biopsy will be performed on Day 100 (Visit 13) if there is no biochemical evidence of relapse.<br><br>Visit 11 – Day 30 Post Transplantation (±3 days)<br>The procedures and evaluations will be as follows:<br>▪ Concomitant Medications review- only disease related medications should be reported.<br>▪ Vital signs<br>▪ CBC with differential<br>▪ Blood chemistry<br>▪ Blood Draw for ADA<br>▪ Monoclonal Protein (M) in serum (SPEP) and serum immunofixation<br>▪ Monoclonal Protein (M) in urine (UPEP) and urine immunofixation-24 hours urine collection is required<br>▪ Serum Free Light Chain (FLC)<br>▪ Engraftment assessment by platelets and neutrophils count<br>▪ Resource use and cost elements data collection (only during Part 2)<br><br>Visit 12, Day 60 Post Transplantation (±7 days)<br>The procedures and evaluations will be as follows before starting maintenance therapy and will include:<br>▪ Monoclonal Protein (M) in serum (SPEP) and serum immunofixation<br>▪ Monoclonal Protein (M) in urine (UPEP) and urine immunofixation-24 hours urine collection is required<br>▪ Serum Free Light Chain (FLC)<br>▪ Concomitant Medications review-only disease related medications should be captured.<br>▪ Vital signs<br>▪ ECOG performance status (see Appendix B)<br>▪ CBC with differential

Study Procedures	▪ Blood chemistry<br>▪ Relapse Free Survival (RFS) and overall survival (OS) assessment<br>▪ Resource use and cost elements data collection (only during Part 2)<br><br>**Maintenance Treatment**<br>Subjects can receive maintenance treatment with lenalidomide, bortezomib, pomalidomide or a similar FDA-approved maintenance therapy, beginning on Day 60 Post Transplantation, and according to institute standard of care. Patients should not receive maintenance therapy within the first 60 days from transplantation, except in the case of confirmed disease relapse. Data should be recorded within the CRF in a dedicated page for maintenance treatment.<br><br>Visit 13: Day 100 Post Transplantation, Termination/ Early Termination Visit (±7 days)<br><br>Termination visit procedures:<br>▪ Review of concomitant medications (only disease related medications should be captured)<br>▪ Complete physical examination<br>▪ Vital signs<br>▪ ECOG performance status<br>▪ CBC with differential<br>▪ Blood chemistry<br>▪ Monoclonal Protein (M) in serum (SPEP) and serum immunofixation<br>▪ Monoclonal Protein (M) in urine (UPEP) and urine immunofixation - 24 hours urine collection is required<br>▪ Serum Free Light Chain (FLC)<br>▪ Bone marrow biopsy will be performed on Day 100 (Visit 13) if there is no biochemical evidence of relapse. Relapse Free Survival (RFS) and overall survival (OS) assessment<br>▪ Resource use collection and cost elements data collection (only during Part 2)<br><br>In case of early termination, an Early Termination visit will be performed, and the data will be captured at the termination visit page (Visit 13) of the CRF. All information for early termination, will be documented in the source documents. Only one reason (the most relevant) for early should be recorded in the CRF. If one of the reasons for discontinuation is an AE, this should be chosen as the reason. Patients who discontinue study treatment will continue to be followed as per protocol, unless they specifically withdrew consent for follow-up. Every effort should be made to follow-up these subjects until the end of the study period and certainly until the resolution of the AE.<br><br>Data collection at these visits should primarily be guided by the need to protect subject safety and well-being.

Study Procedures	In case the subject decides to discontinue the follow-up, the reason for discontinuation should be reported at the relevant CRF visit page.<br><br>A patient who received G-CSF and discontinued before receiving BL-8040 could potentially be rescreened assuming wash-out period from G-CSF is completed.<br><br>**Long Term Disease Assessment Follow-Up**<br><br><u>Visits 14-16: Day 180 (6 months) - Day 360 (12 months)</u><br><br>Visits 14-16 will be conducted at 180 days (± 14 days), 270 days (± 14 days) and 360 days (± 14 days) post transplantation, respectively, in order to assess graft durability and disease status. Visit procedures will include:<br>▪ ECOG performance status<br>▪ CBC with differential<br>▪ Blood chemistry<br>▪ Review of concomitant medications (only disease related medications should be captured)<br>▪ Monoclonal Protein (M) in serum (SPEP) and serum immunofixation<br>▪ Monoclonal Protein (M) in urine (UPEP) and urine immunofixation-24 hours urine collection is required<br>▪ Serum Free Light Chain (FLC)<br>▪ Relapse Free Survival (RFS)/Overall Survival (OS) assessment<br><br><u>Visits 17 and up to 5 years follow-up: Overall Survival (OS) Follow-Up</u><br><br>After completing 12 months follow-up visits, subjects will be contacted by phone every 6 months (±30 days) in order to assess disease status for concomitant medications (only disease-related medications should be captured) and assessment for Relapse Free Survival (RFS) and Overall Survival (OS), beginning in Visit 17 at 18 months until 5 years from last patient randomization day.<br><u>Each visit will be performed at a time window of ±30 days</u>
Study Duration	It is expected that the entire study will last for approximately 7.5 years, which comprise:<br>• Part 1: Depending on the number of subjects recruited during Part 1 (10 to 30 subjects), it is estimated that Part 1 will last up to 6 months.<br>• Part 2: The core study recruitment is estimated to take 18-24 months and the study will be concluded at 5 years after the last patient randomization day.
Planned Sample Size	A total of up to 207 subjects will be enrolled into the study:<br>• Period 1 (Part 1) – Will enroll up to 30 subjects.<br>• Period 2 (Part 2) – Will additionally randomize a total of 177 subjects.

Inclusion Criteria	1. Patients must be between the ages of 18 and 78 years.<br>2. Patients must have a signed study informed consent prior to entering the study.<br>3. Histologically confirmed Multiple Myeloma prior to enrollment<br>4. At least one week (7 days) from last induction cycle of combination/multi-agent chemotherapy (e.g. KRD [carfilzomib, lenalidomide, dexamethasone] or VRD [bortezomib, lenalidomide, dexamethasone]) or from last single agent chemotherapy (e.g. lenalidomide, pomalidomide, bortezomib, dexamethasone, etc) prior to the first dose of G-CSF for mobilization.<br>5. Eligible for Autologous Hematopoietic stem cell transplantation according to the Investigator's discretion.<br>6. The subjects should be in first or second CR (including CR and SCR) or PR (including PR and VGPR).<br>7. Eastern Cooperative Oncology Group (ECOG) performance status 0 or 1.<br>8. Adequate organ function at screening as defined below:<br>a. Hematology:<br>• White blood cell counts more than 2.5 x 10^9/L<br>• Absolute neutrophil count more than 1.5 x 10^9/L<br>b. Platelet count more than 100 x10^9/L Renal Function:<br>• GFR value of ≥15 mL/min/1.73^2 calculated by MDRD equation<br>c. Hepatic function:<br>• ALT and/or AST ≤ 2.5 x ULN<br>• Total Bilirubin ≤ 2.0 x ULN unless the subject has Gilbert disease<br>d. Coagulation test:<br>• INR or PT: ≤1.5xULN unless subject is receiving anticoagulant therapy, as long as PT or PTT is within therapeutic range of intended use of anticoagulants<br>• aPTT: ≤1.5xULN unless subject is receiving anticoagulant therapy, as long as PT or PTT is within therapeutic range of intended use of anticoagulants<br>9. Female subjects must be of non-childbearing potential or, if of childbearing potential, must have a negative serum pregnancy test at screening and negative urine/serum pregnancy test within 72 hours prior to G-CSF first administration.<br>10. Women of childbearing potential must agree to use 2 methods of effective contraception: One barrier method (e.g. diaphragm, or condom or sponge, each of which are to be combined with a spermicide) and one hormonal method, unless she uses a highly effective method. Highly effective methods of contraception include:<br>• Combined (estrogen and progestogen containing) hormonal contraception associated with inhibition of ovulation: oral, intravaginal, transdermal<br>• Progestogen-only hormonal contraception associated with inhibition of ovulation: oral, injectable, implantable<br>• Intrauterine device (IUD)

Inclusion Criteria	• Intrauterine hormone-releasing system (IUS)<br>• Bilateral tubal occlusion<br>• Vasectomised partner<br>• Sexual abstinence<br>These methods must be used prior to study entry and for the duration of study participation through 30 days after the last dose of study treatment. Non-childbearing potential is defined as (by other than medical reasons):<br>• ≥45 years of age and has not had menses for over 2 years.<br>• Amenorrheic for > 2 years without a hysterectomy and oophorectomy and a Follicle Stimulating Hormone (FSH) value in the postmenopausal range upon pre-trial (screening) evaluation.<br>• Post hysterectomy, bilateral oophorectomy, bilateral salpingectomy or bilateral tubal ligation at least 6 weeks prior to screening. Documented hysterectomy or oophorectomy must be confirmed with medical records of the actual procedure or confirmed by ultrasound. Tubal ligation must be confirmed with medical records of the actual procedure; otherwise the subject must be willing to use two adequate barrier methods throughout the study, starting with the Screening Visit, through 30 days after the last dose of study drug. Information must be captured appropriately within the site's source documents.<br>11. Male subjects must agree to use an adequate method of contraception starting with the first day of G-CSF administration through 30 days after the last dose of study drug.
Exclusion criteria	1. Previous history of autologous or allogeneic-HCT.<br>2. Failed previous HSC collections or collection attempts.<br>3. Taken any of the listed below concomitant medications, growth factors or stimulating agents within the designated washout period:<br>a. Dexamethasone: 7 days<br>b. Thalidomide: 7 days<br>c. Lenalidomide: 7 days<br>d. Pamolidomide: 7 days<br>e. Bortezomib: 7 days<br>f. Carfilzomib: 7 days<br>g. G-CSF: 14 days<br>h. GM-CSF or Neulasta®: 21 days<br>i. Erythropoietin or erythrocyte stimulating agents: 30 days<br>j. Eltrombopag, romiplostim or platelet stimulating agents: 30 days<br>k. Carmustine (BCNU): 42 days/6 weeks<br>l. Daratumumab: 28 days<br>m. Ixazomib: 7 days<br>4. Received >6 cycles lifetime exposure to thalidomide or lenalidomide.<br>5. Received >8 cycles of alkylating agent combinations<br>6. Received > 6 cycles of melphalan.<br>7. Received prior treatment with radioimmunotherapy, (e.g. radionuclides, holmium).<br>8. Received prior treatment with venetoclax

Exclusion criteria	9. Plans to receive maintenance treatment within 60 days post- transplantation (e.g. lenalidomide, bortezomib, pomalidomide, thalidomide, carfilzomib, etc.).<br>10. Has received a live vaccine within 30 days of the planned start of G-CSF administration. Seasonal flu vaccines that do not contain live virus are permitted.<br>11. Known active CNS metastases or carcinomatous meningitis.<br>12. A history of allergic reactions attributed to compounds of similar chemical or biologic composition to BL-8040, G-CSF, or other agents used in the study.<br>13. Has an active or uncontrolled infection requiring systemic therapy.<br>14. Has a known additional malignancy that is progressing or requires active treatment.<br>15. Has an underlying medical condition that would preclude study participation.<br>16. Is currently participating and receiving study therapy or has participated in a study of an investigational agent and received study therapy or used an investigational device within 4 weeks of the first dose of treatment.<br>17. O_2 saturation < 92% (on room air).<br>18. Personal history or family history of Long QT Syndrome or Torsade de Pointes<br>19. History of unexplained syncope, syncope from an uncorrected cardiac etiology, or family history of sudden cardiac death.<br>20. Myocardial infarction, CABG, coronary or cerebral artery stenting and/or angioplasty, stroke, cardiac surgery, or hospitalization for congestive heart failure within 3 months, Angina Pectoris Class >2 or NYHA Heart Failure Class >2.<br>21. ECG at screening showing QTcF > 470 msec and/or PR > 280 msec.<br>22. Mobitz II 2nd degree AV Block, 2:1 AV Block, High Grade AV Block, or Complete Heart Block, unless the patient has an implanted pacemaker or implantable cardiac defibrillator (ICD) with backup pacing capabilities.<br>23. Has a history or current evidence of any condition, therapy, or laboratory abnormality that might confound the results of the trial, interfere with the subject's participation for the full duration of the trial, or is not in the best interest of the subject to participate, in the opinion of the treating Investigator.<br>24. Has known psychiatric or substance abuse disorders that would interfere with cooperation with the requirements of the trial.<br>25. Is pregnant or breast feeding or expecting to conceive or women of childbearing potential unless consent to use two contraceptive methods or highly effective contraception as detailed above, within the projected duration of the trial, starting with the Screening Visit through 30 days after the last dose of study drug.<br>26. Has a known history of HIV (HIV 1/2 antibodies).<br>27. Has known active Hepatitis B (e.g., Hepatitis B Surface Antigen [HBsAg] reactive) or Hepatitis C (e.g., Hepatitis C Virus [HCV] RNA [qualitative] is detected).<br>28. Untreated or unsuccessfully treated Hepatitis B or C.

Investigational Product Route and Dosage Form	**BL-8040**<br>BL-8040 drug product is formulated as a sterile and non-pyrogenic lyophilized powder in a vial containing 73 mg BL-8040 free base peptide, on dry basis. BL-8040 should be administrated SC upon reconstitution with 2 mL 0.45% Sodium Chloride for Injection (Half Normal Saline).<br>BL-8040 is manufactured in compliance with cGMP by BioConnection (Organon, Kloosterstraat 9, 5349 AB, Oss, Netherlands).<br>**Placebo**<br>BL-8040 Placebo is formulated as a sterile and non-pyrogenic lyophilized powder in a vial containing 50 mg of mannitol. BL-8040 Placebo is administrated SC upon reconstitution with 2 mL 0.45% Sodium Chloride for Injection (Half Normal Saline).<br>BL-8040 Placebo is manufactured in compliance with cGMP by BioConnection (Organon, Kloosterstraat 9, 5349 AB, Oss, Netherlands).<br><br>Packaging and labeling of BL-8040 and Placebo will be performed by Fisher Clinical Services (Steinbühlweg 69, 4123 Allschwil, Switzerland).
Concomitant Medications	Medications specifically prohibited in the Exclusion Criteria will not be allowed during the ongoing trial, unless they are part of local standard of care for treatment in the period between mobilization and engraftment. If there is a clinical indication for any other medication that is not part of standard of care and is specifically prohibited during the trial, approval should be discussed with the Sponsor's Medical Monitor. The final decision on any supportive therapy or vaccination is at the discretion of the Investigator and/or the subject's primary physician. However, the decision to continue the subject's trial therapy requires mutual agreement of the Investigator, the Sponsor, and the subject.<br>The following washout periods prior to the first dose of G-CSF for mobilization are required for the following agents/medications:<br>• Dexamethasone: 7 days<br>• Thalidomide: 7 days<br>• Lenalidomide: 7 days<br>• Pomalidomide: 7 days<br>• Bortezomib: 7 days<br>• Carfilzomib: 7 days<br>• G-CSF: 14 days<br>• GM-CSF or Neulasta®: 21 days<br>• Erythropoietin or erythrocyte stimulating agents: 30 days<br>• Eltrombopag, romiplostim or platelet stimulating agents: 30 days<br>• BCNU: 42 days/6 weeks<br>• Daratumumab: 28 days<br>• Ixazomib: 7 days<br><br>There are no restrictions against any medications except post-transplantation chemotherapy within the 60-days post-transplantation (unless patient is in documented relapse).

<table>
<tr><td>Concomitant Medications</td><td>
<u>Acceptable Concomitant Medications:</u>

All treatments that the Investigator considers necessary for a subject’s welfare may be administered at the discretion of the Investigator in keeping with the community standards of medical care. If changes occur during the trial period, documentation of drug dosage, frequency, route, and date should also be included in the CRF. Starting from Visit 11 and onwards, only disease related medication data will be collected.
All medications and/or nutritional supplements/therapies taken by patients for the duration of study participation will be recorded in the patients’ records and transcribed into the CRF as follows: Documentation of concomitant medications will begin a year prior to screening. Subsequently, patients will be monitored for concomitant medications from the time of the first dose of G-CSF for mobilization until transplantation (inclusive). After transplantation, concomitant medications taken during and between each visit will be recorded only if G-CSF, erythropoietin, alternative stimulating agents and/or transfusions, and/or myeloma-related medications were given, in order to identify any possible medications that may be associated with bone marrow suppression or graft dysfunction.
G-CSF, erythropoietin, alternative stimulating agents and/or transfusions should be reported from the screening and up until 100 days post transplantation.

<u>Prohibited Concomitant Medications/Therapies:</u>
Subjects are prohibited from receiving the following therapies during the Screening period and Treatment Period of this trial:

Those listed previously in the Exclusion Criteria
Antineoplastic systemic chemotherapy or biological therapy
Chemotherapy not specified in this protocol
Investigational agents other than BL-8040
Radiation therapy: radiation therapy to a solitary symptomatic lesion may be considered on an exceptional case by case basis after consultation with the Sponsor.
Maintenance treatment with lenalidomide, bortezomib, pomalidomide, thalidomide, carfilzomib, etc. within 60 days post- transplantation.

If there is a clinical indication for any medication specifically prohibited during the trial, it should be discussed with the Sponsor’s Medical Monitor. Subjects may receive other medications that the Investigator deems to be medically necessary.
There are no prohibited therapies after the 60 days post- transplantation, during the Post-Treatment period Long Term Disease Assessment Follow-up.
</td></tr>
</table>

Concomitant Medications	Pre-Dose Medication and General Injection Instructions:<br>• Premedication with an antihistamine is required for all patients, within one hour prior to study drug administration. The time of administration of premedication is to be recorded on site documentation and in the CRF. The preferred route for administration of premedication is IV.<br>The recommended premedication regimen is: H1 blocker (e.g diphenhydramine, Promethazine, etc) + H2 blocker (e.g. famotidine, ranitidine, etc) + leukotriene inhibitor (e.g. montelukast). This triple-drug combination is required prior to the first dose of BL-8040.<br>• Analgesic medication may be added to the premedication regimen at the discretion of the treating physician.<br>• BL-8040 is to be administered at a dose of 1.25 mg/kg by slow subcutaneous injection of at least 2 minutes per syringe. If the volume to be administered exceeds 2 mL then the injection should be divided into two or more syringes to be administered at different injection locations.<br>• Transient hypotension was witnessed in several cases following the initial treatment with BL-8040. Therefore, caution should be taken with the use of negative chronotropic drugs such as beta blockers. When appropriate, beta blocker should be replaced with non-negative chronotropic drugs.<br>• All patients are to remain under surveillance for 2 hours after the first administration of BL-8040. Patients receiving an additional dose of BL-8040 should remain under observation for 1 hour after the subsequent administration of BL-8040.<br>• **BL-8040 should only be administered when personnel and therapies are immediately available for the treatment of anaphylaxis and other hypersensitivity reactions, should they occur.**
Study Endpoints	**Primary Efficacy Endpoint**<br>Proportion of subjects mobilizing ≥6.0 x 10^6 CD34+ cells/kg with up to 2 apheresis sessions in preparation for auto-HCT after single administration of BL-8040 + G-CSF or placebo + G-CSF.<br><br>**Secondary Efficacy Endpoints**<br>• Proportion of subjects who collect ≥2.0 x 10^6 CD34+ cells/kg in 1 apheresis session.<br>• Proportion of subjects who collect ≥6.0 x 10^6 CD34+ cells/kg in 1 apheresis session.<br><br>**Additional Secondary Efficacy Endpoints**<br>The comparability between the effects of BL-8040 + G-CSF and placebo + G-CSF with the below endpoints will be assessed in a descriptive manner:<br>• Time from transplantation to neutrophil engraftment, defined as ANC ≥0.5 x 10^9/L for 3 consecutive days or ≥1.0 x 10^9/L for 1 day following the conditioning regimen associated nadir.<br>• Time from transplantation to platelet engraftment, defined as the first of 3 consecutive measurements of platelet count ≥20 x 10^9/L without platelet transfusion support for 7 days following the conditioning regimen associated nadir.

Study Endpoints	• Time from transplantation to engraftment, defined as the time to neutrophils and platelets engraftment, whichever comes later.<br>• Graft durability at 60 days post-transplantation.<br>• Graft durability at 100 days post-transplantation.<br>• Graft durability at 6 months post-transplantation.<br>• Graft durability at 12 months post-transplantation<br><br>**Exploratory Endpoints**<br>• Change from Baseline in peripheral blood CD34+ cell concentration on apheresis Day 1 or Day 1 and Day 2, if applicable, after BL-8040/placebo administration.<br>• Incidence of graft failure post-transplant<br>• Overall Survival (OS) during the period of up to 5 years.<br>• Relapse-Free Survival (RFS) during the period of up to 5 years.<br>• Mobilization of Multiple Myeloma cells assessed in the apheresis product after BL-8040/placebo administration.<br>• CD34+ cells collected per kg of actual body weight (ABW) compared to theoretical amount of cells collected based on ideal body weight (IBW)<br><br>**Exploratory Biomarkers**<br>• Immunophenotyping of lymphocyte subsets in apheresis product.<br><br>**Safety and Tolerability**<br>• Incidence of AEs, defined and graded according to the NCI-issued Common Terminology Criteria for Adverse Events (CTCAE).<br>• Incidence of early discontinuation and early discontinuation due to adverse events.<br><br>**Pharmacokinetic Endpoint**<br>• BL-8040 exposure as measured by C_{max} and AUC (See Section 5.7).<br>• Effect of covariates such as body weight and age on BL-8040 exposure.<br>• Exposure-response analysis, if warranted.<br>**Pharmacoeconomic endpoint**<br>The resource utilization up until engraftment, 100 days after transplantation, multiplied with per country unit costs to obtain an average cost per patient per treatment arm.

Statistical Methodology	**General:**<br><br>This Phase III study was designed to meet two sequential goals. In Part 1 of the study, a total of up to 30 subjects will be enrolled to an open-labeled treatment with G-CSF followed by BL-8040 1.25 mg/kg, with the aim of ensuring that 1.25 mg/kg is the optimal BL-8040 dose. This part will be conducted in 3 sequential steps of 10 subjects each and the accumulated data at each step will be reviewed by an independent DMC.<br><br>Following the successful completion of Part 1, a total of 177 subjects will be randomized into Part 2 of the study, which will employ a double-blind placebo-controlled design to assess the safety, tolerability and efficacy of BL-8040 + G-CSF as compared to Placebo + G-CSF.<br><br>One interim analysis for potential early termination of new subject recruitment into the study will be conducted at the time when a total of 65% of the originally planned sample size will be randomized and have completed the stem cell mobilization procedure.<br><br>**Randomization:**<br><br>Following the successful completion of Part 1, subjects will be randomized using a 2:1 ratio to receive BL-8040 + G-CSF or Placebo + G-CSF, respectively. Randomization will use permuted blocks stratifying subjects by remission status (CR or PR) and baseline platelet count ($<200 \times 10^9$/L or $\geq 200 \times 10^9$/L).<br><br>**Interim Analysis, Type-I Error and Multiplicity Adjustment:**<br><br>The overall alpha level for this study is 0.05 using 2-tailed tests. All significance testing for this study will use two-tailed tests.<br><br>One interim analysis for potential early termination of new subject recruitment into the study will be conducted at the time when a total of 65% of the originally planned sample size will be randomized and have completed the stem cell mobilization procedure.<br><br>For the interim analysis, treatment effect will be considered statistically significant if the p-value will be 0.0108678 or less. As the interim analysis will be conducted only for potential early termination of new subject recruitment into the study, the final analysis of primary and secondary endpoints, which will be conducted at the Core Study Termination Visit (Visit 13) that will occur at Day 100 following transplantation (or early terminated before), will require a p-value of 0.0466256. These p-values represent the use of the Lan-DeMets' correction to type-I error.<br><br>Furthermore, one (1) primary endpoint and 2 secondary endpoints are pre-defined for this study. Hence, there will be a total of 3 comparisons for the primary and secondary endpoints altogether. The hierarchical method for multiple endpoint testing for the secondary endpoints will utilize the gate keeping approach at alpha=0.0466256, ensuring that the overall experiment-wise type-I error of 5% is preserved.

Statistical Methodology	**Sample Size Justification for Part 1 (Open Labelled Period for ascertaining the BL-8040 dose optimization):**<br>To ascertain the dose of BL-8040, a total of up to 30 subjects will be enrolled to Part 1 of the study. The population size of Part 1 was determined based on the following assumptions:<br>✓ The endpoint to be used in order to ascertain the BL-8040 dose will be the proportion (%) of subjects mobilizing ≥6.0 x 10^6 CD34+ cells/kg with up to 2 apheresis sessions in preparation for auto-HCT after G-CSF + single administration of BL-8040.<br>✓ It is assumed that the proportion (%) of subjects mobilizing ≥6.0 x 10^6 CD34+ cells/kg with up to 2 apheresis sessions due to treatment with BL-8040 + G-CSF will be 70% or more.<br>✓ Based on DiPersio JF, et al. Blood. 2009;113(23):5720-5726, it is assumed that the null reference hypothesis to be overcome is 35.3%.<br><br>Using SAS® PROC POWER for one binomial proportion at a two-sided alpha level of 5%, we determined that a total of 24 subjects will provide 95% power to demonstrate superiority over the reference value of 35.3%. To account for potential dropouts, the Part 1 population size is further increased to a total of 30 subjects.<br><br>**Sample Size Justification for Part 2 (Randomized, Placebo-Controlled Part):**<br>The power and the derived sample size for Part 2 of the study were calculated based on the following assumptions:<br>✓ The primary study endpoint is the proportion (%) of subjects mobilizing ≥6.0 x 10^6 CD34+ cells/kg with up to 2 apheresis sessions in preparation for auto-HCT after G-CSF + single administration of BL-8040 or Placebo + G-CSF.<br>✓ Subjects will be randomized to treatment with BL-8040 + G-CSF or to Placebo + G-CSF using a 2:1 randomization ratio, respectively.<br>✓ It is assumed that success rates for mobilizing ≥6 x 10^6 cells/kg in MM subjects following treatment with Placebo + G-CSF will be 35%.<br>✓ It is also expected that treatment with BL-8040 will result in a success rate of 70%, in line with results obtained for subjects treated with Mozobil + G-CSF. However, as a worst-case scenario, it is assumed that 20% of subjects assigned to the BL-8040 + G-CSF group would not get their assigned treatment and therefore, using the intention-to-treat (ITT) principle, this would reduce the BL-8040 success rate to 63%.

Statistical Methodology	✓ An additional attrition rate of 20% has also been taken into consideration.<br>✓ Final analysis will employ an alpha level of 0.0466256.<br>✓ SAS® PROC POWER for two proportions was used to determine the sample size.<br><br>According to the above underlying assumptions, a total of 177 subjects will provide 89.8% power to demonstrate that treatment with BL-8040 + G-CSF is superior, at a two-sided alpha of 0.0466256, to treatment with Placebo + G-CSF in the study primary endpoint.<br>The power at interim analysis, assuming an attrition rate of 20% and success rates of 70% and 35% for BL-8040 + G-CSF or Placebo + G-CSF, respectively, is 85.9%.<br><br>**Primary Endpoint and Principal Statistical Analysis:**<br><br>The primary study endpoint is the proportion (%) of subjects mobilizing ≥6.0 x 10^6 CD34+ cells/kg with up to 2 apheresis sessions in preparation for auto-HCT after G-CSF + single administration of BL-8040 or Placebo + G-CSF.<br>The following rules will be incorporated to derive this endpoint:<br>✓ Only subjects randomized to study treatment during Part 2 of the study will be included in the formal efficacy analysis.<br>✓ A subject who cumulatively mobilizes ≥6.0 x 10^6 CD34+ cells/kg with up to 2 apheresis sessions will be classified as a "Responder".<br>✓ Other subjects, including randomized subjects who failed to complete the stem cell mobilization procedure, as well as those who did not mobilize 6.0 x 10^6 CD34+ cells/kg after up to 2 apheresis sessions, will be classified as "Non-Responders".<br>The principal analysis of this binary endpoint will use the Cochran-Mantel-Haenszel test stratified by remission status (CR or PR) and baseline platelet count (<200x10^9/L or ≥200x10^9/L).<br><br>**Sequence of Planned Analyses:**<br><br>Study enrollment may early be terminated in the case that interim analysis will provide statistically significant (p<0.0108678) evidence favoring treatment with BL-8040 + G-CSF. Otherwise, study will continue to randomize up to 177 subjects.<br><br>The sequence of the planned statistical analyses for this study is described below in chronological order:<br><br>Initially, the interim analysis will be conducted at the time when a total of 65% of the planned sample size will be randomized and have completed the stem cell mobilization procedure. A detailed statistical analysis plan (SAP) will be developed while the study is blinded, prior to the conduct of interim analysis.<br><br>The results obtained for this analysis will be used <u>only</u> to determine if study enrollment is to be halted.

Statistical Methodology	Thereafter, the core set of the study accumulated data will be made available for statistical analyses on the day that the last study randomized subject completes the Core Study Termination Visit (Visit 13), which will occur at his/her Day 100 following transplantation (or if early terminated before). At that time the study database will be locked and a formal statistical analysis of the following endpoints will be conducted:<br>✓ Primary Endpoint: Proportion of subjects mobilizing ≥6.0 x 10^6 CD34+ cells/kg with up to 2 apheresis sessions in preparation for auto-HCT after a single administration of BL-8040 + G-CSF or placebo + G-CSF.<br>✓ Secondary Endpoint: Proportion of subjects who collect ≥2.0 x 10^6 CD34+ cells/kg in 1 apheresis session.<br>✓ Secondary Endpoint: Proportion of subjects who collect ≥6.0 x 10^6 CD34+ cells/kg in 1 apheresis session.<br>✓ Additional Secondary Endpoint: Time to neutrophil engraftment, defined as ANC ≥0.5 x 10^9/L for 3 consecutive days or ≥1.0 x 10^9/L for 1 day following the conditioning regimen associated nadir.<br>✓ Additional Secondary Endpoint: Time to platelet engraftment, defined as the first of 3 consecutive measurements of platelet count ≥20 x 10^9/L without platelet transfusion support for 7 days following the conditioning regimen associated nadir.<br>✓ Additional Secondary Endpoint: Time to engraftment defined as the time to neutrophils and platelets engraftment, whichever comes later.<br>✓ Additional Secondary Endpoint: Graft durability at 60 days post-transplantation<br>✓ Additional Secondary Endpoint: Graft durability at 100 days post-transplantation.<br>✓ Economic endpoint: The resource utilization until engraftment, 100 days after transplantation, multiplied with per country unit costs to obtain an average cost per patient per treatment arm.<br>✓ Exploratory Endpoint: Change from Baseline in peripheral blood CD34+ cell concentrations on apheresis Day 1 or Day 1 and 2, if applicable, after BL-8040/placebo single administration.<br>✓ Exploratory Endpoint: Mobilization of Multiple Myeloma cells in the apheresis product.<br>✓ Exploratory endpoint: To descriptively compare the CD34+ cells collected per kg of actual body weight (ABW) used in this study to the theoretical amount of CD34+ cells collected based on ideal body weight (IBW)<br>The results and conclusions obtained from the integrative assessment of these efficacy endpoints, as well as accumulated safety data up to these cut-off dates, will be summarized in a Clinical Study Report (CSR) in support of regulatory approval and local market access of BL-8040 + G-CSF for the treatment of Multiple Myeloma.

Statistical Methodology	BioLineRx believes that unblinding the study following the day that the last randomized subject completes the Core Study Termination Visit, which will occur at his/her Day 100 following transplantation, will not compromise study integrity and blinding and thus will not be associated with any bias for the subsequent sequence of the planned analyses due to the following reasons:<br>✓ Study recruitment, randomization and administration of study IP will already be completed at this study stage.<br>✓ Long-term graft durability endpoints are based on objective measures and therefore are not likely to be influenced from treatment allocation.<br>✓ The individual subject treatment assignment and data listings will only be shared with the Sponsor's dedicated taskforce and with regulatory authorities.<br>✓ The publicly disseminated information will be on a by "group basis".<br>✓ The observed summary statistics of efficacy outcomes will not allow determining individual subject assignment.<br>✓ The observed summary statistics of adverse events will not allow determining individual subject assignment.<br><br>The third set of study accumulated data will be made available for statistical analyses of the long-term graft durability endpoints, as well as the longer-term safety data, on the day the last randomized subject completes Visit 16, which will occur at 12 months following transplantation. At that time, the study database will be locked again and a formal statistical analysis of the following endpoints will be conducted:<br>✓ Additional Secondary Endpoint: Graft durability at 6 months post transplantation.<br>✓ Additional Secondary Endpoint: Graft durability at 12 months post transplantation.<br>✓ Economic endpoint: The resource utilization until engraftment, 100 days post transplantation, multiplied with per country unit costs to obtain an average cost per patient per treatment arm.<br>✓ Exploratory Endpoint: Proportion of subjects with graft failure post-transplantation.<br>The results and conclusions obtained from the integrative assessment of these secondary efficacy endpoints, as well as accumulated safety data up to this cut-off date, will be summarized in supplement to the CSR in further support of regulatory approval of BL-8040 + G-CSF for the treatment of Multiple Myeloma.

Statistical Methodology	The last set of study accumulated data will be made available for statistical analyses of the long-term exploratory endpoints at 5 years following the randomization of the last study subject. At that time, the entire study will be terminated, the study database will be locked again, and a formal statistical analysis of the following endpoints will be conducted:<br>✓ Exploratory Endpoint: Overall Survival (OS) during the period of up to 5 years.<br>✓ Exploratory Endpoint: Relapse-Free Survival (RFS) during the period of up to 5 years.<br>✓ Economic endpoint: The resource utilization until engraftment, 100 days post-transplantation, multiplied with per country unit costs to obtain an average cost per patient per treatment arm. Although no specific resource will be collected after day 100 post-transplantation, clinical endpoints per arm can be combined with unit costs to allow an economic evaluation.<br>The results and conclusions obtained from the integrative assessment of these exploratory efficacy endpoints will be summarized in an additional supplement to the CSR in further support of the regulatory approval of BL-8040 + G-CSF for the treatment of Multiple Myeloma.<br>**Interim Analyses:**<br>An interim analysis for the primary endpoint only will be conducted for potential early termination of new subject recruitment into the study as described above.<br>**Statistical Analysis Plan (SAP):**<br>A more detailed SAP will be developed while the study is blinded, prior to the conduct of the predefined interim analysis.

GLOSSARY

Abbreviation/Term	Definition
ABW	Actual Body Weight
ADA	Anti-drug antibody
ADL	Activities of Daily Living
AE	Adverse event
ALP	Alkaline Phosphatase
ALT (SGPT)	Alanine transaminase (serum glutamate pyruvic transaminase)
ANC	Absolute neutrophil count
aPTT	Activated Partial Thromboplastin Time
Ara-C	Arabinofuranosyl Cytidine / Cytarabine / Cytosine Arabinoside
AST (SGOT)	Aspartate transaminase (serum glutamic oxaloacetic transaminase)
AUC	Area under the curve
Auto-HCT	Autologous Hematopoietic Cells Transplantation
β-HCG	Beta human chorionic gonadotropin
BM	Bone Marrow
BA/BE	Bioavailability / Bioequivalence
BUN	Blood urea nitrogen
CABG	Coronary Artery Bypass Grafting
CBC	Complete blood count
CFR	Code of Federal Regulations
CIBMTR	Center for International Blood and Marrow Transplant Research
C_{max}	Maximum plasma concentration
cGMP	Current Good Manufacturing Practice
CNS	Central nervous system
CR	Complete Response
CrCl	Creatinine Clearance
CRF	Case report form
CRO	Contract Research Organization
CSR	Clinical Study Report
CTCAE	Common Terminology Criteria for Adverse Events
CXCL12	C-X-C motif chemokine ligand 12
CXCR4	CXC Chemokine Receptor Type 4
DFS	disease free survival
DNA	deoxyribonucleic acid
DMC	Data Monitoring Committee
ECG (or EKG)	Electrocardiogram
ECOG	Eastern Cooperative Oncology Group
eCRF	Electronic Case Report Form
EDC	Electronic Data Capture
FACS	Fluorescence-activated cell sorting
FDA	Food and Drug Administration
FLC	Free Light Chains
FSH	Follicle Stimulating Hormone
GCP	Good Clinical Practice

Abbreviation/Term	Definition
G-CSF	Granulocyte Colony-Stimulating Factor
GFR	Glomerular Filtration Rate
HBsAg	Hepatitis B Surface Antigen
HCs	Hematopoietic Cells
HCT	Hematopoietic Cells Transplantation
HCV	Hepatitis C Virus
Hep C Ab	Hepatitis C Antibody
HGB	Hemoglobin
HIV	Human Immunodeficiency Virus
HLT	High Level Term
HSC	Hematopoietic Stem Cell
HSPC	Hematopoietic Stem and Progenitor Cell
IB	Investigator Brochure
IBW	Ideal Body Weight
ICD	Implantable Cardiac Defibrillator
ICF	Informed Consent Form
ICH	International Conference on Harmonization
IEC	Independent Ethics Committee
IND	Investigational New Drug
INN	International Non-proprietary Name
INR	International Normalized Ratio (for blood coagulation tests)
IP	Investigational Product
IRB	Institutional Review Board
ITT	Intention-to-treat
IUD	Intrauterine Device
IUS	Intrauterine Hormone-Releasing System
IV	Intravenous
IWRS	Interactive Web Response System
KRD	Carfilzomib, lenalidomide, and dexamethasone
LDH	Lactate dehydrogenase
MCH	Mean Cell Hemoglobin
MCHC	Mean Cell Hemoglobin Concentration
MCV	Mean Corpuscular Volume
MDRD	Modification of Diet in Renal Disease
MedDRA	Medical Dictionary for Regulatory Activities
mITT	Modified Intention-to-Treat Analysis
MM	Multiple myeloma
MoA	Mechanism of Action
NCI	National Cancer Institute
OS	Overall survival
P1P2	Study Part 1 and Part 2 Analysis Set
PB	Peripheral blood
PCS	Potentially Clinically Significant
PD	Pharmacodynamics
PE	Physical Exam

Abbreviation/Term	Definition
PI	Principal investigator
PK	Pharmacokinetics
POA	Power of Attorney
PP	Per Protocol
PR	Partial Response
PT	Prothrombin Time
PTT	Partial Thromboplastin Time
QA	Quality Assurance
RBC	Red Blood Cell
RFS	Relapse free survival
RNA	Ribonucleic Acid
RR	Response rate
SAE	Serious adverse event
SAP	Statistical Analysis Plan
SC	Subcutaneous
SCR	Stringent complete response
SE	Standard Error
SOC	System Organ Class
SoC	Standard of care
SOP	Standard Operation Procedures
SPEP	Serum protein electrophoresis
ST	Safety Analysis Set
SUSAR	Suspected Unexpected Serious Adverse Reaction
T-ALL	T-cell Acute Lymphoblastic Leukemia
TBI	Total Body Irradiation
TEAE	Treatment Emergent Adverse Event
ULN	Upper limit of normal
UPEP	Urine protein electrophoresis
US	Ultrasound
VGPR	Very Good Partial Response
VP	Vice President
VRD	Bortezomib, lenalidomide, and dexamethasone
WBC	White blood cell (count)
WHO	World Health Organization
λ_z	Elimination rate constant

1. INTRODUCTION

1.1 THERAPEUTIC INDICATION

Multiple myeloma (MM) is a condition in which there is a proliferation of malignant plasma cells derived from a single clone in the bone marrow. MM is the second most common hematologic malignancy[1–3] and accounts for approximately 13% of all hematologic malignancies. The median age of patients at the time of diagnosis is approximately 65 years[4]. Common clinical features of MM are bone pain and pathologic fractures secondary to lytic lesions, renal failure, fatigue secondary to anemia and recurrent infections[5.]

In the past, with conventional chemotherapy (melphalan and prednisone (MP)), the prognosis of MM was a median overall survival of 24 to 30 months. In the 1980s, high-dose chemotherapy and stem-cell rescue therapy via autologous hematopoietic cell transplantation (auto-HCT) was introduced, and randomized trials since then have demonstrated a survival advantage for this modality compared with conventional chemotherapy. The introduction of thalidomide represented a major milestone in the treatment of myeloma, and the subsequent availability of its analogs lenalidomide and pomalidomide, and proteasome inhibitors such as bortezomib and carfilzomib, have expanded therapeutic options further. Currently, median survival data exceed 45 to 60 months with this novel therapy[6–9].

Auto-HCT in MM has been shown to improve event-free survival and overall survival (OS) compared to conventional chemotherapy alone in previously untreated patients with standard-risk MM[10–12]. However, the effectiveness of auto-HCT relies, in part, upon the ability to collect an adequate amount of hematopoietic cells (HCs), typically obtained from peripheral blood (PB). In order to perform an auto-HCT, a minimum of ≥2 x 10^6 CD34+ cells/kg are necessary, while transplants of ≥5-6 x 10^6 CD34+ cells/kg are associated with longer disease-free survival (DFS) and OS compared to lower transplant doses[13–16]. Despite the use of G-CSF to mobilize HCs to the PB and after multiple days of apheresis, ~10-30% of patients remain unable to produce an adequate number of cells for auto-HCT[17–19]. Additionally, multiple apheresis days increase healthcare costs and account for additional logistical burden to both patients and the healthcare system. More recently, small molecules aimed at increasing circulating CD34+ cells have been shown to increase rates of successful collection with shorter collection times[20].

1.2 INVESTIGATIONAL THERAPY

1.2.1 BL-8040

BL-8040 (formerly known as BKT140 and developed by Biokine Therapeutics Ltd.), is a novel selective inhibitor of the CXCR4 chemokine receptor. It is an investigational drug for several indications, including, among others:

- The mobilization of hematopoietic stem cells (HSC) for autologous and/or allogeneic transplantations in subjects undergoing PB stem cell transplantation.
- The treatment of patients with hematologic malignancies or pre-malignant conditions.
- The treatment of patients with solid tumors.
- The treatment of BM failure diseases.

BL-8040 is a 14-residue, cyclic, synthetic peptide capped with an aromatic ring. BL-8040 binds and inhibits the CXCR4 chemokine receptor with high affinity (IC_{50} 0.54 - 4.5 nM). BL-8040 was shown in-vitro and in-vivo to be a specific antagonist of CXCR4 and to have a slow

dissociation rate from the receptor. In in-vivo animal studies, as well as in clinical studies, BL-8040 has demonstrated accelerated mobilization of adult white blood cells (WBCs, neutrophils, monocytes and lymphocytes), normal stem-cells and leukemic blasts from the BM to the PB (see 1.2.1.1).

In-vitro and in-vivo nonclinical studies have shown that, in addition to its activity as a mobilizer of hematopoietic cells, BL-8040 exhibits a CXCR4-dependent preferential anti-tumor effect against malignant cells. In-vivo preclinical studies have also shown that multiple doses of BL-8040 have led to a marked increase in the number of hematopoietic progenitor cells and hematopoietic stem cells (HSCs) in the BM and PB of mice. BL-8040 also promoted increased megakaryopoiesis in the BM, leading to increased platelet production with a prolonged effect in-vivo[21–23].

Detailed information on BL-8040 extensive nonclinical program is presented in the Investigator's brochure (IB).

1.2.1.1 Nonclinical Efficacy

The interaction between CXCL12 and its receptor, CXCR4, is critically involved in the retention of HSPCs (Hematopoietic Stem and Progenitor Cells) and WBCs in the BM. Blocking of CXCR4 may result in the mobilization of these cells to the periphery, as demonstrated in nonclinical studies. The uptake dynamics found in-vitro were corroborated by in-vivo studies in mice, where a dose-dependent mobilization of WBCs (peak at 0.5 hr) to the peripheral blood was observed as a result of a single injection of BL-8040. BL-8040 induced rapid (0.5-2 hrs), dose-dependent and transient mobilization of WBCs, including monocytes, B cells, T cells, progenitors and stem cells in mice treated with BL-8040 as a single agent[21]. The effect was seen in mice at a dose range of 2.5-10 mg/kg, equivalent to the human dose of 0.2-0.83 mg/kg, in respectively.

Single injection of BL-8040 to mice at 6 mg/kg (human equivalent dose of 0.49 mg/kg) induced the mobilization of HSC progenitor cells at 30 minutes and reached a peak 1 to 2 hours post-injection with approximately 10-fold increases over control ($p < 0.01$). Higher doses of BL-8040 alone or in combination with G-CSF were used to induce the mobilization of progenitors and long-term repopulating stem cells with better engraftment abilities (report 140-RD-R-04). Single injection of BL-8040 to mice at a higher dose (12 mg/kg, human equivalent dose of 0.99 mg/kg) showed a robust, prolonged and dose-dependent increase in the number of HSCs mobilized to the blood. A peak in mobilization occurred at 4 hrs post-injection, with approximately 18.6-fold increase from the control), which slowly decreased at 8 hrs post dosing. The effect was still evident at 24 hrs post-injection showing a 3-fold increase from the control.

1.2.1.2 Clinical Experience

These nonclinical results were further supported by findings from two clinical studies: Clinical study BKTSC001 (NCT01010880) and clinical study BL-8040.02 (NCT02073019). In study BKTSC001, which was conducted in MM patients, a single administration of BL-8040, when combined with a standard G-CSF mobilization regimen, facilitated the rapid and robust mobilization of stem cells and WBCs in a BL-8040 dose-dependent manner. At the top two dose levels tested (0.3 and 0.9 mg/kg; equivalent to 0.24 and 0.73 mg/kg free base peptide on dry basis), there was an approximately 4-fold increase in peripheral WBC counts and 10 to 20-fold increase (0.3 and 0.9 mg/kg, respectively) in peripheral CD34+ cells (stem cells).

In study BL-8040.02 a single administration of BL-8040, as a single agent, at doses ranging between 0.5-1 mg/kg (free base peptide on dry basis) to healthy volunteers resulted in rapid and robust mobilization of stem cells and WBCs in a BL-8040 dose-dependent manner. After administration of the first dose of BL-8040, CD34+ counts increased with time, reaching maximal levels between 9 and 11 hours. The maximal counts increased with doses averaging 9.31, 38.2, 43.7 and 45.5 CD34+/µL for placebo, 0.5, 0.75, and 1 mg/kg groups, respectively. By 24 hours, CD34+ levels had slightly declined, but were still 5 to 7-fold higher than Baseline. Upon administration of the second dose, an additional increase in CD34+ was observed, with maximal counts obtained between 4 and 7 hours after dosing. The CD34+ counts declined thereafter, reaching Baseline levels approximately 48 hours after the second dose, suggesting a long PD half-life. On both study days, the PD parameters increased with dose and were higher after the second dose relative to the first dose. After the first dose, a 5 to 10-fold increase from baseline was observed in CD34+ counts.

BL-8040-Related Adverse Events

Full details of the adverse events (AE) seen in each clinical trial are presented in the IB.

Based on the clinical experience to date, the following adverse events may be anticipated following administration of BL-8040: Injection-site reactions (including pain, erythema, pruritus and inflammation) and systemic reactions (hives, pruritus (not at the injection site), flushing, hot flushes, chills, rash, urticaria and hypotension). These two groups of reactions are well managed with pre-medication and/or post event treatment using steroids and antihistamines. Other isolated AEs reported to date include paresthesia, musculoskeletal pain, headache, constipation, diarrhea, abdominal pain, fatigue, nausea, vomiting, increased liver transaminases, increased alkaline phosphatase, dyspnea and hypokalemia. Bone marrow suppression resulting in decreased monocyte count, neutropenia and thrombocytopenia (which in turn may cause petechiae), as well as ataxia and alopecia are expected outcomes of treatment with high dose Ara-C, as administered in study BL-8040.01. BioLineRx has therefore not listed them as anticipated side effects of BL-8040.

Leukocytosis and high and low peripheral blast counts are an expected pharmacodynamic effect of treatment with BL-8040. To date, leukocytosis has been reported in only one patient treated with Nelarabine and BL-8040 for T-ALL.

Most of the AEs observed were transient and mild to moderate in severity. Clinically-appropriate measures should be considered in case of BL-8040-related local reactions at the injection site (e.g., local treatments with steroids and or antihistamines) and systemic reactions (e.g. systemic antihistamines and steroids) with preventive treatment before subsequent doses.

Potential Safety Considerations Based on the BL-8040 MoA

Leukostasis is an important medical event that should be considered based on the pharmacodynamic properties of BL-8040. The promotion of WBCs and HSCs mobilization from the Bone Marrow (BM) may result in leukostasis, the presence of very high WBC counts in the peripheral circulation causing small vessel occlusion with a variety of clinical sequelae including visual symptoms, dyspnea and hypoxia. To date, leukostasis has been reported in only one patient treated with nelarabine and BL-8040 for T-ALL.

1.2.2 G-CSF (Filgrastim)

Filgrastim is a 175 amino-acid protein manufactured by recombinant DNA technology. It is produced by Escherichia coli bacteria into which the human granulocyte colony stimulating

factor gene has been inserted. Filgrastim is unglycosylated and contains an N-terminal methionine necessary for expression in E. coli.

Colony-stimulating factors are glycoproteins that act on hematopoietic cells by binding to specific cell surface receptors and stimulating proliferation, differentiation commitment, and some end-cell functional activation.

Endogenous G-CSF is a lineage-specific colony-stimulating factor which is produced by monocytes, fibroblasts, and endothelial cells. G-CSF regulates the production of neutrophils within the bone marrow and affects neutrophil progenitor proliferation, differentiation, and selected end-cell functional activation (including enhanced phagocytic ability, priming of the cellular metabolism associated with respiratory burst, antibody-dependent killing, and the increased expression of some functions associated with cell surface antigens). G-CSF is not species-specific and has been shown to have minimal direct in-vivo or in-vitro effects on the production of hematopoietic cell types other than the neutrophil lineage.

Absorption and clearance of filgrastim follows first-order pharmacokinetic modeling without apparent concentration dependence. A positive linear correlation occurred between the parenteral dose and both the serum concentration and area-under-the-concentration-time curves. Continuous IV infusion of 20 mcg/kg of filgrastim over 24 hours resulted in mean and median serum concentrations of approximately 48 and 56 ng/mL, respectively. Subcutaneous (SC) administration of 3.45 mcg/kg and 11.5 mcg/kg resulted in maximum serum concentrations of 4 and 49 ng/mL, respectively, within 2 to 8 hours. The volume of distribution averaged 150 mL/kg in both normal subjects and cancer patients. The elimination half-life, in both normal subjects and cancer patients, was approximately 3.5 hours. Clearance rates of filgrastim were approximately 0.5 to 0.7 mL/minute/kg. Single parenteral doses or daily IV doses, over a 14-day period, resulted in comparable half-lives. The half-lives were similar for IV administration (231 minutes, following doses of 34.5 mcg/kg) and for SC administration (210 minutes, following filgrastim doses of 3.45 mcg/kg). Continuous 24-hour IV infusions of 20 mcg/kg over an 11- to 20-day period produced steady-state serum concentrations of filgrastim with no evidence of drug accumulation over the time period investigated.

1.2.3 Transplantation

The following adverse events are related to hematopoietic stem cell transplantation and can be anticipated following myeloablation and further Auto-HCT.

- Alopecia
- Anemia
- Creatinine elevations
- Diarrhea
- Edema
- Fatigue
- Febrile neutropenia
- Gastritis
- Hematuria
- Hemorrhage
- Hypotension
- Hypoxemia
- Incontinence
- Infection / Sepsis

- Infertility
- Insomnia
- Liver function test alterations
- Mental status changes
- Mood alterations
- Mucositis
- Nausea / Vomiting
- Neutropenia
- Pain
- Pleural effusion
- Pneumonitis
- Thrombocytopenia
- Veno-occlusive disease

1.3 STUDY RATIONALE

Autologous hematopoietic cell transplantation (auto-HCT) in multiple myeloma (MM) has been shown to improve overall survival (OS) compared to conventional chemotherapy[10–12]. However, the effectiveness of auto-HCT relies, in part, upon the ability to collect an adequate amount of hematopoietic cells (HCs), typically obtained from peripheral blood (PB). In order to perform an auto-HCT, a minimum of ≥2 x 10^6 CD34+ cells/kg are necessary, while transplants of ≥5-6 x 10^6 CD34+ cells/kg are associated with longer disease-free survival (DFS) and OS compared to lower transplant doses[13–16]. Despite the use of G-CSF to mobilize HCs to the PB and after multiple days of apheresis, ~10-30% of patients remain unable to produce an adequate number of cells for auto-HCT[17–19]. Additionally, multiple apheresis days increase healthcare costs and account for additional logistical burden to both patients and the healthcare system.

In-vitro studies demonstrated that BL-8040 binds and inhibits the CXCR4 chemokine receptor with high affinity. It was shown in-vitro and in-vivo to be a specific antagonist of CXCR4 and to have long receptor occupancy (>48 hrs). In in-vivo animal studies, as well as in clinical study BKTSC001, BL-8040 demonstrated accelerated mobilization of adult WBCs (neutrophils, monocytes, lymphocytes) and HSCs[22].

Despite the use of G-CSF, there remains a significant number of patients who are unable to collect both the minimum number and the optimal number of CD34+ cells/kg prior to auto-HCT. Based on the previous success of BL-8040 to mobilize hematopoietic cells (studies BKTSC001 and BL-8040.02), we propose that the combination of G-CSF and BL-8040 will allow larger proportions of the patient population to collect the minimum and optimal numbers of CD34+ cells/kg, in fewer apheresis sessions.

BL-8040 will first be used in an open label, dose-evaluating, lead-in protocol, followed by a randomized, placebo-controlled 2-arm protocol with the primary endpoint assessing the proportion of patients who collect ≥6 x 10^6 CD34+ cells/kg in 2 apheresis sessions. Secondary endpoints will assess the proportion of patients who collect ≥2 x 10^6 CD34+ and cells/kg or ≥6 x 10^6 CD34+ cells/kg in a single apheresis session. However, any patient not achieving the collecting goal after 2 apheresis sessions, will be allowed to continue apheresis with a second dose of BL-8040/placebo in order to capture a more “difficult-mobilizer”

cohort of patients. Additional secondary endpoints will assess the time to engraftment and graft durability at different time-points.

2. STUDY OBJECTIVES AND ENDPOINTS

2.1 STUDY OBJECTIVES

2.1.1 Primary Objective

The primary objective of the study is to demonstrate the superiority of one dose of BL-8040 + G-CSF over placebo + G-CSF to mobilize ≥6.0 x 10^6 CD34+ cells/kg in up to 2 apheresis sessions in preparation for autologous hematopoietic cell transplantation (auto-HCT) in MM subjects.

2.1.2 Secondary Objectives

The secondary objectives of the study are:

- To demonstrate the superiority of one dose of BL-8040 + G-CSF over placebo + G-CSF to mobilize ≥2.0 x 10^6 CD34+ cells/kg in 1 apheresis session in preparation for auto-HCT in MM subjects.
- To demonstrate the superiority of one dose of BL-8040 + G-CSF over placebo + G-CSF to mobilize ≥6.0 x 10^6 CD34+ cells/kg in 1 apheresis session in preparation for auto-HCT in MM subjects.
- To descriptively assess the comparability between the effects of BL-8040 + G-CSF and placebo + G-CSF in time to neutrophil engraftment, platelet engraftment and the later of the two.
- To descriptively assess the comparability between the effects of BL-8040 + G-CSF and placebo + G-CSF on graft durability at 60 days, 100 days, as well as 6 and 12 months post-transplantation.
- To demonstrate that the use of (one or two doses of) BL-8040 + G-CSF over placebo + G-CSF results in a reduction in resource use and cost savings

2.1.3 Exploratory Objectives

The exploratory objectives of the study are:

- To demonstrate the superiority of BL-8040 + G-CSF over placebo + G-CSF in increasing CD34+ cell concentration in the peripheral blood on apheresis Day 1 or Day 1 and 2, if applicable.
- To descriptively assess the comparability between the effects of BL-8040 + G-CSF and placebo + G-CSF in graft failure post-transplantation.
- To descriptively assess the comparability between the effects of BL-8040 + G-CSF and placebo + G-CSF in overall survival with up to 5 years follow-up.
- To descriptively assess the comparability between the effects of BL-8040 + G-CSF and placebo + G-CSF in relapse free survival with up to 5 years follow-up.
- To descriptively assess the comparability between the effects of BL-8040 + G-CSF and placebo + G-CSF in the mobilization of Multiple Myeloma cells in the apheresis product.

- To descriptively compare the CD34+ cells collected per kg of actual body weight (ABW) used in this study to the theoretical amount of CD34+ cells collected based on ideal body weight (IBW)

2.1.4 Safety and Tolerability Objectives

To demonstrate that the combination of BL-8040 + G-CSF is safe and tolerable as compared to Placebo + G-CSF.

2.1.5 Pharmacokinetic Endpoint

- BL-8040 exposure as measured by Cmax and AUC (See Section 5.6).
- Effect of covariates such as body weight and age on BL-8040 exposure.
- Exposure-response analysis, if warranted.

2.1.6 Pharmacoeconomic Endpoints

The resource utilization up until engraftment, 100 days after transplantation, multiplied with per country unit costs to obtain an average cost per patient per treatment arm.

2.2 STUDY ENDPOINTS/OUTCOMES

2.2.1 Efficacy Endpoints

2.2.1.1 Primary Endpoint

Proportion of subjects mobilizing ≥6.0 x 10^6 CD34+ cells/kg with up to 2 apheresis sessions in preparation for auto-HCT after G-CSF + single administration of BL-8040 or placebo + G-CSF.

2.2.1.2 Secondary Endpoints

- Proportion of subjects who collect ≥2.0 x 10^6 CD34+ cells/kg in 1 apheresis session
- Proportion of subjects who collect ≥6.0 x 10^6 CD34+ cells/kg in 1 apheresis session

2.2.1.3 Additional Secondary Efficacy Endpoints

The comparability between the effects of BL-8040 + G-CSF and placebo + G-CSF the below endpoints will be assessed in a descriptive manner:

- Time from transplantation to neutrophil engraftment, defined as ANC ≥0.5 x 10^9/L for 3 consecutive days or ≥1.0 x 10^9/L for 1 day following the conditioning regimen associated nadir.
- Time from transplantation to platelet engraftment, defined as the first of 3 consecutive measurements of platelet count ≥20 x 10^9/L without platelet transfusion support for 7 days following the conditioning regimen associated nadir.
- Time from transplantation to engraftment, defined as the time to neutrophils and platelets engraftment, whichever comes later.
- Graft durability at 60 days post-transplantation. Visit 12 scheduling may be within ±14 days of Day 60, provided that the visit takes place before starting maintenance therapy.
- Graft durability at 100 days post-transplantation.
- Graft durability at 6 months post-transplantation.

- Graft durability at 12 months post-transplantation.

2.2.1.4 **Exploratory Endpoints**

- Change from Baseline in peripheral blood CD34+ cell concentration on apheresis Day 1 or Day 1 and Day 2, if applicable, after BL-8040/placebo single administration.
- Incidence of graft failure post-transplant.
- Overall survival with up to 5 years follow-up.
- Relapse free survival with up to 5 years follow-up.
- Mobilization of Multiple Myeloma cells assessed in the apheresis product (only from Part 2).
- CD34+ cells collected per kg of actual body weight (ABW) compared to theoretical amount of cells collected based on ideal body weight (IBW).

2.2.2 Exploratory Biomarkers

Immunophenotyping of lymphocyte subsets in apheresis product.

2.2.3 Safety and Tolerability Outcomes

Incidence of AEs, defined and graded according to the NCI-issued Common Terminology Criteria for Adverse Events (CTCAE).

Incidence of early discontinuation and early discontinuation due to adverse events.

2.2.4 Pharmacokinetic Endpoint

- BL-8040 exposure as measured by C_{max} and AUC (See Section 5.6).
- Effect of covariates such as body weight and age on BL-8040 exposure.
- Exposure-response analysis, if warranted.

2.2.5 Pharmacoeconomic Endpoints

The resource utilization up until engraftment, 100 days after transplantation, multiplied with per country unit costs to obtain an average cost per patient per treatment arm

3. STUDY DESIGN

A multicenter, randomized, double-blind, placebo-controlled, parallel group study to evaluate the effect of BL-8040 in combination with G-CSF on mobilizing CD34+ cells. This Phase III study will be composed of two sequential parts, as described hereafter:

3.1 PART 1

This lead-in period is designed to ascertain the dose of BL-8040 and will enroll a total of up to 30 subjects to an open labeled treatment to assess the efficacy, safety and tolerability of the treatment with G-CSF ~10 µg/kg/day (and maximum of 15 µg/kg) and BL-8040 1.25 mg/kg, per study protocol, to achieve the goal collection of $\geq 6 \times 10^6$ CD34+ cells/kg.

An independent data monitoring committee (DMC) will then assess the accumulated data of this lead-in period and recommend whether to continue to Part 2 of the study with BL-8040 1.25 mg/kg. The review of the accumulated data of Part 1 will be conducted in 3 sequential steps:

- Initially, the accumulated data of 10 subjects will be summarized and reviewed by the DMC, which may recommend that Sponsor either study an additional cohort of 10 subjects, lower the dose, discontinue the study or continue to Part 2 of the study.
- Similarly, after reviewing the accumulated data of a total of 20 subjects, the DMC may recommend that the Sponsor either study an additional cohort of 10 subjects, lower the dose, discontinue the study or continue to Part 2 of the study.
- Lastly, after reviewing the accumulated data of a total of 30 subjects, the DMC will make recommendations regarding study continuation.

The below quantitative decision rules will be taken into consideration by the DMC:

- The primary efficacy endpoint of the study is the proportion (%) of subjects mobilizing $\geq 6.0 \times 10^6$ CD34+ cells/kg with up to 2 apheresis sessions in preparation for auto-HCT after G-CSF + single administration of BL-8040 or placebo + G-CSF.
- After the review of the accumulated data of 10 subjects, enroll an additional 10 subjects if the number of subjects meeting primary efficacy endpoint is less than or equal to 6.
- After the review of the accumulated data of 20 subjects, enroll an additional 10 subjects if the number of subjects meeting primary efficacy endpoint is less than or equal to 12.
- After the review of the accumulated data of 30 subjects early terminate the study if the number of subjects meeting primary endpoint is less than or equal to 16.

Furthermore, the DMC will have the freedom to employ more conservative decision rules based on integrative risks and benefits assessments of the accumulated information of the study at each decision point.

All subjects treated during Part 1 will continued to be followed concomitantly to the conduct of Part 2 and according to the same visit schedule until study termination.

3.2 PART 2

Following the successful completion of Part 1, a total of 177 subjects will be randomized into Part 2 of the study, which will employ a randomized, double-blinded, placebo-controlled design to assess the efficacy, safety and tolerability of BL-8040 + G-CSF as compared to Placebo + G-CSF.

One interim analysis for potential early termination of new subject recruitment into the study will be conducted at the time when a total of 65% of the planned sample size will be randomized and have completed the stem cell mobilization procedure.

3.3 BOTH PARTS

The procedure is presented in graphic form in Figure 1.

Each part of the study will consist of the following study periods:

Screening: Within 28 days prior to starting mobilization regimen (Day 1)

Mobilization and Apheresis: From Day 1 to the day before myeloablative chemotherapy (Visit 9); Goal: 3 weeks, maximum: 5 weeks.

Chemotherapy and Transplantation: From first day of myeloablation (Visit 9) to the first day of platelet and neutrophil engraftment (whichever comes later) after transplantation; expected within 2-3 weeks, maximum not defined.

Short-Term Graft Durability: From the first day after engraftment to Day 60 and 100 after transplantation.

Long-Term Graft Durability: From 100 days after transplantation until 12 months after transplantation.

Relapse free survival (RFS): From 12 months after transplantation until a maximal period of 5 years from last patient randomization day.

Overall Survival (OS): From 12 months after transplantation until a maximal period of 5 years from last patient randomization day.

Subjects to be enrolled in Part 2 will be randomized in a 2:1 ratio to receive either BL-8040 or placebo, respectively, plus G-CSF. Randomization will be stratified by remission status (Complete remission (CR) vs. Partial Remission (PR))[a] and baseline platelet count (<200 x 10^9/L or ≥200 x 10^9/L). The apheresis product will be processed and stored according to local practice guidelines at each study center.

Subjects will undergo mobilization with G-CSF ~10 µg/kg (and maximum of 15 µg/kg) subcutaneously (SC) daily in the morning for up to 8 days. Beginning on the evening of Day 4, subjects will receive either a single injection of BL-8040 or placebo SC. In the morning of Day 5, a 5th dose of G-CSF will be administered prior to apheresis. Subjects will then undergo first apheresis per institutional protocol (4 blood volumes ±10% /apheresis). In the event that the subject does not reach the collection goal for mobilization (≥6.0 x 10^6 CD34+ cells/kg), on Day 6, a 6th dose of G-CSF will be administered prior to the second apheresis session in an effort to reach a total of ≥6 x 10^6 CD34+ cells/kg.

If the subject does not reach the collection goal for mobilization (≥6.0 x 10^6 CD34+ cells/kg), a second dose of BL-8040/placebo will be administered in the evening of Day 6 and a 7th dose of G-CSF will be administered in the morning of Day 7 prior to the third apheresis session. If the subject still does not reach the collection goal for mobilization (≥6.0 x 10^6 CD34+ cells/kg), a fourth and final apheresis will be performed on Day 8 with an 8th dose of G-CSF administered prior to the apheresis, in an effort to collect a total of ≥6 x 10^6 CD34+ cells/kg, but at least ≥ 2 x 10^6 CD34+ cells/kg from the combined collections. Subjects who fail to collect the minimum required CD34+ cells/kg will be subsequently managed per investigator preference in accordance with standard of care guidelines. They may reattempt mobilization per standard of care after no less than 7 day "rest/wash-out" period (7 days from the last dose of BL-8040/placebo). Subjects who fail to collect enough CD34+ cells in four apheresis sessions will continue to be followed according to the protocol schedule of visits. The entire study, both Part 1 and Part 2 of the study, will be monitored by an independent DMC according to signed charter in line with regulatory guidelines (see Section 9.7).

[a] The group of Partial Response (PR) will include PR and Very Good PR (VGPR) and the Complete Response (CR) group will include CR and Stringent CR (SCR).

Figure 1: Stem Cell Mobilization Procedure

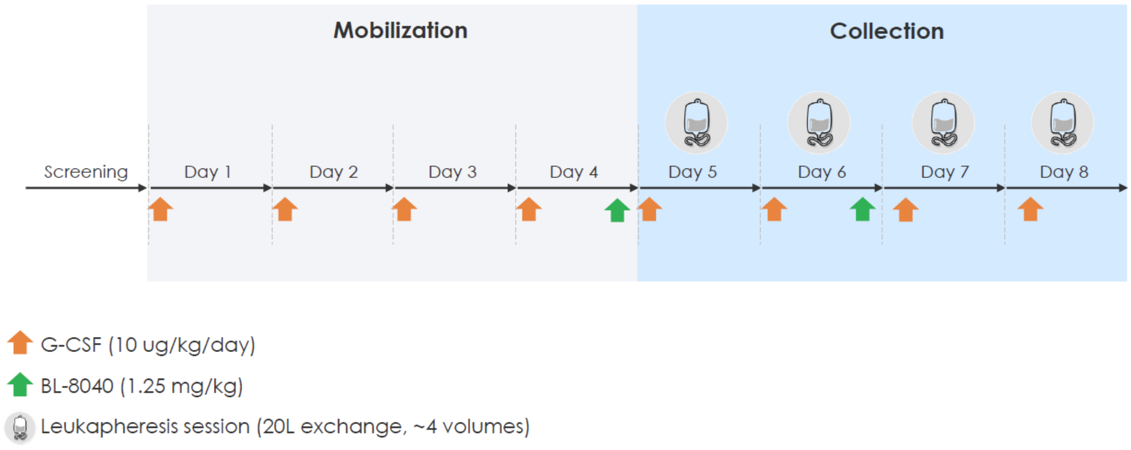

4. STUDY POPULATION

4.1 NUMBER OF SUBJECTS

A total of up to 207 subjects will be enrolled into the study:

- Part 1: Open-label Lead-in – will enroll up to 30 subjects.
- Part 2: Randomized, Double-blinded – will enroll a total of 177 subjects.

4.2 INCLUSION CRITERIA

1. Patients must be between the ages of 18 and 78 years.
2. Patient must have a signed study informed consent prior to entering the study.
3. Histologically confirmed MM prior to enrollment and randomization.
4. At least 1 week (7 days) from last induction cycle of combination/multi-agent chemotherapy (e.g. KRD [carfilzomib, lenalidomide, dexamethasone] or VRD [bortezomib, lenalidomide, dexamethasone]) or last single agent chemotherapy (e.g. lenalidomide, pomalidomide, bortezomib, dexamethasone, etc) prior to the first dose of G-CSF for mobilization.
5. Eligible for Autologous Hematopoietic stem cell transplantation according to the Investigator's discretion.
6. The subjects should be in first or second CR (including CR and SCR) or PR (including PR and VGPR).
7. Eastern Cooperative Oncology Group (ECOG) performance status 0 or 1 (see Appendix B).

8. Adequate organ function at screening as defined below:
   a. Hematology:
      - White blood cell-count more than 2.5 x 10^9/L
      - Absolute neutrophil count more than 1.5 x 10^9/L
      - Platelet count more than 100 x10^9/L
   b. Renal Function:
      - GFR ≥15 mL/min/1.73^2 calculated by MDRD equation
   c. Hepatic function:
      - ALT and/or AST ≤ 2.5 x ULN.
      - Total Bilirubin ≤ 2.0 x ULN unless the subject has Gilbert disease.
   d. Coagulation test:
      - INR or PT: ≤1.5xULN unless subject is receiving anticoagulant therapy, as long as PT or PTT is within therapeutic range of intended use of anticoagulants.
      - aPTT: ≤1.5xULN unless subject is receiving anticoagulant therapy, as long as PT or PTT is within therapeutic range of intended use of anticoagulants.
9. Female subjects must be of non-childbearing potential or, if of childbearing potential, must have a negative serum pregnancy test at screening and negative urine/serum pregnancy test within 72 hours prior to G-CSF first administration.
10. Women of childbearing potential must agree to use 2 methods of effective contraception: One barrier method (e.g. diaphragm, or condom or sponge, each of which are to be combined with a spermicide) and one hormonal method, unless she uses a highly effective method. Highly effective methods of contraception include:

- Combined (estrogen and progestogen containing) hormonal contraception associated with inhibition of ovulation: oral, intravaginal, transdermal
- Progestogen-only hormonal contraception associated with inhibition of ovulation: oral, injectable, implantable
- Intrauterine device (IUD)
- Intrauterine hormone-releasing system (IUS)
- Bilateral tubal occlusion
- Vasectomised partner
- Sexual abstinence.

These methods must be used prior to study entry and for the duration of study participation through 30 days after the last dose of study treatment. Non-childbearing potential is defined as (by other than medical reasons):

- ≥45 years of age and has not had menses for over 2 years.
- Amenorrheic for > 2 years without a hysterectomy and oophorectomy and a Follicle Stimulating Hormone (FSH) value in the postmenopausal range upon pre-trial (screening) evaluation.
- Post hysterectomy, bilateral oophorectomy, bilateral salpingectomy or bilateral tubal ligation at least 6 weeks prior to screening. Documented hysterectomy or oophorectomy must be confirmed with medical records of the actual procedure or confirmed by ultrasound. Tubal ligation must be confirmed with medical records of the actual procedure; otherwise the subject must be willing to use two adequate barrier methods throughout the study, starting with the Screening Visit, through 30 days after the last dose of study drug. Information must be captured appropriately within the site's source documents.

11. Male subjects must agree to use an adequate method of contraception starting with the first day of G-CSF treatment through 30 days after the last dose of study drug.

4.3 EXCLUSION CRITERIA

1. Previous history of autologous or allogeneic-HCT.
2. Failed previous HSC collections or collection attempts.
3. Taken any of the listed below concomitant medications, growth factors or stimulating agents within the designated washout period:
   a. Dexamethasone: 7 days
   b. Thalidomide: 7 days
   c. Lenalidomide: 7 days
   d. Pamolidomide: 7 days
   e. Bortezomib: 7 days
   f. Carfilzomib: 7 days
   g. G-CSF: 14 days
   h. GM-CSF or Neulasta®: 21 days
   i. Erythropoietin or erythrocyte stimulating agents: 30 days
   j. Eltrombopag, romiplostim or platelet stimulating agents: 30 days
   k. Carmustine (BCNU): 42 days/6 weeks
   l. Daratumumab: 28 days
   m. Ixazomib: 7 days
4. Received >6 cycles lifetime exposure to thalidomide or lenalidomide.
5. Received > 8 cycles of alkylating agent combinations.
6. Received > 6 cycles of melphalan.
7. Received prior treatment with radioimmunotherapy, (e.g. radionuclides, holmium).
8. Received prior treatment with venetoclax.

9. Plans to receive maintenance treatment within 60 days post-transplantation (e.g. lenalidomide, bortezomib, pomalidomide, thalidomide, carfilzomib, etc.).
10. Has received a live vaccine within 30 days of the planned start of G-CSF administration. Seasonal flu vaccines that do not contain live virus are permitted.
11. Known active CNS metastases or carcinomatous meningitis.
12. A history of allergic reactions attributed to compounds of similar chemical or biologic composition to BL-8040, G-CSF, or other agents used in the study.
13. Has an active infection requiring systemic therapy or uncontrolled infection.
14. Has a known additional malignancy that is progressing or requires active treatment.
15. Has an underlying medical condition that would preclude study participation.
16. Is currently participating and receiving study therapy or has participated in a study of an investigational agent and received study therapy or used an investigational device within 4 weeks of the first dose of treatment.
17. O_2 saturation < 92% (on room air).
18. Personal history or family history of Long QT Syndrome or Torsade de Pointes.
19. History of unexplained syncope, syncope from an uncorrected cardiac etiology, or family history of sudden cardiac death.
20. Myocardial infarction, CABG, coronary or cerebral artery stenting and /or angioplasty, stroke, cardiac surgery, or hospitalization for congestive heart failure within 3 months or greater than Angina Pectoris Class >2 or NYHA Heart Failure Class >2.
21. ECG at Screening showing QTcF > 470 msec and/or PR > 280 msec.
22. Mobitz II 2nd degree AV Block, 2:1 AV Block, High Grade AV Block, or Complete Heart Block, unless the patient has an implanted pacemaker or implantable cardiac defibrillator (ICD) with backup pacing capabilities.
23. Has a history or current evidence of any condition, therapy, or laboratory abnormality that might confound the results of the trial, interfere with the subject’s participation for the full duration of the trial, or is not in the best interest of the subject to participate, in the opinion of the treating Investigator.
24. Has known psychiatric or substance abuse disorders that would interfere with cooperation with the requirements of the trial.
25. Is pregnant or breast feeding or expecting to conceive or women of childbearing potential unless consent to use two contraceptive methods or highly effective contraception as detailed above, within the projected duration of the trial, starting with the Screening Visit through 30 days after the last dose of study drug.
26. Has a known history of HIV (HIV 1/2 antibodies)
27. Has known active Hepatitis B (e.g., Hepatitis B Surface Antigen [HBsAg] reactive) or Hepatitis C (e.g., Hepatitis C Virus [HCV] RNA [qualitative] is detected).
28. Untreated or unsuccessfully treated Hepatitis B or C.

4.4 SUBJECT IDENTIFICATION

At screening, all subjects who signed informed consent will be identified by a subject number provided during the registration in IWRS system. The subject number will be used throughout the study.

Subjects who fail the screening procedures may be re-screened. Re-screening should include all screening procedures listed in the protocol flow chart, including signature of a new consent form.

Any subject who is re-screened will be assigned a new subject number and his/her previous subject number will be documented in CRF.

4.5 SCREENING FAILURES

Subjects who fail to meet the entrance criteria at any stage during the screening period are defined as screen failures. All screen failures will be documented on the screening log including reason(s) for screen failure. The screening log will be kept in the Investigator's Site File. Screening failures should be registered as failures in the IWRS system and CRF. Reason for screening failure should be captured within CRF and the IWRS system.

Screen failure subjects will not be enrolled to the study and will receive standard of care performed at the site.

4.6 REMOVAL, EARLY WITHDRAWAL OF SUBJECTS FROM THERAPY OR ASSESSMENT

- Subjects are free to discontinue their participation in the study at any time and without prejudice to further treatment. The Investigator must withdraw any subject from the study if that subject requests to be withdrawn, or if it is determined that continuing in the study would result in a significant safety risk to the subject. In urgent cases, the investigator has the ability to unblind the trial treatment immediately. The IWRS system will notify all relevant persons of the unblinding event.
- Subjects withdrawn from the study at any time will not be replaced.
- Subject participation in this study may be discontinued due to the following reasons:
    - Subject withdrew consent
    - Request of the Sponsor
    - Request of the primary care physician or Investigator
    - Non-adherence to study requirements
    - Protocol violation
    - Adverse Event/Experience
    - Loss to follow-up/failure to return
    - Death
    - Pregnancy
    - Other

4.7 HANDLING OF WITHDRAWALS

If a subject is withdrawn from the study, either at his or her request or at the Investigator’s discretion, or if requested by the Sponsor, primary care physician or regulatory agency, or fails to return, every effort should be made to determine the reason. This information will be

recorded on the subject's case report form (CRF) at Visit 13 - Termination/Early Termination All subjects who withdraw from the study prematurely, regardless of cause, should undergo all procedures of the Early Termination Study Visit (see 5.2.7.4). It is vital to obtain follow-up data for any subject withdrawn because of an AE. In any case, every effort must be made to undertake safety follow-up procedures.

If a subject withdraws from the study, all procedures of early termination visit should be performed unless withdrawal occurs after Visit 13.

Subjects will consent to be followed under the same protocol assessments in case of withdrawal, by signing the ICF.

Any serious AE (SAE) must be reported to the Sponsor or Sponsor's designee by telephone, email or fax within 24 hours of becoming aware of the event and to the IRB/IEC according to local regulations (for SAE notification procedures, refer to 7.5).

In the event of any AEs considered to be clinically significant by the Investigator, subjects will be followed up with appropriate medical management until the AE is resolved or stabilized, according to the Investigator's clinical judgment. All follow-up information will be recorded in the subject's CRF until resolution/stabilization of the AE. Subsequent follow-up will be documented in the subject's personal file.

Patients who undergo apheresis on study with CD34+ cells collected, but who either fail to meet the collection goals or withdraw from the study for any of the aforementioned reasons, will continue to be followed as outlined above and under section 5.2.5.

A patient who received G-CSF and discontinued before receiving first BL-8040 could potentially be rescreened assuming washout period from G-CSF is completed.

4.8 Sponsor's Termination of Study

The Sponsor reserves the right to early discontinue the study at any time for any reason.

Regulatory Authorities also have the right to terminate the study for any reason.

5. STUDY PROCEDURES AND ASSESSMENTS

The schedule of events for this study is shown in Appendix A. No protocol-related procedures should be performed before the subject provides written informed consent. Study-related events and activities including specific instructions, procedures, concomitant medications, dispensing of study medication, and descriptions of AEs should be recorded in the appropriate source documents and CRF.

5.1 Screening Period (From the time of Informed Consent Form (ICF) signature until Day 0 (Baseline Visit))

During the Screening Visit, the subject must be thoroughly informed about all aspects of the study, including scheduled study visits and activities, and must sign the informed consent form (ICF; see 9.3).
Prior to any study activities/evaluation, the subject will be allocated a subject number using an Interactive Web Response System (IWRS), which will be used throughout the study, and assessed for eligibility criteria.

A window of up to 28 days is allowed for the screening period procedures. Urinary and blood laboratory assessments, as well as ECG, must be completed within 10 days (+2 days) before G-CSF administration, except blood serology, UPEP, SPEP, immunofixation and FLC. Potential subjects will be assessed for fulfillment of the entry requirements as detailed in the Inclusion and Exclusion criteria (see Sections 4.2 and 4.3).

Screening period procedures:

- ICF process
- Inclusion/exclusion criteria review
- Collect demographics and medical history
- Review prior and concomitant medications
- Complete physical examination
- IWRS
- Adverse Events (AEs) recording starting from ICF signature
- 12 lead electrocardiogram (ECG)
- Vital signs (blood pressure, heart rate, respiratory rate, temperature [°C] and room air SPO2)
- Eastern Cooperative Oncology Group (ECOG) performance status
- Coagulation Test: Prothrombin Time (PT)/International Normalized Ratio (INR) and Activated Partial Thromboplastin Time (aPTT)
- Complete Blood Count (CBC) with differential
- Blood chemistry
- Serum pregnancy test for all female patients of childbearing potential
- Blood Serology Assessment
- Monoclonal Protein (M) in serum (SPEP) and serum immunofixation
- Monoclonal Protein (M) in urine (UPEP) and urine immunofixation-24 hours urine collection is required
- Serum Free Light Chain (FLC)
- Urinalysis
- Bone marrow biopsy – only for CR/sCR patients

Of note: Screening Visit procedures can be performed on the same calendar day as Baseline Visit providing those are performed within 3 days before G-CSF administration

Subjects who failed the screening procedures may be re-screened after consultation with the Sponsor. Re-screening should include all screening procedures listed in the protocol schedule of events, including re-consent signature.

5.2 TREATMENT PERIOD

5.2.1 Baseline Visit (Visit Day 0)

Baseline visit should occur within 28 days after the screening visit and within 3 days prior to G-CSF administration (day -3 to Day 0). Only subjects who fully comply with the inclusion/exclusion criteria for the study will be eligible for participation. The following procedures will be performed at the Baseline Visit. A window of three days (-3 day to Day 0) is allowed for the procedures and assessments of this visit:

- AE recording
- Inclusion/Exclusion criteria review
- Concomitant Medications review
- Directed Physical Examination (if Baseline Visit Day 0 and Visit Day 1 are performed on the same day, Directed PE will be done prior to G-CSF administration)
- Vital signs (supine blood pressure, heart rate, respiratory rate, temperature [°C] and room air SPO2)
- 12 Lead ECG
- CBC with differential
- Blood chemistry
- CD34+ enumeration in PB
- Weight (in kg)
- Height (in cm)
- Pregnancy test – serum or urine for all female patients of childbearing potential

Of note: Baseline Visit Day 0 and Visit Day 1 can be done on the same calendar day providing activities related to Baseline are done before G-CSF administration

5.2.2 Randomization (Part 2 only)

During Part 1, subject numbers will be used to confirm enrollment at Baseline and to update the IWRS. During Part 2, enrollment of subjects will be performed up to 28 days following the screening evaluation, at the Baseline visit after confirmation that subject met study enrolment criteria.

Subjects will be randomized using a 2:1 ratio to receive BL-8040 + G-CSF or Placebo + G-CSF, respectively. Randomization will use permuted blocks stratifying subjects by remission status (CR or PR) and baseline platelet count ($<200 \times 10^9$/L or $\geq 200 \times 10^9$/L). The IWRS will then assign a randomization number and the subject will be supplied and treated with the medication labeled with the applicable kit number.

5.2.3 Schedule of Treatment Period (Day 1 to Day 8)

5.2.3.1 Visit Days 1-3

Morning (8:00AM ± 2 hrs):

- AE recording
- Concomitant Medications review
- Resource use and cost element data collection only for in patient
- Subjects will be administered with G-CSF ~10 µg/kg (and maximum of 15 µg/kg) SC daily
- Enrollment with IWRS in Day 1 only[a]

5.2.3.2 Visit Day 4

Morning (8:00AM ± 2 hours):

Subjects will undergo mobilization with G-CSF ~10 µg/kg (dose will be calculated according to local SoC and should not exceed 15 µg/kg) subcutaneously (SC).

All procedures must be done before G-CSF injection, unless otherwise specified.

- AE recording
- Concomitant Medications review
- CBC with differential
- Blood chemistry
- CD34+ enumeration in PB[b]
- Resource use and cost element collection (before and after G-SCF administration)
- Randomization using IWRS (only during Part 2, after G-SCF administration)

Evening of Day 4 (8:00PM ± 2 hours):

Subjects will receive either a single injection of BL-8040 1.25 mg/kg or placebo SC. During Part 1, only BL-8040 (no placebo) will be administered. Premedication should be administered within 1 hour prior to BL-8040/Placebo administration. All evening procedures should be done before BL-8040/placebo injection, unless otherwise specified.

- Directed Physical Exam
- AE recording

[a] During Part 1, the IWRS system will be used for eligibility confirmation and stratification parameters entry as no randomization is conducted in Part 1. During Part 2, the IWRS system will provide treatment allocation.

[b] Local assessment in both parts. For Part 2, sample will be analyzed at local and central laboratory.

- Triplicate 12 Lead ECG:
    a. Pre-dose (up to 1 hour before BL-8040/placebo administration) and
    b. 30-45 minutes post-dose
- Vital signs (blood pressure, heart rate, respiratory rate, temperature [°C] and room air SPO2)
- CBC with differential
- Blood draw for PK assessment:
    a. Pre-dose (up to 1 hour before BL-8040/placebo administration)
    b. Post dose[a] (40-60 minutes and 2-6 hours)
- Blood draw for ADA assessment (Pre BL-8040/Placebo dosing up to 1 hour before)
- Blood draw for assessment of Complement Activation
    a. Pre BL-8040/placebo dosing up to 1 hour before
    b. 2 hours post dosing[a] ±15 minutes
- Blood draw for assessment of serum tryptase/Mast Cells Activation
    a. Pre BL-8040/placebo dosing up to 1 hour before dosing
    b. 2 hours post dosing[a] ±15 minutes
    c. Only in case of anaphylaxis unscheduled sample for tryptase will be collected within 2 hours from the event
- Subjects will receive a single dose of BL-8040/placebo.

5.2.3.3 **Visit Day 5**

<u>PK assessment</u>

Blood draw for PK assessment: 12 ± 2 hrs post second dose of BL-8040/Placebo administration and before apheresis

Morning (8:00AM ± 2 hours): A 5th dose of G-CSF will be administered prior to first apheresis. Subjects will then undergo apheresis per institutional protocol (4 blood volumes ±10%/apheresis).

The below procedures should be done <u>before</u> G-CSF injection:

- Directed Physical Exam
- AE recording
- Concomitant Medications review
- Weight
- Triplicate 12 Lead ECG (up to 1 hour prior to G-CSF)
- Vital signs

[a] Post dose defined as end time of administration

- CBC with differential
- Coagulation test
- Blood chemistry
- CD34+ enumeration in PB[a]
- Resource use and cost element data collection (Part 2)

The below procedures should be done after G-CSF injection:

- Apheresis - Subjects will then undergo a 4 blood volumes ±10% apheresis (12 hours ± 2 hours post first dose of BL-8040/Placebo administration) - see section 5.2.4)
- CD34+ enumeration in apheresis product[a]
- Lymphocyte subsets in apheresis product (Part 2)
- Multiple Myeloma Cells in the apheresis product (Part 2)
- Resource use and cost element data collection (Part 2)

5.2.3.4 **Visit Day 6**

This visit will be conducted only for:

- Subjects who did not reach the goal of collection of ≥6 x 10^6 CD34+ cells/kg for mobilization after one apheresis.
- All subjects with moderate or severe renal impairment at Screening as defined by GFR <50 mL/min/1.73^2 calculated by MDRD equation regardless of the amount of cells collected in the first apheresis.

For all patients who did not reach the collection goal in the first apheresis, a 6th dose of SC G-CSF ~10 µg/kg (and maximum of 15 µg/kg) will be administered prior to the second apheresis in an effort to reach a total of ≥6 x 10^6 CD34+ cells/kg. The following procedures will be performed:

(Morning 8:00AM ± 2 hrs): Subjects will be administered with G-CSF ~10 µg/kg (and maximum of 15 µg/kg) SC

The below procedures should be done before G-CSF injection:

- AE recording
- Concomitant Medications review
- CBC with differential
- Blood chemistry
- CD34+ enumeration in PB[a]
- Resource use and cost element data collection (Part 2)

[a] Local assessment in both parts. For Part 2, sample will be analyzed at local and central laboratory.

The below procedures should be done after G-CSF injection:

- Apheresis - four blood volumes ±10% apheresis - See section 5.2.4
- CD34+ enumeration in apheresis product[a]
- Lymphocyte subsets in apheresis product (Part 2)
- Multiple Myeloma Cells in the apheresis product (Part 2)
- Resource use and cost element data collection (Part 2)

For subjects with moderate or severe renal impairment at Screening as defined by GFR <50 mL/min/1.73^2 calculated by MDRD equation, the below procedures should be done:

- CBC with differential
- Blood chemistry
- Vital signs
- 12 Lead ECG local assessment

The results of serum creatinine and GFR, as well as any other clinically significant change, should be reviewed and assessed by the investigator. In case of abnormal clinically significant changes, it should be reported as AE and, in addition to the study procedures, subjects should be followed on a weekly basis until recovery or stabilization by the following procedures:

- Biochemistry with GFR assessment
- CBC with differential
- Vital signs
- 12 Lead ECG local assessment
- AE assessment

For subjects that did not reach the collection goal and present with BL-8040/placebo related clinically significant changes according to investigator assessment, a second administration will **not** be administered in the evening. The subjects will then be considered as early terminated and Visit 13 (Termination/Early Termination) procedures should be followed.

Of note: For patients with moderate or severe renal impairment at Screening, who also did not reach the collection goal, all of the above-listed procedures should be performed (CBC and biochemistry should be done only once)

[a] Local assessment in both parts. For Part 2, sample will be analyzed at local and central laboratory.

Only if the subject does not reach the collection goal for mobilization (≥6.0 x 10^6 CD34+ cells/kg), a second dose of BL-8040/placebo (in Part 1, only BL-8040 will be administered) will be administered in the evening.

<u>Evening (8:00PM ± 2 hrs)</u>:

Premedication should be administered within 1 hour prior to BL-8040/Placebo administration.

The below procedures should be done before BL-8040/placebo injection, unless otherwise specified:

- Directed Physical Exam
- AE recording
- Triplicate 12 Lead ECG Day 4 evening:
  a. Pre-dose (up to 1 hour before BL-8040 administration) and
  b. 30-45 minutes post-dose[a]
- Vital signs
- CBC with differential
- IWRS for cases of a 2nd dose of BL-8040/Placebo
- Subjects will receive SC injection of BL-8040/placebo
- Blood draw for PK Assessment:
  a. Pre-dose (up to 1 hour before BL-8040/placebo administration),
  b. Post dose[a] (40-60 minutes and 2-6 hours)
- Resource use and cost element collection (Part 2) (before and after BL-8040/placebo administration)

5.2.3.5 **Visit Day 7**

This visit will be conducted only in subjects who did not reach the goal of collection after two apheresis procedures and received a second dose of BL-8040/placebo on Visit Day 6.

<u>PK assessment</u>

Blood draw for PK assessment: 12 ± 2 hrs post second dose of BL-8040/Placebo administration and before apheresis.

<u>Morning (8:00AM ± 2 hours)</u>: A 7th dose of SC G-CSF ~10 µg/kg (and maximum of 15 µg/kg) will be administered prior to a third apheresis.

[a] Post dose defined as end time of administration

The below procedures should be done before G-CSF injection:

- Directed Physical Exam
- Review AE
- Concomitant Medications review
- Triplicate 12 Lead ECG
- Vital signs
- CBC with differential
- Coagulation Test
- Blood chemistry
- CD34+ enumeration in PB[a]
- Resource use and cost element data collection (Part 2)

The below procedures should be done after G-CSF injection:

- Apheresis - four blood volumes ±10% apheresis (12 hours ± 2 hours post second dose of BL-8040/Placebo administration) - See section 5.2.4
- CD34+ enumeration in apheresis product[a]
- Lymphocyte subsets in apheresis product (Part 2)
- Multiple Myeloma Cells in the apheresis product (Part 2)
- Resource use and cost element data collection (Part 2)

5.2.3.6 **Visit Day 8**

This visit will be conducted only for:

- All subjects who did not reach the collection goal for mobilization ($\geq$6.0 x 10^6 CD34+ cells/kg) after three apheresis procedures. In this case, an 8th dose of G-CSF will be administered prior to fourth apheresis.
- All subject with moderate or severe renal impairment at Screening as defined by GFR of <50 mL/min/1.73^2 calculated by MDRD equation, who received a second dose of BL-8040/placebo, regardless of the amount of cells collected in the apheresis.

For subjects who did not reach the goal of collection the following procedures will be performed:

Morning (8:00AM ± 2 hours): A 8th dose of SC G-CSF ~10 µg/kg (and maximum of 15 µg/kg) will be administered prior to a third apheresis.

[a] Local assessment in both parts. For Part 2, sample will be analyzed at local and central laboratory.

The below procedures should be done before G-CSF injection:

- Directed physical examination
- Review AEs
- Review of Concomitant medications
- Vital signs
- CBC with differential
- Blood chemistry
- CD34+ enumeration in PB[a]
- Resource use and cost element data collection (Part 2)

The below procedures should be done after G-CSF injection:

- Apheresis- four blood volumes ±10% apheresis
- CD34+ enumeration in apheresis product[a]
- Lymphocyte subsets in apheresis product (only during Part 2)
- Multiple Myeloma Cells in the apheresis product (only during Part 2)
- Resource use and cost element data collection (Part 2)

For subjects with moderate or severe renal impairment at Screening as defined by GFR of <50 mL/min/1.73^2 calculated by MDRD equation, the below procedures should be done:

- CBC with differential
- Blood chemistry
- Vital signs
- 12 Lead ECG local assessment

The results of serum creatinine and GFR, as well as any other clinically significant change, should be reviewed and assessed by the investigator. In case of abnormal clinically significant changes, it should be reported as AE and, in addition to the study procedures, subjects should be followed on a weekly basis until recovery or stabilization by the following procedures:

- Biochemistry with GFR assessment
- CBC with differential
- Vital signs
- 12 Lead ECG local assessment
- AE assessment

[a] Local assessment in both parts. For Part 2, sample will be analyzed at local and central laboratory.

Rescue procedure:

Subjects who fail to collect the required CD34+ cells/kg after four apheresis sessions (the requirements will be defined according to local SoC) will be able to receive a rescue procedure (see section 5.2.5) per investigator preference in accordance with standard of care guidelines and may reattempt re-mobilization after at least 7 day “rest/wash-out” period (7 days from the last dose of BL-8040/placebo).

5.2.4 Apheresis procedure

Apheresis will be performed according to local practice guidelines at each study site using a standard 4 blood volumes ±10%. Venous access via an apheresis catheter of the center’s choice will be used and may be prepared/placed in advance of the first apheresis day.

Three samples of apheresis product will be obtained for local and central assessment by FACS analyses (the apheresis product volume and the absolute number of CD34+ cells per unit volume will be measured and recorded in the CRF to calculate the total yield of CD34+ cells/kg).

Processing and storage of apheresis product will be done according to the standardized procedures of each study site.

5.2.5 Rescue Procedure

Subjects who fail to collect the cells required per local SoC after four apheresis sessions will be managed per Investigator preference in accordance with standard of care guidelines. They may re-attempt mobilization per standard of care after at least 7 days “rest/wash-out” period (from the last dose of BL-8040/placebo). Subject who failed to collect enough CD34+ cells in four apheresis sessions will continue to be followed according to the protocol schedule visits. All cost element and resource use related to rescue procedure will be collected and captured in the eCRF.

5.2.6 Post Treatment Period

5.2.6.1 **Visit 9: Conditioning Treatment Pre-transplantation**

Following successful mobilization and collection of adequate CD34+ cells for auto-HCT, study participants will be admitted to the hospital for preparative (conditioning) chemotherapy. Conditioning must occur within 5 weeks after the last apheresis session

The pre-transplant conditioning chemotherapeutic regimens (± total body irradiation (TBI)) used in this trial may include:

- Melphalan (200 or 140 mg/m^2)
- BEAM
- BuCy
- Arsenic/Melphalan
- Busulfan/Melphalan

Selection of the appropriate conditioning regimen shall be at the discretion of the Investigator. Local standard of care guidelines will be followed.

The procedures and evaluations at Visit 9 should be done within 3 days before beginning of conditioning:

- Complete Physical Exam
- Concomitant Medications review
- Vital signs
- CBC with differential
- Blood Chemistry
- Blood draw for ADA assessment
- Pregnancy test (urine/serum)
- Resources use and cost elements data collection

5.2.6.2 Visit 10: Transplantation and Immediate Post-Transplantation Follow-Up (Day 1-29 Post Transplantation)

Transplantation will occur ~24 hrs after the completion of the conditioning regimen and no later than 5 weeks after the last apheresis session.

Transplantation should be performed using the collected CD34+ cells. A minimum of >2 x 10^6 CD34+ cells/kg must be transplanted; however, more cells may be transplanted up to the full amount collected at the Investigator's discretion. Excess CD34+ cells may be stored for later use, per Investigator preference. The number of cells transplanted will be recorded in the CRF.

Local guidelines for transplantation will be followed.

Post-transplantation follow-up begins the day after cell transplantation and lasts until the time of neutrophil and platelet engraftment (whichever comes later). Engraftment will be assessed by daily CBC of PB unless engraftment period is conducted as an outpatient – in that case only neutrophils and platelets counts will be collected and documented in the CRF together with any usage of medications during this period.

Engraftment is defined as follows:

- **Neutrophil engraftment:** Defined as the first of 3 consecutive days of ANC ≥0.5 x 10^9/L or ≥1.0 x 10^9/L for 1 day following the conditioning-regimen-associated nadir.
- **Platelet engraftment:** Defined as the first of 3 consecutive measurements of platelet count ≥20 x 10^9/L following the conditioning regimen associated nadir and without platelet transfusion support for 7 days
- *Of note: For subjects receiving transfusion for any reason other than platelet count <20 x 10^9/L, who already meet the platelet engraftment criteria as above, this transfusion will not affect the platelets engraftment assessment date (e.g. transfusion for any specific procedures or as part of SoC in specific institutions, etc.)*

- Actual engraftment days will be calculated according to CIBMTR guidelines (See Appendix D).

The procedures and evaluations at Visit 10 are as follows:

- Day of transplantation - Before Transplant
  - Directed Physical Exam
  - Concomitant Medications review
  - Vital signs
  - CBC with differential
  - Blood chemistry
  - Resources use and cost elements data collection

- Day 1-29 post-transplant:
  Procedures to be done daily beginning the day after transplantation until engraftment:
  - Neutrophil and platelets assessment
    a. For in-patient, CBC with differential will be collected daily and recorded accordingly until engraftment
    b. For patient that are followed up as out-patient, assessment will be done according to local SoC and recorded within the CRF
  - Recording administration of G-CSF, other growth factors or blood and platelets transfusion will be fully documented within CRF
  - Resources use and cost elements data collection (only for inpatients)

5.2.7 Visit 11-13: Graft Durability and Core Study Termination Visit

Following transplantation, subjects will be monitored for graft durability in PB. Counting begins on transplantation day, which is considered as Day 0.

Graft durability is defined as maintenance of at least 2 of the following 3 criteria:

- Platelet count ≥50,000 (50 x 10^9/L) without transfusion for at least 2 weeks.
- Hemoglobin level ≥10 g/dL with no erythropoietin support or transfusions for at least 1 month.
- Absolute neutrophil count (ANC) ≥1,000 (1.0 x 10^9/L) for 1 week.

Bone marrow biopsy will be performed on Day 100 (Visit 13) if there is no biochemical evidence of relapse.

5.2.7.1 Visit 11: Day 30 Post Transplantation (± 3 days)

The procedures and evaluations will be as follows:

- Concomitant Medications review - only disease related medications should be reported.
- Vital signs
- CBC with differential
- Blood chemistry
- Blood Draw for ADA
- Monoclonal Protein (M) in serum (SPEP) and serum immunofixation
- Monoclonal Protein (M) in urine (UPEP) and urine immunofixation - 24 hours urine collection is required
- Serum Free Light Chain (FLC)
- Engraftment assessment by platelets and neutrophils count
- Resource use and cost elements data collection (only during Part 2)

5.2.7.2 Visit 12: Day 60 Post Transplantation (±7 days)

Visit procedures will include as follows:

- Monoclonal Protein (M) in serum (SPEP) and serum immunofixation
- Monoclonal Protein (M) in urine (UPEP) and urine immunofixation-24 hours urine collection is required
- Serum Free Light Chain (FLC)
- Concomitant Medications review-only disease related medications should be captured.
- Vital signs
- ECOG performance status (see Appendix B)
- CBC with differential
- Blood chemistry
- Relapse Free Survival (RFS) and overall survival (OS) assessment
- Resources use and cost elements data collection (only during Part 2)

5.2.7.3 Maintenance Treatment

Subjects will be allowed to receive maintenance treatment with lenalidomide, bortezomib, pomalidomide or a similar FDA-approved maintenance therapy, beginning on Day 60 post transplantation. Patients should not receive maintenance therapy within the first 60 days from transplantation, except in the case of confirmed disease relapse. Data should be recorded within the CRF in a special assigned page for maintenance treatment.

5.2.7.4 **Visit 13: Day 100 Post Transplantation (± 7 days), Termination/Early Termination Visit**

Termination visit procedures:

- Concomitant Medications review- only disease-related medications should be captured.
- Complete physical examination
- Vital signs
- ECOG performance status (see Appendix B)
- CBC with differential
- Blood chemistry
- Monoclonal Protein (M) in serum (SPEP) and serum immunofixation
- Monoclonal Protein (M) in urine (UPEP) and urine immunofixation-24 hours urine collection is required
- Serum Free Light Chain (FLC)
- Bone marrow biopsy will be performed on Day 100 (Visit 13) if there is no biochemical evidence of relapse. Relapse Free Survival (RFS) and overall survival (OS) assessment
- Resources use and cost elements data collection (only during Part 2)

All information for early termination will be documented in the source documents. Only one reason (the most severe) for early discontinuation should be recorded in the CRF. If one of the reasons for discontinuation is an AE, this should be chosen as the reason. Patients who discontinue study treatment will continue to be followed as per protocol in case they agreed to it in the ICF. Every effort should be made to follow-up these subjects until resolution of the AE and for the entire period of the study follow-up (up to 5 years).

Data collection at these visits should primarily be guided by the need to protect subject safety and well-being.

In case the subject decides to discontinue the follow-up, the reason for discontinuation should be reported at the relevant CRF visit page.

A patient who received G-CSF and discontinued before receiving BL-8040 could potentially be rescreened assuming washout period from G-CSF is completed.

5.2.8 Long Term Disease Assessment Follow-Up Post Transplantation

5.2.8.1 Visits 14-16: Day 180 (6 months) - Day 360 (12 months)

Visits 14-16 will be conducted at 180 days (±14 days), 270 days (±14 days) and 360 days (±14 days) post-transplantation. Visit procedures will include:

- ECOG performance status
- CBC with differential
- Blood chemistry
- Review of Concomitant Medications - only disease-related medications should be captured.
- Monoclonal Protein (M) in serum (SPEP) and serum immunofixation
- Monoclonal Protein (M) in urine (UPEP) and urine immunofixation - 24 hours urine collection is required
- Serum free light chain (FLC)
- Relapse Free Survival (RFS) and overall survival (OS) assessment

5.2.8.2 Visit 17 and up to 5 years

After completing 12 months follow-up visits, subjects will be contacted by phone every 6 months (± 30 days) in order to assess disease status, concomitant medications (only disease-related medications should be captured) and assessment for Relapse Free Survival (RFS) and Overall Survival (OS), beginning in Visit 17 at 18 months until 5 years from last patient randomization day.

5.2.8.3 Unscheduled Visits

An unscheduled visit may be performed at any time of the study at the subject's request or as deemed necessary by the Investigator. The date and reason for the unscheduled visit will be recorded. AE monitoring and concomitant medication recording will be performed by the Investigator. Other procedures and evaluations will be completed as deemed necessary by the Investigator and may include (but not be limited to) laboratory safety tests, vital signs and physical examination.

5.3 SAFETY ASSESSMENTS

Safety assessments will be based on changes from Baseline of clinical signs and symptoms reported by the subject or observed by the Investigator, including AEs, concomitant medication use, treatment compliance, tolerability (e.g. dropouts due to AEs), vital signs, ECGs, physical examination, laboratory safety assessments and clinical evaluation of leukostasis.

5.3.1 Adverse Events (AEs)

All AEs will be collected starting from ICF signature and up to 30 days from the last dose of study drug.

Any new systemic AE that occurs between scheduled assessment visits should be brought to the attention of the Investigator and recorded in the subject's medical file and on the appropriate CRF page.

AEs will be reported and graded in accordance with the latest NCI-CTCAE version (currently version 5.0) and coded by Data Management using the latest version of MedDRA (currently version 20.1).

All information on use of resources to manage the AE will be collected. This includes drug use, time of the physician and nurse, consumables, drugs, imaging and laboratory tests.

Post-conditioning and transplantation neutropenia and thrombocytopenia are expected findings and therefore there is no need to report them as adverse events unless the severity is not as expected with these procedures according to PI assessment. Concomitant medication for those events should be recorded in concomitant medication page in CRF.

5.3.2 Concomitant Medications

Concomitant medication use will be recorded at the Screening visit and will include medications taking by the patient within 12 months before screening.

All concomitant medication will be recorded up to Visit 10, inclusive.

Starting from Visit 11, only myeloma/disease-related Concomitant Medications will be recorded, including among others stimulating factors, transfusions, myeloma-specific chemotherapy, etc.

5.3.3 Vital Signs

During Part 1, the lead-in period, as well as Part 2, vital signs will be measured at:

- Screening
- Baseline
- Day 4, evening, before BL-8040/placebo administration
- Day 5, morning, before G-CSF administration
- Day 6, morning before G-CSF administration
- Day 6, evening, before BL-8040/placebo administration
- Day 7 and Day 8, morning, before G-CSF administration

Vital signs will also be measured at:

- Conditioning visit (Visit 9), before procedure
- Transplantation day, before procedure
- Day 30 Post-Transplantation (± 3 days)
- Day 60 Post-Transplantation (± 7 days)
- Day 100 Post-transplantation (± 7 days)
- Day 6 (morning) for subjects with moderate or severe renal impairment
- Day 8 (morning) for subjects with moderate or severe renal impairment, who received a second dose of BL-8040/placebo.

Vital signs will include blood pressure, pulse rate, body temperature, oxygen saturation and respiration rate after at least 5 minutes rest as per standard practice at the investigational site. Significant findings noticed after the start of study drug, which meet the definition of an AE, must be recorded on the AE CRF.

5.3.4 Electrocardiogram

- During Part 1, the lead-in period, as well as Part 2, ECG will be taken at Screening and at Baseline (Day 0).
- On Day 4, evening, ECG will be taken in triplicate prior to study drug administration (up to 1 hour pre-dose) and 30-45 minutes post dose[a]
- On Day 5, morning, ECG will be taken in triplicate prior to apheresis and G-CSF administration (up to 1 hour pre G-CSF dose)
- On Day 6, evening, ECG will be taken in triplicate prior to study drug administration (up to 1 hour pre-dose) and 30-45 minutes post dose[a]
- On Day 7, morning, ECG will be taken in triplicate prior to apheresis and G-CSF administration (up to 1 hour pre G-CSF dose).
- On Day 6, morning, ECG will be taken in triplicate for subjects with moderate or severe renal impairment
- On day 8, morning, ECG will be taken in triplicate for subjects with moderate or severe renal impairment if who received a second dose of BL-8040/placebo

The subject should rest for at least 10 minutes before measurement is taken. In cases were ECG coincide with blood collection or vital signs measurement, ECG recording should be performed first.

Interval between triplicate ECG is one minute.

[a] Post dose defined as end time of administration

The following assessment will be done as part of the ECG:

- Ventricular rate / Heart rate (beats per minute)
- RR interval (milliseconds)
- PR interval (milliseconds)
- QRS duration (milliseconds)
- QT interval (milliseconds)
- QTcF interval (milliseconds)

ECG printouts will be evaluated by the Investigator or designee, signed and dated and filed in the source documentation file. All ECG but those of Day 6 and 8 for subjects with moderate or severe renal impairment will be sent automatically to a central reading center. In case of clinically significant pathological finding an alert will be sent to each site and to the Sponsor Medical monitor.

Day 6 and 8 ECGs for subjects with moderate or severe renal impairment will be done locally evaluated by the Investigator or designee, signed and dated and filed in the source documentation file.

5.3.5 During Parts 1 and 2

Complete Physical Examination

Will include assessment of head, lungs, cardiovascular system, abdomen, musculoskeletal system, skin, lymph nodes, neurological system and, where appropriate, other body systems as indicated in the study schedule.

A complete physical examination will be conducted at Screening, Visit 9 (Conditioning) and Visit 13 (Termination/Early Termination visits).

Directed Physical Examination

During a directed PE, particular attention should be focused on identifying possibly G-CSF and study drug related adverse events and managing these AEs effectively.

A directed physical examination will be conducted:

- at Baseline (Day 0).

  In case Baseline visit Day 0 and Visit Day 1 performed on the same day, Directed PE will be done prior G-CSF administration.
- on Day 4 before BL-8040/placebo administration.
- on Day 5 before G-CSF administration.
- on Day 6 before BL-8040/placebo administration.
- on Days 7 and 8 before G-CSF administration.
- on Transplantation day prior to transplantation.
- Day 30 Post-Transplantation (± 3 days).

Information about the physical examination must be presented in the source documentation at the study site. Significant findings that are present prior to the start of study drug must be included in the Relevant Medical History/ Current Medical Conditions CRF. Significant findings or deterioration of pre-existing condition made after ICF signing that meet the definition of an AE must be recorded on the AE CRF module if occurred until Day 30 post treatment.

5.3.6 Laboratory Assessments

Complete CBC and differential

During Parts 1 and 2, CBC (see Table 1) will be performed by local laboratories at the participating sites at:

- Screening
- Baseline (Visit Day 0)
- Day 4, morning and evening, pre-dose (of G-CSF and study drug administration, respectively)
- Day 5, morning pre-dose (of G-CSF and study drug administration, respectively)
- Day 6, morning and evening, pre-dose (of G-CSF and study drug administration, respectively)
- Days 7 and 8, morning, G-CSF pre-dose (of G-CSF At Visit 9 - Conditioning visit prior to treatment
- At Visit 10 - Transplantation day
- Visits 12-16:
    - Day 30 Post-transplantation (± 3 day)
    - Day 60 Post-Transplantation (± 7 days)
    - Day 100 Post-Transplantation (± 7 days)
    - Day 180 Post-Transplantation (± 14 days)
    - Day 270 Post-Transplantation (± 14 days)
    - 1 Year Post-Transplantation (± 14 days)
- For subjects with moderate or severe renal impairment, on Day 6, morning, and Day 8, morning (Day 8 only if a second dose of BL-8040/Placebo was administered)

Neutrophils and Platelets assessment only

- Day 1– Day 29 Post-Transplant or until engraftment, whichever occurs first. For in-patient, assessment should be performed on a daily basis; for out-patient, assessment should be performed according to local SoC.

Blood Chemistry

Blood chemistry laboratory tests (see Table 1) will be taken on:

- Screening
- Baseline (Visit Day 0)
- Day 4, morning, before G-CSF administration
- Days 5, 6, 7, and 8, morning, before G-CSF administration

- At Visit 9 - Conditioning visit
- At Visit 10 - Transplantation day
- Visits 12-16:
  - Day 30 Post-transplantation (± 3 day)
  - Day 60 Post-Transplantation (± 7 days)
  - Day 100 Post-Transplantation (± 7 days)
  - Day 180 Post-Transplantation (± 14 days)
  - Day 270 Post-Transplantation (± 14 days)
  - 1 Year Post-Transplantation (± 14 days)
- For subjects with moderate or severe renal impairment, on Day 6, morning, and Day 8, morning (Day 8 only if a second dose of BL-8040/Placebo was administered)

Coagulation Test

INR and PT/aPTT will be performed at Screening and on Days 5 and 7, before apheresis.

Other assessments

- Blood Serology assessment will be performed at Screening.
- Monoclonal (M) Protein in Serum, Monoclonal (M) Protein in Urine (i.e. UPEP/SPEP with Immunofixation) and Serum Free Light Chain (FLC) will be taken at Screening, Day 30, 60, 100, 180, and Day 270, and 12 months post-transplantation.
- Urinalysis will be done at Screening visit.
- Laboratory safety sampling will include the parameters listed below. The exact time-points for each one of the tests are specified in Appendix A.

Table 1: Laboratory Assessments

Evaluations	Parameters
Hematology	Red blood cell count, hemoglobin (HGB), hematocrit (HCT), mean cell hemoglobin (MCH), mean cell hemoglobin concentration (MCHC), mean corpuscular volume (MCV), white blood cell (WBC) count and differential (including number of blasts, neutrophils and lymphocytes) and platelet count.
Chemistry	Electrolytes: sodium (Na+), potassium (K+), calcium and phosphorus<br>Liver function tests: AST, ALT, ALP, total bilirubin<br>Kidney function tests: creatinine, BUN<br>Other: glucose, uric acid, LDH.
Coagulation	Pro-thrombin time (PT)/INR and activated partial thromboplastin time (aPTT)
Serology[a]	HIV antibodies (HIV1 and HIV2), Hepatitis B surface antigen (HBsAg) and Hepatitis C antibody (Hep C Ab).
Urinalysis	Bacteria, Glucose, protein, Bilirubin, Leukocyte, RBC, Cast, Ketones, Specific Gravity, Color, Nitrites, Crystals, pH.
Other	Pregnancy test - a serum/urine β-HCG pregnancy test for women of childbearing potential.

[a] To be collected at screening only.

Laboratory safety test abnormalities, which arise after study drug administration, will be repeated as clinically indicated until the values return to normal or until the etiology has been determined and the condition considered stable. Abnormal laboratory test results that are considered to be clinically significant by the Investigator and are up to 30 days from the last dose of study drug should be reported as an AE in the AE CRF module.

Laboratory results will be reported to the Investigator or designee who will review, sign and date abnormal laboratory findings for clinical significance. The Investigator will note any laboratory test results of clinical concern or values that were outside normal ranges and provide details of the relationship to investigational product and the action taken. If a change in a laboratory value represents a medical condition, the medical condition will be listed in the AE record.

5.3.7 Assessment of Anti-Drug Antibodies (ADA), Complement Activation and Mast Cell Activation

Tests for mast cell activation and complement activation will be performed by the central laboratory and will be collected on Day 4 (evening) - before study drug administration (up to 1 hour pre-dose), and 2 hours following administration[a] (±15 minutes). Testing will be performed in Part 1 and Part 2 of the study. Details are available in the study "Laboratory Manual".

Testing for ADA will be performed by a central laboratory and will be collected on Day 4 (evening) - before BL-8040 administration (up to 1 hour pre-dose), on Visit 9 before chemotherapy administration and on Visit 11.

5.4 EFFICACY ASSESSMENTS

5.4.1 CD34+ Cell Enumeration in the Apheresis Product

CD34+ cell enumeration in the apheresis product will be performed both by the local and central laboratories (Part 2), once a day, on Days 5-8. Decisions with regard to continuing apheresis procedures will be made based on local laboratory results.

5.4.2 CD34+ Cell Enumeration in Peripheral Blood (PB)

CD34+ cell enumeration in PB should be collected before G-CSF administration. This assessment will be performed at local laboratory during Parts 1 and 2 and will also be performed by central laboratory during Part 2 only. The assessment will be done at Baseline and once a day on Days 4-8.

---

[a] Post dose defined as end time of administration

5.4.3 Assessment of Engraftment by CBC

Hematology laboratory tests for the purposes of safety and assessment of engraftment will be taken:

- At Visit 9 - Conditioning visit
- At Visit 10:
  - At Transplantation day
  - Day 1 – Day 29 Post-Transplantation (for out-patients, assessment will be performed according to local SoC) - only neutrophils and platelets will be recorded during this period
- Visits 12-16:
  - Day 30 Post-transplantation (± 3 days)
  - Day 60 Post-Transplantation (± 7 days)
  - Day 100 Post-Transplantation (± 7 days)
  - Day 180 Post-Transplantation (± 14 days)
  - Day 270 Post-Transplantation (± 14 days)
  - 1 Year Post-Transplantation (± 14 days)

5.5 Disease Status Assessment by Bone Marrow Biopsies

Bone marrow biopsies will be performed at screening only for CR/sCR patients. Biopsy performed within 30 days prior to signing the ICF will be acceptable and no new biopsy will be required. A second bone marrow biopsy will be performed at Visit 13 (Day 100 Post Transplantation) if biochemical tests do not show evidence of relapse. Further unscheduled assessment may be performed upon Investigator's discretion.

5.6 PK Assessments

In Part 1, blood samples for PK analysis of BL-8040 or its metabolites will be collected on Day 4 and Day 6 before and at 0.25, 0.5, 1, 2, 4, 8 and 12 hours after BL-8040 administration. Samples should be collected before the first apheresis on Days 5 and 7 (and before G-CSF injection).

The time windows for PK collections in Part 1 are described in detail in the study Laboratory Manual.

In Part 2, blood samples for PK analysis of BL-8040 or its metabolites will be collected on Day 4 at pre-dose of BL-8040 and at 40-60 minutes, 2-6 hours and 10-12 hours post BL-8040 administration. The 12 ±2 hours PK sample should be collected on Day 5 before the apheresis (before G-CSF injection). The same sampling scheme will be followed for Day 6, if applicable.

The exact sampling time relative to BL-8040 administration will be recorded in the CRF.

5.7 Blood Sampling and Processing

Samples will be collected for safety and efficacy analysis, ADA titers, complement and mast cell activation and PK at the time-points indicated in Appendix A.

Instructions for the collection, processing, storage and shipment of samples are detailed in the Laboratory Manual provided by the Sponsor.

5.8 PHARMACOECONOMIC ASSESSMENTS

Resources Use and Cost Elements Data will be collected for inpatient subjects' only during Visit 1 to Visit 13.

6. INVESTIGATIONAL PRODUCT

6.1 IDENTITY OF INVESTIGATIONAL PRODUCT

BL-8040

BL-8040 drug product is formulated as a sterile and non-pyrogenic lyophilized powder in a vial containing 73 mg BL-8040 free base peptide, on dry basis. BL-8040 should be administrated SC following reconstitution with 2 mL 0.45% Sodium Chloride for Injection (Half Normal Saline).

BL-8040 is manufactured in compliance with cGMP by BioConnection (Organon, Kloosterstraat 9, 5349 AB, Oss, Netherlands).

Placebo

BL-8040 Placebo is formulated as a sterile and non-pyrogenic lyophilized powder in a vial containing 50 mg of mannitol. BL-8040 Placebo should be administrated SC following reconstitution with 2 mL 0.45% Sodium Chloride for Injection (Half Normal Saline).

BL-8040 Placebo is manufactured in compliance with cGMP by BioConnection (Organon, Kloosterstraat 9, 5349 AB, Oss, Netherlands).

Packaging and labeling of BL-8040 Drug Product and Placebo will be performed by Fisher Clinical Services (Steinbühlweg 69, 4123 Allschwil, Switzerland).

6.2 STUDY DRUG ADMINISTRATION AND DOSAGE

6.2.1 BL-8040 or Placebo

Patients will receive the first dose of BL-8040 (1.25 mg/kg) or placebo in the evening of the fourth day of G-CSF mobilization, 12 hours ± 2 hours prior to the start of apheresis. A second dose of BL-8040 or placebo may be administered on the evening of Day 6, in patients in whom the collection goal could not be achieved after the second apheresis session on the morning of Day-6.

During Part 1, only BL-8040 will be administered on top of G-CSF.

<u>Pre-Dose medication and general injection instructions:</u>

Premedication with an antihistamine is required for all patients within one hour prior to study drug administration. The time of administration of premedication is to be recorded on site documentation and in the CRF. The preferred route for administration of premedication is IV.

- The recommended premedication regimen is: H1 blocker (e.g diphenhydramine, Promethazine, etc) + H2 blocker (e.g. famotidine, ranitidine, etc) + leukotriene inhibitor (e.g. montelukast). This triple-drug combination is required prior to the first dose of BL-8040

- Analgesic medication may be added to the premedication regimen at the discretion of the treating physician.
- BL-8040 is to be administered at a dose of 1.25 mg/kg by slow subcutaneous injection of at least 2 minutes per syringe. If the volume to be administered exceeds 2 mL then the injection should be divided into two or more syringes to be administered at different injection locations.
- Transient hypotension was witnessed in several cases following the initial treatment with BL-8040. Therefore, caution should be taken with the use of negative chronotropic drugs such as beta blockers. When appropriate, beta blocker should be replaced with non-negative chronotropic drugs.
- All patients are to remain under surveillance for 2 hours after the first administration of BL-8040. Patients receiving an additional dose of BL-8040, should remain under observation for 1 hour after the subsequent administration of BL-8040.
- BL-8040 should only be administered when personnel and therapies are immediately available for the treatment of anaphylaxis and other hypersensitivity reactions, should they occur.

Site of Injection:

The BL-8040 or placebo injections should be given in different areas of the body in case of a divided dose or if a second dose is needed. If the dose volume after reconstitution is higher than 2 mL, injections should be divided in order to maintain injection volume under 2 mL; at the discretion of the Investigator, a single dose administration may be divided and injected into more than one injection site. The same instructions are applicable for the randomization period of the study.

Doses can be injected SC in the arms, legs or abdomen according to the Investigator discretion. Subjects should stay ~1 hour after injection to follow up any possible adverse event.

Dosing calculation:

The patient’s actual body weight, collected at Baseline, will be used to calculate the dosage of BL-8040 administered.

The volume of study drug to be administered will be calculated as follows:

The formula to calculate volume of injection (mL) is:

$$\frac{\text{Patient weight (kg) x drug dose (1.25 mg/kg)}}{\text{36.5 mg/mL}}$$

Results should be rounded to one decimal place. For example: For a patient that weighs 70 kg, the dose calculation is as follows:

(70 kg ×1.25 mg/kg)/ (36.5 mg/mL) = 2.39 mL. Round one decimal place. The volume of BL-8040 to inject is 2.4 mL, divided into two injections.

Table 2 shows the volumes to be injected for different patient weights.

Table 2: Injected Volume based on Patient's Weight

Subject's Weight (kg)	Volume to Inject (mL)
40-42	1.4
43-45	1.5
46-48	1.6
49-51	1.7
52-54	1.8
55-56	1.9
57-59	2.0
60-62	2.1
63-65	2.2
66-68	2.3
69-71	2.4
72-74	2.5
75-77	2.6
78-80	2.7
81-83	2.8
84-86	2.9
87-89	3.0
90-91	3.1
92-94	3.2
95-97	3.3
98-100	3.4
101-103	3.5
104-106	3.6
107-109	3.7
110	3.8

6.2.2 G-CSF

During the G-CSF administration period, each patient will receive ~ 10 µg/kg (maximum of 15 µg/kg) G-CSF in the morning daily for up to a total of 8 days, but a minimum of 5 days, depending on the results of each apheresis. The dose of G-CSF administered will be based on the patient's actual body weight assessed at Baseline. In case dose should be rounded to vial volume, the final dose will be administered according to institution SoC.

G-CSF will be administered as a SC injection. Administration of G-CSF will be documented in the CRF. G-CSF may be given at the clinic, by a visiting nurse, or by patient self-administration at home per patient/institutional preference and local practice pattern.

Following transplantation, G-CSF (~5 µg/kg daily) may be started according to local SoC and may continue until the ANC is $\geq 0.5 \times 10^9$/L for 3 days or $\geq 1.0 \times 10^9$/l for 1 day. Administration of G-CSF will be documented in the CRF.

The number of units of G-CSF injected will be recorded.

6.3 Manufacturing of Study Medication

6.3.1 BL-8040

BL-8040 drug substance (motixafortide (INN), formerly called 4F-benzoyl-TN14003 or BKT140) is a synthetic cyclic peptide. It is a white to off-white powder, freely soluble in water and in 0.45% Sodium Chloride (half normal saline).

BL-8040 drug product is formulated as a sterile and non-pyrogenic lyophilized powder in a vial containing 73 mg BL-8040 free base peptide (on dry basis).

BL-8040 Placebo is formulated as a sterile and non-pyrogenic lyophilized powder in a vial containing 50 mg of mannitol.

Both BL-8040 drug product and its matching placebo are manufactured in accordance with (cGMP) requirements by BioConnection (Organon Kloosterstraat 6, 5349 AB, Oss, Netherlands).

Reconstitution and administration instructions will be provided in a separate study manual.

6.3.2 G-CSF

G-CSF will be sourced from a commercial supplier.

6.4 Packaging and Labeling of Study Medication

Packaging and labeling of BL-8040 and Placebo will be performed by:

Fisher Clinical Services
Steinbühlweg 69
4123 Allschwil
Switzerland

6.5 Distribution and Shipment of Study Medication

The investigational medicinal product will be packed and shipped in appropriate boxes. If, upon arrival at the clinical investigation site, study drug supplies appear to be damaged, the study monitor should be contacted immediately.

Each shipment of study drug supplies for the study will be accompanied by a shipment form describing the contents of the shipment, product certificate of analysis, acknowledgement of receipt and other appropriate documentation. The shipment form will assist in maintaining current and accurate inventory records. The study staff will confirm the receipt of clinical supply by IWRS system.

All study supplies should arrive at the Pharmacy/Investigational site in sufficient quantity and in time to enable dosing as scheduled. The Sponsor or its representative must notify the Principal Investigator's designee prior to dispatch of drug supplies, with the anticipated date of their arrival.

6.6 STORAGE, DISPENSING AND RETURN OF THE INVESTIGATIONAL MEDICINAL PRODUCT

6.6.1 BL-8040 or Placebo

Vials of BL-8040 for injection or its placebo should be stored in the refrigerator (2-8°C) in its original packaging, protected from light.

Records should be kept by the Investigator or designee as to how much study drug was dispensed to each subject. The study monitors must periodically check the study drug supplied to ensure expiry date was not reached, sufficient amount of study drug is available, that drug accountability is being performed at each visit, and drug accountability logs are maintained.

All investigational products must be kept in a secure area with access to the study drug limited to designated study personnel.

Only trained personnel under the supervision of either the Investigator or the local pharmacist are authorized to dispense and administer study drug to participating subjects. Further details and instructions will be provided in the Pharmacy Manual.

6.6.2 G-CSF (Filgrastim)

Institutional guidelines for filgrastim will be followed for storage and dispensation.

6.7 ACCOUNTABILITY AND COMPLIANCE OF INVESTIGATIONAL MEDICINAL PRODUCT

Each delivery must be acknowledged by the hospital pharmacist (or authorized study team member responsible for the investigational medicinal product). Accurate, complete and timely documentation of study drug distribution will be maintained by the pharmacy and the study staff of the investigational site, which may include confirmation of receipts of clinical supply, drug accountability logs and other forms.

The medical center pharmacist (or authorized study team member responsible for the investigational medicinal product) is responsible for ensuring the supervision of the storage and allocation of these supplies, which will be forwarded to the Investigator at the appropriate time before administration. The Investigator may dispense investigational drug only to subjects enrolled in the study.

Drug accountability records must be maintained by the clinical investigation site at all times. At the last study visit, all used and unused investigational drug will be collected and drug accountability performed by the study staff. The study monitor will check these regularly during monitoring visits.

The subject number, the date, batch number/kit number and quantity of study drug used by the subject will be checked for correctness and recorded on the appropriate accountability forms. Unused drug supplies will be returned to the Sponsor. At the end of the study, all clinical supplies and the corresponding accountability forms must be returned to the Sponsor, the study monitor, or designee for reconciliation or destruction. A photocopy of these records must be kept at the clinical investigation site.

The inventory will be made available to the study monitor, who will verify accountability and verify dose during the course of the study.

Study drug orders, records of study drug receipts, dispensing records and inventory forms located at the site will be examined and reconciled by the study monitor periodically during and at the end of the study.

6.8 Concomitant Therapy

If there is a clinical indication for any medication that is not part of the local standard of care and is specifically prohibited during the trial this should be discussed with the Sponsor's Medical Monitor. The final decision on any supportive therapy or vaccination rests with the Investigator and/or the subject's primary physician. However, the decision to continue the subject's trial therapy requires mutual agreement of the Investigator, the Sponsor, and the subject.

The following washout periods prior to the first dose of G-CSF for mobilization are required for the following agents/medications.

- Dexamethasone: 7 days
- Thalidomide: 7 days
- Lenalidomide: 7 days
- Pamolidomide: 7 days
- Bortezomib: 7 days
- Ixazomib :7 days
- Carfilzomib: 7 days
- G-CSF: 14 days
- GM-CSF or Neulasta®: 21 days
- Combination/multi-agent cyto-reductive therapy: 7 days
- Erythropoietin or erythrocyte stimulating agents: 30 days
- Eltrombopag, romiplostim or platelet stimulating agents: 30 days
- BCNU: 42 days/6 weeks
- Daratumumab: 28 days

There are no restrictions against any medications except post-transplantation chemotherapy for maintenance within the 60-days post-transplantation (unless patient is in documented relapse).

6.8.1 Acceptable Concomitant Medications

All treatments that the Investigator considers necessary for a subject's welfare may be administered at the discretion of the Investigator in keeping with the community standards of medical care. If changes occur during the trial period, documentation of drug dosage, frequency, route, and date should also be included in the CRF.

All medications and/or nutritional supplements/therapies taken by patients within 12 months prior to screening and for the duration of study participation will be recorded in the patients' records and transcribed into the CRF as follows: documentation of concomitant medications will begin at screening. Subsequently, patients will be monitored for concomitant medications from the time of the first dose of G-CSF for mobilization until day of transplantation (Visit 10) inclusive. Concomitant medications taken during follow-up visits will be recorded only if myeloma drugs, G-CSF, erythropoietin, alternative stimulating agents

and/or transfusions were also given in that month prior to the visit, to identify any possible medications that may be associated with bone marrow suppression, graft dysfunction or relapse.

Myeloma-related medication, G-CSF, erythropoietin, alternative stimulating agents and/or transfusions should be reported from the screening and up to 100 days post transplantation.

Pre-Dose medication and general injection instructions:

Premedication with an antihistamine is required for all patients, within one hour prior to study drug administration. The time of administration of premedication is to be recorded on site documentation and in the CRF. The preferred route for administration of premedication is IV.

- The recommended premedication regimen is: H1 blocker (e.g diphenhydramine, Promethazine, etc) + H2 blocker (e.g. famotidine, ranitidine, etc) + leukotriene inhibitor (e.g. montelukast). This triple-drug combination is required prior to the first dose of BL-8040
- Analgesic medication may be added to the premedication regimen at the discretion of the treating physician.
- BL-8040 is to be administered at a dose of 1.25 mg/kg by slow subcutaneous injection of at least 2 minutes per syringe. If the volume to be administered exceeds 2 mL then the injection should be divided into two or more syringes to be administered at different injection locations.
- Transient hypotension was witnessed in several cases following the initial treatment with BL-8040. Therefore, caution should be taken with the use of negative chronotropic drugs such as beta blockers. When appropriate, beta blocker should be replaced with non-negative chronotropic drugs.
- All patients are to remain under surveillance for 2 hours after the first administration of BL-8040. Patients receiving an additional dose of BL-8040, should remain under observation for 1 hour after the subsequent administration of BL-8040.
- BL-8040 should only be administered when personnel and therapies are immediately available for the treatment of anaphylaxis and other hypersensitivity reactions, should they occur.

6.8.2 Prohibited Concomitant Medications

Subjects are prohibited from receiving the following therapies during the screening period and Treatment Phase of this trial:

- Those listed previously in the exclusion criteria
- Antineoplastic systemic chemotherapy or biological therapy
- Chemotherapy not specified in this protocol
- Investigational agents other than BL-8040
- Radiation therapy: Radiation therapy to a solitary symptomatic lesion may be considered on an exceptional case by case basis after consultation with the Sponsor.
- Maintenance treatment with lenalidomide, bortezomib, pomalidomide, thalidomide, carfilzomib, etc. within 60 days post-transplantation.

If there is a clinical indication for any medication specifically prohibited during the trial, approval should be discussed with the Sponsor Medical Monitor. Subjects may receive other medications that the Investigator deems to be medically necessary. There are no prohibited therapies after the 60 days post-transplantation during the Post-Treatment Follow-up Phase.

7. SAFETY AND PHARMACOVIGILANCE

7.1 ADVERSE EVENT (AE)

An AE is defined in ICH E6 as "any untoward medical occurrence in a patient or clinical investigation subject administered a pharmaceutical product and which does not necessarily have a causal relationship with this treatment. An AE can therefore be any unfavorable and unintended sign (including an abnormal laboratory finding), symptom or disease temporally associated with the use of a medicinal (investigational) product, whether or not related to the medicinal (investigational) product."

An abnormal result of diagnostic procedures, including abnormal laboratory findings, will be considered an AE if it fulfills one or more of the following:

- Results in subject's withdrawal by the Investigator
- Is associated with an SAE
- Is associated with clinical signs or symptoms
- Is considered by the physician to be of clinical significance
- A new condition or the worsening of a pre-existing condition will be considered an AE.

AEs do not include the following:

- Post-transplantation neutropenia or thrombocytopenia
- Medical/surgical procedures are not AEs (e.g., surgery, endoscopy, tooth extraction, transfusion). The condition that leads to the procedure is an AE if the procedure was not planned at screening visit.
- Overdose of concomitant medication without any signs or symptoms unless the subject is hospitalized for observation.
- Hospitalization for elective surgery planned prior to study (situation where an untoward medical occurrence has not occurred).
- Disease progression

All AEs, whether observed by the Investigator or designee or volunteered by or elicited from the subject, should be recorded individually on an AE CRF page with the following information: The specific event or condition, whether the event was present pre-baseline or not, the dates and times if available (using the 24 hour clock, where midnight is 00:00 and noon is 12:00) of occurrence, duration, severity, relationship to study medication, action taken to study drug, outcome, and whether considered non-serious or serious, drug-related or not.

Once the subject has signed the Informed Consent Form (ICF), AEs will be recorded up to 30 days from the last dose of study drug. The severity of the AE will be assessed by the

investigating physician in accordance with the definitions below. A Serious AE must fulfill the requirements listed in Section 7.2.

AE will be recorded and graded according to the latest version of the NCI-CTCAE (currently version 5.0) (see Table 3) and coded into the database according to the latest version of MedDRA (currently version 20.1).

Table 3: Severity of Adverse Events According to CTCAE (Version 5)

Grade	Description
0	No AE or within normal limits
1	Mild; asymptomatic or mild symptoms; clinical or diagnostic observations only; intervention not indicated.
2	Moderate; minimal, local or noninvasive intervention indicated; limiting age-appropriate instrumental Activities of Daily Living (ADL)
3	Severe or medically significant but not immediately life-threatening; hospitalization or prolongation of hospitalization indicated; disabling; limiting self-care ADL
4	Life-threatening consequences; urgent intervention indicated
5	Death related to AE

A semi-colon indicates 'or' within the description of the grade.
A single dash (-) indicates a grade is not available

The following definitions should be used for toxicities/AEs that are not defined in the CTCAE:

- Mild (Grade 1): The AE is noticeable to the subject but does not interfere with routine activity, no medical intervention is required.
- Moderate (Grade 2): The AE interferes with routine activity but responds to symptomatic therapy or rest.
- Severe (Grade 3): The AE significantly limits the subject's ability to perform routine activities despite symptomatic therapy.
- Life-threatening (Grade 4): The subject is at immediate risk of death.

The Investigator will document his opinion of the relationship of the AE to treatment with investigational product using the criteria outlined in Table 2.

Outcome to Date are classified as follows:

- Recovered: The subject has fully recovered from the AE with no residual effects observable
- Recovered with sequelae: The subject has recovered from the AE with residual effects observable
- Improved: the subject status improved but has not been fully recovered
- Ongoing: AE is not recovered
- Fatal
- Unknown

AEs will be coded by Data Management using the latest version of MedDRA (currently version 20.1) AE dictionary.

All AEs, serious and not serious, will be recorded on the AE Case Report Form, and if relevant, the Concomitant Medications Record in the CRF will be updated. Severity and relationship to study drug will be assessed by the Investigator as described in Table 3. Particular attention should be made to ensure no discrepancies between the AE and the SAE form (i.e. outcome, severity, relationship must be consistent).

Treatment-emergent AEs (TEAEs) are defined as AEs observed after 1st dose of study drug.

Table 4: Relationship of Adverse Event to Treatment

Term	Definition	Clarification
No reasonable possibility (not related)	This category applies to adverse events which, after careful consideration, are clearly due to extraneous causes (disease, environment, etc.) or to adverse events, which, after careful medical consideration at the time they are evaluated, are judged to be unrelated to the study drug.	The relationship of an adverse event may be considered “no reasonable possibility” if it is clearly due to extraneous causes or if at least 2 of the following apply:<br>• It does not follow a reasonable temporal sequence from the administration of the test drug.<br>• It could readily have been produced by the patient’s clinical state, environmental or toxic factors, or other modes of therapy administered to the patient.<br>• It does not follow a known pattern of response to the test drug.<br>• It does not reappear or worsen when the drug is re-administered.
Reasonable possibility (related)	This category applies to adverse events for which, after careful medical consideration at the time they are evaluated, a connection with the test drug administration cannot be ruled out with certainty nor felt with a high degree of certainty to be related to the study drug.	The relationship of an adverse event may be considered “reasonable possibility” if at least 2 of the following apply:<br>• It follows a reasonable temporal sequence from administration of the drug.<br>• It cannot be reasonably explained by the known characteristics of the patient’s clinical state, environmental or toxic factors, or other modes of therapy administered to the patient.<br>• It disappears or decreases on cessation or reduction in dose. There are important exceptions when an adverse event does not disappear upon discontinuation of the drug, yet drug-relatedness clearly exists.<br>• It follows a known pattern of response to the test drug.

All resource use in the management of an AE will be collected.

7.2 Serious Adverse Events (SAEs)

An SAE is any AE occurring at any dose that suggests a significant hazard or side effect, regardless of the Investigator or Sponsor's opinion on the relationship to the investigational medicinal product and that results in, but may not be limited to, any of the following outcomes:

- Death (regardless of the cause)
- A life-threatening experience
- In-patient hospitalization or prolongation of existing hospitalization (any in-patient hospital admission that includes a minimum of an overnight stay in a health care facility)

- A persistent or significant disability/incapacity
- A congenital anomaly or birth defect
- Important medical events that may not result in death, be life-threatening, or require hospitalization may be serious when, based upon appropriate medical judgment, they may jeopardize the subject and may require medical or surgical intervention to prevent one of the outcomes listed above.

In-patient hospitalization or prolongation of existing hospitalization means that hospital in-patient admission and/or prolongation of hospital stay were required for treatment of AE, or that they occurred as a consequence of the event.

Hospitalization for elective treatment of a pre-study condition (pre-baseline) that did not worsen while on study and optional hospitalizations not associated with a clinical AE (e.g. elective cosmetic surgery) are not considered SAEs.

Significant medical events are those which may not be immediately life-threatening but may jeopardize the subject and may require intervention to prevent one of the other serious outcomes listed above. Examples of such events are intensive treatment in an emergency room or at home for allergic bronchospasm; blood dyscrasias or convulsions that do not result in hospitalization; such an AE will normally be considered serious by this criterion.

A **life-threatening** adverse drug experience is any AE that places the subject, in the view of the Investigator, at immediate risk of death from the reaction as it occurred, i.e., it does not include a reaction that, had it occurred in a more severe form, might have caused death.

Any newly emergent SAEs, after treatment is discontinued or the subject has completed the study and is considered to be related to the study drug or study participation, should be recorded and reported immediately to Sponsor or delegate.

All resource use in the management of an SAE will be collected.

7.3 Definition of an Unexpected Adverse Event

An **unexpected** adverse drug event is any AE, the specificity or severity of which is not consistent with information in the current Investigator's Brochure for an unapproved investigational product.

Suspected Unexpected Serious Adverse Reaction (SUSAR) is a SAE assessed as unexpected by the Sponsor and that is judged by either the reporting Investigator or the Sponsor to have a reasonable causal relationship to the investigational medicinal product.

7.4 Exceptions in the Reporting of SAE

According to EU and FDA detailed guidance on the collection, verification and presentation of adverse reaction reports arising from clinical trials on medicinal products for human use, regarding clinical trials in high morbidity or mortality diseases, it is acceptable to define some exceptions in the immediate reporting of specific SAEs. Refer to EU guidance ENTR/CT3, 5.1.9 and FDA guidance "Safety Reporting Requirements for INDs and BA/BE Studies" (Dec 2012).

High dose chemotherapy is a recognized condition with a relatively high morbidity and/or high mortality. Under these circumstances, it seems appropriate that the SAEs, clearly related to the high dose chemotherapy could be an exception for an immediate systematic reporting.

These AEs will be thoroughly handled and followed up through the CRF (AE form) and will be reviewed monthly by the medical safety officer and could be re-qualified for reporting if necessary.

Each event must be carefully analyzed by the Investigator's designee to decide whether the SAE could be considered as an exception or must be immediately reported.

7.5 NOTIFICATION OF SERIOUS ADVERSE EVENT (SAE)

Initial notification of SAEs

An Initial SAE report form must be completed and sent to Pharmacovigilance within 24 hours of the Investigator's knowledge of the event. **Any fatal or life-threatening event should be reported immediately, by phone, CRF, fax and/or email.** Reporting SAEs to regulatory authorities and/or IRBs will comply with local regulations.

**Medical Monitor**	**Pharmacovigilance Manager**
Hemda Chen	Tzipi Lustig

Phone: +972 8 6429100

Email: safety8040@biolinerx.com

Fax: +972 8 6429137

Minimum Criteria for a Valid Initial SAE Case:

For regulatory purposes, initial SAE reports should be submitted to the Sponsor's Medical Monitor or designee immediately and should include:

1. A suspected study drug (whether or not it is suspected to be related to one or both study drugs)
2. An identifiable subject (e.g. study subject code number)
3. An AE with the Investigator's assessment of seriousness
4. An identifiable reporting source (Investigator contact details)

The Initial SAE report will be followed within 24 hours by a completed SAE report including a sufficiently-detailed narrative to allow for a medical assessment of the case, as well as copies of hospital case reports, results of applicable diagnostic tests, laboratory results, biopsy results, autopsy reports and other documents when requested and applicable.

The Investigator must be prepared to supply the Sponsor with the following information:

- Investigator Name and Center Number
- Subject Number
- Subject Initials
- Subject Demographics
- Clinical Event
- Description
- Date of onset

- Severity
- Treatment (including hospitalization)
- Relationship to study drug (causality)
- Action taken regarding study drug
- If the AE was Fatal or Life-threatening
- Cause of death (whether or not the death was related to study drug).
- Autopsy findings (if available).

Once sent, the SAE form and accompanying documentation should be placed in the SAE section of the Investigator's site file.

Follow-up of SAEs:

Follow-up of all SAEs that occur during the study will continue until their satisfactory resolution or stabilization. In outstanding cases, it may be defined as "ongoing without further follow-up" if mutually agreed by the Investigator and Sponsor.

A Follow-up SAE Report Form must be completed by the site (marked as "Follow-up report") and sent to the Pharmacovigilance department within a reasonable timeframe (an SAE Follow-up report is required whether or not there is any additional information to the initial report).

The contact information for Follow-up SAE reporting is the same as for initial SAE reports (see above section).

As for the initial SAE report, once sent, the Follow-up SAE report and accompanying documentation should be placed in the SAE section of the Investigator's site file.

7.6 TIMEFRAME FOR REPORTING REQUIRED EVENTS

For the time-period beginning at the signature of the consent form and ending at Baseline, any SAE that occurs to any subject, and any follow-up to an SAE, including death due to any cause, must be reported within 24 hours to the Sponsor if it causes the subject to be excluded from the trial, or is the result of a protocol-specified intervention, including but not limited to, washout or discontinuation of usual therapy, diet or a study procedure.

For the time period beginning at Screening and up to 30 days after the last dose of study drug, any SAE, or follow-up to an SAE, including death due to any cause, whether or not related to the Sponsor's product, must be reported within 24 hours to the Sponsor either by electronic media or paper.

Additionally, any SAE considered by an Investigator who is a qualified physician to be related to the Sponsor's product that is brought to the attention of the Investigator at any time and up to 30 days following the last dose of study drug must be reported immediately to the Sponsor.

All subjects with SAEs must be followed up for outcomes.

8. STATISTICAL METHODOLOGY

8.1 General

This Phase III study was designed to meet two sequential goals. In Part 1 of the study, a total of up to 30 subjects will be enrolled to an open-labeled treatment with G-CSF followed by BL-8040 1.25 mg/kg with the aim of ensuring that 1.25 mg/kg is the optimal BL-8040 dose. This part will be conducted in 3 sequential steps of 10 subjects each and the accumulated data at each step will be reviewed by an independent DMC.

Following the successful completion of Part 1, a total of 177 subjects will be randomized into Part 2 of the study which will employ a randomized, double-blinded, placebo-controlled design to assess the safety, tolerability and efficacy of BL-8040 + G-CSF as compared to Placebo + G-CSF.

One interim analysis for potential early termination of new subject recruitment into the study will be conducted at the time when a total of 65% of the planned sample size will be randomized and have completed the stem cell mobilization procedure.

8.2 Randomization

Following the successful completion of Part 1, subjects will be randomized using a 2:1 ratio to receive BL-8040 + G-CSF or Placebo + G-CSF, respectively. Randomization will use permuted blocks stratifying subjects by remission status (CR or PR) and baseline platelet count ($<200 \times 10^9$/L or $\geq 200 \times 10^9$/L).

8.3 Interim Analysis, Type-I Error and Multiplicity Adjustment

The overall alpha level for this study is 0.05 using 2-tailed tests. All significance testing for this study will use two-tailed tests.

One interim analysis for potential early termination of new subject recruitment into the study will be conducted at the time when a total of 65% of the planned sample size will be randomized and have completed the stem cell mobilization procedure.

For the interim analysis, treatment effect will be considered statistically significant if the p-value will be 0.0108678 or less. As the interim analysis will be conducted only for potential early termination of new subject recruitment into the study, the final analysis of primary and secondary endpoints, which will be conducted at the Core Study Termination Visit (Visit 13) that will occur at Day 100 following transplantation (or early terminated before), will require a p-value of 0.0466256. These p-values represent the use of the Lan-DeMets' correction to type-I error.

Furthermore, one (1) primary endpoint and 2 secondary endpoints are pre-defined for this study. Hence, there will be a total of 3 comparisons for the primary and secondary endpoints altogether. The hierarchical method for multiple endpoint testing for the secondary endpoints will utilize the gate keeping approach at alpha=0.0466256, ensuring that the overall experiment-wise type-I error of 5% is preserved.

The hierarchical order of primary and secondary endpoints testing will be as outlined below:

1. Primary endpoint: Proportion of subjects mobilizing ≥6.0 x 10^6 CD34+ cells/kg with up to 2 apheresis sessions in preparation for auto-HCT after single administration of BL-8040 + G-CSF or placebo + G-CSF.
2. Proportion of subjects who collect ≥2.0 x 10^6 CD34+ cells/kg in 1 apheresis session.
3. Proportion of subjects who collect ≥6.0 x 10^6 CD34+ cells/kg in 1 apheresis session.

8.4 SAMPLE SIZE JUSTIFICATION

Altogether, a total of up to 207 subjects will be enrolled into the study, as detailed hereafter.

8.4.1 Sample Size Justification for Part 1 (Open labeled period for ascertaining the BL-8040 dose optimization)

To ascertain the dose of BL-8040, a total of up to 30 subjects will be enrolled to Part 1 of the study. The population size of Part 1 was determined based on the following assumptions:

- The endpoint to be used in order to ascertain the BL-8040 dose will be the proportion (%) of subjects mobilizing ≥6.0 x 10^6 CD34+ cells/kg with up to 2 apheresis sessions in preparation for auto-HCT after G-CSF + single administration of BL-8040.
- It is assumed that the proportion (%) of subjects mobilizing ≥6.0 x 10^6 CD34+ cells/kg with up to 2 apheresis sessions due to treatment with BL-8040 + G-CSF will be 70% or more.
- Based on DiPersio et al[20], it is assumed that the null reference hypothesis to be overcome is 35.3%.

Using SAS® PROC POWER for one binomial proportion at a two-sided alpha level of 5%, we determined that a total of 24 subjects will provide 95% power to demonstrate superiority over the reference value of 35.3%. To account for potential dropouts, the Part 1 population size is further increased to a total of 30 subjects.

8.4.2 Sample Size Justification for Part 2 (Randomized, Placebo-Controlled Part)

The power and the derived sample size for Part 2 of the study were calculated based on the following assumptions:

- The primary study endpoint is the proportion (%) of subjects mobilizing ≥6.0 x 10^6 CD34+ cells/kg with up to 2 apheresis sessions in preparation for auto-HCT after G-CSF + single administration of BL-8040 or Placebo + G-CSF.
- Subjects will be randomized to treatment with BL-8040 + G-CSF or to Placebo + G-CSF using a 2:1 randomization ratio, respectively.
- It is assumed that success rates for mobilizing ≥6 x 10^6 cells/kg in MM subjects following treatment with Placebo + G-CSF will be 35%.
- It is also expected that treatment with BL-8040 will result in a success rate of 70%, in line with results obtained for subjects treated with Mozobil + G-CSF. However, as a worst-case scenario, it is assumed that 20% of subjects assigned to the BL-8040 + G-CSF group would not get their assigned treatment and therefore, using the intention-to-treat (ITT) principle, this would reduce the BL-8040 success rate to 63%.

- An additional attrition rate of 20% has also been taken into consideration.
- Final analysis will employ an alpha level of 0.0466256.
- SAS® PROC POWER for two proportions was used to determine the sample size.

According to the above underlying assumptions, a total of 177 subjects will provide 89.8% power to demonstrate that treatment with BL-8040 + G-CSF is superior, at a two-sided alpha of 0.0466256, to treatment with Placebo + G-CSF in the study primary endpoint.

The power at interim analysis, assuming an attrition rate of 20% and success rates of 70% and 35% for BL-8040 + G-CSF or Placebo + G-CSF, respectively, is 85.9%.

8.5 Data Analyses Sets

The following data analysis sets are defined for the analyses of the data accumulated during the conduct of Part 2 of the study:

- **Intention-to-Treat Analysis Set (ITT)**: In accordance with the intention-to-treat principle, all randomized participants will be included in the ITT analysis set according to the treatment group to which they were originally randomized. The Intention-to-Treat (ITT) analysis set will serve as the principal analysis set for efficacy assessments.
- **Modified Intention-to-Treat Analysis Set (mITT)**: The mITT analyses set will consist of all randomized subjects who were treated with the study drug and had at least one post-Baseline efficacy assessment according to the treatment group to which they were originally randomized.
- **Per Protocol Analysis Set (PP):** The per-protocol analysis set (PP) will consist of all randomized subjects who were treated with study drug according to protocol guidelines without any major protocol violations. Analyses will be conducted according to treatment actually administered.
- **Safety Analysis Set (ST):** The safety analysis set (ST) will consist of all subjects who have been randomized and received at least one dose of study\drug according to treatment actually administered. The safety analysis set (ST) will serve as the principal analysis set for safety assessments.

In addition, the following data analysis set is defined for the analyses of the adverse event data accumulated during the conduct of both Part 1 and Part 2 of the study:

- **Study Part 1 and Part 2 Analysis Set (P1P2)**: The P1P2 analyses set will consist of all subjects who were treated with the study drug during Part 1 and Part 2 of the study and had at least one post-Baseline assessment according to the treatment actually administered.

8.6 Sequence of Planned Analyses

Study enrollment may be terminated early in the case that interim analysis will provide statistically significant ($p<0.0108678$) evidence favoring treatment with BL-8040 + G-CSF. Otherwise, study will continue to randomize up to 177 subjects.

The sequence of the planned statistical analyses for this study is described below in the planned chronological order:

- Initially, the interim analysis will be conducted at the time when a total of 65% of the planned study population will be randomized and have completed the stem cell mobilization procedure. The results obtained for this analysis will be used only to determine if study enrollment is to be halted.
- Thereafter, the core set of the study accumulated data will be made available for statistical analyses on the day that the last study randomized subject completes the Core Study Termination Visit (Visit 13), which will occur at his/her Day 100 following transplantation (or early terminated before). At that time the study database will be locked and a formal statistical analysis of the following endpoints will be conducted:
  - ✓ Primary Endpoint: Proportion of subjects mobilizing ≥6.0 x 10^6 CD34+ cells/kg with up to 2 apheresis sessions in preparation for auto-HCT after single administration of BL-8040 + G-CSF or placebo + G-CSF.
  - ✓ Secondary Endpoint: Proportion of subjects who collect ≥2.0 x 10^6 CD34+ cells/kg in 1 apheresis session.
  - ✓ Secondary Endpoint: Proportion of subjects who collect ≥6.0 x 10^6 CD34+ cells/kg in 1 apheresis session.
  - ✓ Additional Secondary Endpoint: Time to neutrophil engraftment, defined as ANC ≥0.5 x 10^9/L for 3 consecutive days or ≥1.0 x 10^9/L for 1 day following the conditioning regimen associated nadir.
  - ✓ Additional Secondary Endpoint: Time to platelet engraftment, defined as the first of 3 consecutive measurements of platelet count ≥20 x 10^9/L without platelet transfusion support for 7 days following the conditioning regimen associated nadir.
  - ✓ Additional Secondary Endpoint: Time to engraftment defined as the time to neutrophils and platelets engraftment whichever comes later.
  - ✓ Additional Secondary Endpoint: Graft durability at 60 days post-transplantation - only in case of patients in whom maintenance therapy will be given around Day 60.
  - ✓ Additional Secondary Endpoint: Graft durability at 100 days post-transplantation.
  - ✓ Economic endpoint: Resource use and cost up until engraftment, 100 days after transplantation.
  - ✓ Exploratory Endpoint: Change from Baseline in peripheral blood CD34+ cell concentration on apheresis Day 1 or Day 1 and 2, if applicable, after BL-8040/placebo single administration.
  - ✓ Exploratory Endpoint: Mobilization of Myeloma cells assessed in the apheresis product after BL-8040/placebo administration.
  - ✓ Exploratory endpoint: To descriptively compare the CD34+ cells collected per kg of actual body weight (ABW) used in this study to the theoretical amount of CD34+ cells collected based on ideal body weight (IBW)

  The results and conclusions obtained from the integrative assessment of these efficacy endpoints, as well as accumulated safety data up to this cut-off date, will be summarized in a Clinical Study Report (CSR) in support of regulatory approval of BL-8040 + G-CSF for the treatment of Multiple Myeloma.

  BioLineRx believes that unblinding the study following the day that the last randomized subject completes the Core Study Termination Visit, which will occur at his/her Day 100

following transplantation, will not compromise study integrity and blinding and thus will not be associated with any bias for the subsequent sequence of the planned analyses due to the following reasons:

- ✓ Study recruitment, randomization and administration of study IP will already be completed at this study stage.
- ✓ Long-term graft durability endpoints are based on objective measures and therefore are not likely to be influenced by treatment allocation.
- ✓ The individual subject treatment assignment and data listings will only be shared with the Sponsor's dedicated taskforce and with regulatory authorities.
- ✓ The publicly disseminated information will be on a by "group basis".
- ✓ The observed summary statistics of efficacy outcomes will not allow determining individual subject assignment.
- ✓ The observed summary statistics of adverse events will not allow determining individual subject assignment.

The third set of study accumulated data will be made available for statistical analyses of the long-term graft durability endpoints, as well as the longer-term safety data, on the day the last randomized subject completes Visit 16, which will occur at 12 months following transplantation. At that time the study database will be locked again, and a formal statistical analysis of the following endpoints will be conducted:

- ✓ Additional Secondary Endpoint: Graft durability at 6 months post-transplantation.
- ✓ Additional Secondary Endpoint: Graft durability at 12 months post-transplantation.
- ✓ Exploratory Endpoint: Proportion of subjects with graft failure post-transplantation.

The results and conclusions obtained from the integrative assessment of these secondary efficacy endpoints, as well as accumulated safety data up to this cut-off date, will be summarized in a supplement to the CSR in further support of regulatory approval of BL-8040 + G-CSF for the treatment of Multiple Myeloma.

- The last set of study accumulated data will be made available for statistical analyses of the long-term exploratory endpoints at 5 years following the randomization of the last study subject. At that time, the entire study will be terminated, the study database will be locked again, and a formal statistical analysis of the following endpoints will be conducted:
  - ✓ Exploratory Endpoint: Overall Survival (OS) during the period of up to 5 years.
  - ✓ Exploratory Endpoint: Relapse-Free Survival (RFS) during the period of up to 5 years.

The results and conclusions obtained from the integrative assessment of these exploratory efficacy endpoints will be summarized in an additional supplement to the CSR in further support of the regulatory approval of BL-8040 + G-CSF for the treatment of Multiple Myeloma.

8.7 Interim Analyses

An interim analysis for the primary endpoint only will be conducted for potential early termination of new subject recruitment into the study as described above.

8.8 EFFICACY ENDPOINTS AND ANALYSES

All efficacy endpoints will be tested for the ITT analysis set controlling for multiplicity as outlined in Section 8.3.

8.8.1 Primary Efficacy Endpoint and Principal Statistical Analysis

The primary study endpoint is the proportion (%) of subjects mobilizing ≥6.0x10^6 CD34+ cells/kg with up to 2 apheresis sessions in preparation for auto-HCT after G-CSF + single administration of BL-8040 or Placebo + G-CSF.

The following rules will be incorporated to derive this endpoint:

- Only subjects randomized to study treatment during Part 2 of the study will be included in the formal efficacy analysis.
- A subject who cumulatively mobilizes ≥6.0 x 10^6 CD34+ cells/kg with up to 2 apheresis sessions will be classified as a “Responder”.
- Other subjects, including randomized subjects who failed to complete the stem cell mobilization procedure, as well as those who did not mobilize 6.0 x 10^6 CD34+ cells/kg after up to 2 apheresis sessions, will be classified as “Non-Responders”.

The principle analysis of this binary endpoint will use the Cochran-Mantel-Haenszel test stratified by remission status (CR or PR) and baseline platelet count (<200 x 10^9/L or ≥200 x 10^9/L).

In addition, the number and proportion of subjects meeting this endpoint will be displayed by treatment group.

8.8.1.1 Sensitivity Analyses for the Principal Analysis of the Primary Endpoint

The robustness of the results of the principal analysis of the primary endpoint will be explored employing the following:

- Primary Sensitivity Analysis Using Logistic Regression:

  The primary sensitivity analysis of this binary end-point will be based on estimating a contrast (BL-8040 + G-CSF vs. Placebo + G-CSF) derived from a baseline-adjusted, Logistic Regression model [SAS® PROC GENMOD with DIST=BIN and LINK=LOGIT] to this binary outcome measure. In addition to the treatment group, the model will include the following covariates: remission status (CR or PR) and baseline platelet count (<200 x 10^9/L or ≥200 x 10^9/L).

  The treatment effect (odds-ratio) of BL-8040 + G-CSF vs. Placebo + G-CSF on the corresponding adjusted proportions will be displayed
- Sensitivity Analysis Accounting for Missing Values:

  The ITT analysis set will be used for the below sensitivity analysis. To evaluate the possible impact of missing values on the principal analysis of the primary endpoint results assuming all missing data are Missing Not At Random (MNAR), a total of additional 4 sensitivity analyses using the primary analysis statistical model, will be conducted:

- The first analysis will consider randomized patients with missing data as "Responders".
- The second analysis will consider randomized patients with missing data as "Non-Responders".
- The third analysis will consider BL-8040+G-CSF randomized patients with missing data as "Non-Responders" and BL-8040+Placebo randomized patients with missing data as "Responders".
- The fourth analysis will consider BL-8040+G-CSF randomized patients with missing data as "Responders" and BL-8040+Placebo randomized patients with missing data as "Non-Responders".

- Other Sensitivity Analyses

  Other sensitivity analyses will be defined in the SAP.

8.8.2 Secondary Efficacy Endpoints and Statistical Analysis

Secondary endpoints will be tested for the ITT analysis set controlling for multiplicity using the hierarchical gate keeping method for multiple endpoints testing for the secondary endpoints. Study secondary endpoints will be tested using the gatekeeping principle as defined above in the following order:

8.8.2.1 Proportion of subjects who collect ≥2.0 x 10^6 CD34+ cells/kg in 1 apheresis session

The following rules will be incorporated to derive this endpoint:

- Only subjects randomized to study treatment during Part 2 of the study will be included in the analysis.
- A subject who collects ≥2.0 x 10^6 CD34+ cells/kg in 1 apheresis session in preparation for autologous hematopoietic cell transplantation (auto-HCT) will be classified as a "Responder".
- Other subjects, including randomized subjects who failed to complete the stem cell mobilization procedure, as well as those who did not mobilize 2.0 x 10^6 CD34+ cells/kg in 1 apheresis session, will be classified as "Non-Responders".

The analysis of this binary endpoint will use the Cochran-Mantel-Haenszel test stratified by remission status (CR or PR) and baseline platelet count ($<200 \times 10^9$/L or $\geq 200 \times 10^9$/L).

Sensitive analysis of this binary endpoint will be based on estimating a contrast (BL-8040 + G-CSF vs. Placebo + G-CSF) derived from a baseline-adjusted, Logistic Regression model [SAS® PROC GENMOD with DIST=BIN and LINK=LOGIT] to this binary outcome measure. In addition to the treatment group, the model will include the following covariates: remission status (CR or PR) and baseline platelet count ($<200 \times 10^9$/L or $\geq 200 \times 10^9$/L).

The treatment effect (odds-ratio) of BL-8040 + G-CSF vs. Placebo + G-CSF on the corresponding adjusted proportions will be displayed.

In addition, the number and proportion of subjects meeting this endpoint will be displayed by treatment group.

8.8.2.2 Proportion of subjects who collect ≥6.0 x 10^6 CD34+ cells/kg in 1 apheresis session

The following rules will be incorporated to derive this endpoint:

- Only subjects randomized to study treatment during Part 2 of the study will be included in the analysis.
- A subject who mobilizes ≥6.0 x 10^6 CD34+ cells/kg in 1 apheresis session in preparation for autologous hematopoietic cell transplantation (auto-HCT) will be classified as a "Responder".
- Other subjects, including randomized subjects who failed to complete the stem cell mobilization procedure, as well as those who did not mobilize 6.0 x 10^6 CD34+ cells/kg in 1 apheresis session, will be classified as "Non-Responders".

The analysis of this binary endpoint will be based on estimating a contrast (BL-8040 + G-CSF vs. Placebo + G-CSF) derived from a baseline-adjusted, Logistic Regression model [SAS® PROC GENMOD with DIST=BIN and LINK=LOGIT] to this binary outcome measure. In addition to the treatment group, the model will include the following covariates: remission status (CR or PR) and baseline platelet count (<200 x 10^9/L or ≥200 x 10^9/L).

The treatment effect (odds-ratio) of BL-8040 + G-CSF vs. Placebo + G-CSF on the corresponding adjusted proportions will be displayed.

In addition, the number and proportion of subjects meeting this endpoint will be displayed by treatment group.

8.8.3 Additional Secondary Efficacy Endpoints and Between Study Groups Descriptive Comparability Assessment

For the comparability between the effects of BL-8040 + G-CSF versus placebo + G-CSF the below endpoints will be assessed in a descriptive manner:

8.8.3.1 Time from transplantation to neutrophil engraftment, defined as ANC ≥0.5 x 10^9/L for 3 consecutive days or ≥1.0 x 10^9/L for 1 day following the conditioning regimen associated nadir

This endpoint will be presented using a Kaplan-Meier plot stratified by study group. Any estimate quoted from the Kaplan-Meier plot will have the estimate and the standard error (SE) of the estimate provided. Greenwood's formula will be used to calculate the SE.

8.8.3.2 Time from transplantation to platelet engraftment, defined as the first of 3 consecutive measurements of platelet count ≥20 x 10^9/L without platelet transfusion support for 7 days following the conditioning regimen associated nadir

This endpoint will be presented using a Kaplan-Meier plot stratified by study group. Any estimate quoted from the Kaplan-Meier plot will have the estimate and the standard error (SE) of the estimate provided. Greenwood's formula will be used to calculate the SE.

8.8.3.3 Time from transplantation to engraftment defined as the time to neutrophils and platelets engraftment whichever comes later.

This endpoint will be presented using a Kaplan-Meier plot stratified by study group. Any estimate quoted from the Kaplan-Meier plot will have the estimate and the standard error (SE) of the estimate provided. Greenwood's formula will be used to calculate the SE.

8.8.3.4 Graft durability at 60 days post-transplantation.

The number and proportion of subjects meeting this endpoint will be provided by treatment group and the odds-ratio of BL-8040 + G-CSF vs. Placebo + G-CSF on the corresponding proportions will be displayed.

8.8.3.5 Graft durability at 100 days post-transplantation.

The number and proportion of subjects meeting this endpoint will be provided by treatment group and the odds-ratio of BL-8040 + G-CSF vs. Placebo + G-CSF on the corresponding proportions will be displayed.

8.8.3.6 Graft durability at 6 months post-transplantation

The number and proportion of subjects meeting this endpoint will be provided by treatment group and the odds-ratio of BL-8040 + G-CSF vs. Placebo + G-CSF on the corresponding proportions will be displayed.

8.8.3.7 Graft durability at 12 months post-transplantation.

The number and proportion of subjects meeting this endpoint will be provided by treatment group and the odds-ratio of BL-8040 + G-CSF vs. Placebo + G-CSF on the corresponding proportions will be displayed.

8.8.3.8 Pharmacoeconomic endpoint

The resource utilization up until engraftment, 100 days after transplantation, multiplied with per country unit costs to obtain an average cost per patient per treatment arm.

8.8.4 Exploratory Endpoints

Analyses of the exploratory endpoints will provide additional insight into the therapeutic effect of BL-8040 + G-CSF as compared to Placebo + G-CSF. These endpoints will be tested for the ITT analysis set using the nominal alpha level of 5% without controlling for multiplicity. Detailed statistical methodology to be used for the analyses of these exploratory endpoints will be provided in a more detailed SAP to be developed while the study is ongoing and prior to locking the database and unblinding. The exploratory endpoints to be analyzed are:

- Change from baseline in peripheral blood CD34+ cells concentration on apheresis Day 1 or Day 1 and Day 2, if applicable, after BL-8040/placebo single administration.
- Proportion of subjects with graft failure post-transplantation.
- Overall Survival (OS) during the period of up to 5 years.
- Relapse-Free Survival (RFS) during the period of up to 5 years.
- Mobilization of Multiple Myeloma cells assessed in the apheresis product.
- CD34+ cells collected per kg of actual body weight (ABW) compared to theoretical amount of cells collected based on ideal body weight (IBW).

8.9 Safety and Tolerability Analyses

Safety analyses will be performed for the Safety Analysis Set (ST) and for the Study Part 1 and Part 2 Analysis Set (P1P2).

8.9.1 Adverse Events

An adverse event (AE) is defined in ICH E6 as "any untoward medical occurrence in a patient or clinical investigation subject administered a pharmaceutical product and which does not necessarily have a causal relationship with this treatment. An AE can therefore be any unfavorable and unintended sign (including an abnormal laboratory finding), symptom or disease temporally associated with the use of a medicinal (investigational) product, whether or not related to the medicinal (investigational) product." The MedDRA dictionary will be used to standardize the terms used by the investigator to describe the Adverse Events (AEs).

Adverse events will be recorded from the time a subject has signed the Informed Consent Form. Adverse events analyses will include only the Treatment Emergent Adverse Events (TEAEs), namely, those events which started at the time of first study IP administration or afterwards. The following analyses are pre-planned for adverse events:

- The incidence (no. of patients) and frequency (no. of events) of most frequent TEAEs (>5%) by Preferred Term (PT).
- The incidence (no. of patients) and frequency (no. of events) of TEAEs broken down by System Organ Class (SOC) and PT.
- The incidence (no. of patients) and frequency (no. of events) of TEAEs broken down by SOC, High Level Term (HLT) and PT.
- The incidence (no. of patients) and frequency (no. of events) of TEAEs by SOC and PT in Subjects Early Discontinued from Study/Treatment.
- The incidence (no. of patients) and frequency (no. of events) of serious TEAEs by SOC and PT.
- The incidence (no. of patients) and frequency (no. of events) of serious TEAEs by SOC, HLT and PT.
- The incidence (no. of patients) and frequency (no. of events) of TEAEs when broken down by severity.
- The incidence (no. of patients) and frequency (no. of events) of TEAEs broken down by relationship to study IP.
- The incidence (no. of patients) and frequency (no. of events) of TEAEs when broken down by action taken with study IP.
- The incidence (no. of patients) and frequency (no. of events) of TEAEs when broken down by event outcome.

The Adverse Events dictionary used to code Investigator's verbatim terms will be provided.

Individual subject listings of all SAEs, treatment-emergent SAEs, TEAEs and non-TEAEs will be provided.

8.9.2 Safety Laboratory Data

Analyses of safety central laboratory data will be performed in the following manner:

- Quantitative laboratory measurements will be categorized with reference to the normal ranges as Low, Normal or High. The incidence (no. of subjects) of abnormal values at

any time post initiation of the 1st study IP administration, calculated for subjects with normal values at Baseline will be provided. Analysis will include, per tested parameter, those subjects with normal baseline and at least one post-treatment measurement. A summary table will display the number and relative percentage of subjects with at least one abnormal value (above upper or below the lower normal range) at any time post-initiation of the 1st study IP administration.

- Quantitative laboratory measurements will be categorized with reference to the normal ranges as Low, Normal or High. Shift analysis of the categorical change from Baseline to each scheduled visit and to the last observed value will be provided.
- Box-Plots of measurements done and figures of mean values ±SEs, as well as descriptive statistics for all laboratory quantitative parameters and changes from Baseline, will be provided by scheduled visits.

8.9.3 Vital Signs

Analyses of vital signs data will be performed in the following manner:

- The more detailed SAP will provide the quantitative criteria used to define the potentially clinically significant (PCS) abnormal vital signs values. Measurements to be used in the analysis are those taken immediately following the first administration of the study IP and onwards. The incidence tables of PCS values as well as the individual subject listing will be provided. The denominator to be used for calculating percentages is the number of subjects with at least one observation post 1st study IP administration.
- Box-Plots of measurements done, figures of mean values ±SEs, as well as descriptive statistics for all parameters and changes from Baseline (derived), will be provided by scheduled visits and treatment groups.

8.9.4 Physical Examinations

Analyses of physical examinations will be performed in the following manner:

- Distribution of number of subjects by body system examined and result assessments made will be presented at Baseline and at Termination Visit.
- Shift analysis of categorical change by body system will also be performed.
- Incidence and listings of individual subject's findings of Abnormal Clinically Significant following 1st study IP administration and onwards will also be provided.

8.9.5 12-Lead ECG

12-lead ECG will be recorded at Screening, Baseline and triplicate ECG on Day 4 prior to dosing and 30-45 min after study drug and on Day 5 prior to apheresis. If a second dose of study drug is provided, another triplicate ECG assessment will be performed on Day 6 pre-dose and 30-45 min post dose[a] and prior to third apheresis. Additional ECGs may be performed at the discretion of the Investigator. Analyses of ECG evaluations will be presented in the report in the following manner:

- Descriptive statistics and Box-Plots of quantitative 12-Lead ECG parameters measured by scheduled timepoints (Pre-Dose/Post-Dose) as well as their changes from Baseline.

[a] Post dose defined as end time of administration

- Distribution of the number of subjects by the interpretation (Normal/Abnormal) at each scheduled timepoint.
- Listing of QTC Interval Fridericia (msec) Measurements > 450 msec AND changes from baseline > 60 msec will be listed along with Baseline value and change from Baseline.

8.9.6 Tolerability Assessment

Tolerability and drop-out assessments will be performed for the ITT analysis set.

Tolerability analysis will be based on the number and percent (%) of subjects who failed to complete the core study period and will be presented by withdrawal reason.

The time to withdrawal will be presented using a Kaplan-Meier Plots. Any estimate quoted from these plots will have the estimate and the standard error (SE) of the estimate provided. Greenwood's formula will be used to calculate the SE.

8.10 PHARMACOKINETIC ASSESSMENTS

Part 1:

Plasma concentrations of BL-8040 or its metabolites in subjects that received BL-8040 will be analyzed by model-independent methods using Phoenix WinNonlin version 6.4 or higher (Pharsight, Inc., Mountain View, CA). Actual sampling times will be used in this analysis. Pharmacokinetic parameters will include Cmax, AUC and $t_{½}$ whenever practical. Individual and mean serum concentrations of BL-8040 versus time data will be tabulated and plotted. In addition, individual and summary statistics (mean, standard deviation, and range) of PK parameters will be tabulated.

Part 2:

Sparse PK samples collected in this Phase III study may be used to estimate population pharmacokinetic parameters of BL-8040 or its metabolites, if possible. In addition, the effect of covariates (such as hepatic and renal function) on the pharmacokinetics of BL-8040 may be examined in the population analysis.

The PK population for analysis will include all patients who have at least one evaluable PK sample post-dose.

Individual plasma concentrations of BL-8040 or its metabolites versus time data will be tabulated and plotted. Estimates of the estimated PK parameters will be tabulated and summarized (mean, standard deviation, and range), if possible.

The pharmacokinetic assessments may be reported in a dedicated analysis report separately from the Clinical Study Report.

8.11 MORE DETAILED STATISTICAL ANALYSIS PLAN (SAP)

A more detailed SAP will be developed while the study is blinded, prior to the conduct of the predefined interim analysis.

8.12 STATISTICAL SOFTWARE

All data listings, summary tables and statistical analyses will be generated using SAS® Version 9.4 or higher (SAS is a registered trademark of the SAS Institute Inc., Cary, NC, USA).

9. ETHICS

9.1 Institutional Review Board (IRB) or Independent Ethics Committee (IEC)

Prior to initiation of the study, the Investigator will submit the study protocol and amendments, IB and amendments, ICF and any other documents that may be provided to the subject or any other documents requested by the IRB/IEC for review and approval.

The names and affiliations of all members of the IRB/IEC must be provided to the PI and BioLineRx. In lieu of this, the IRB/IEC must certify that it has been officially authorized/recognized according to the national legislation.

The IRB/IEC must provide written approval of the study to keep in the Investigator's file. Records of approval of all documents pertaining to this study, including the local Regulatory Authority, should be filed as such. The Investigator will not begin the study until the protocol, ICF and any other document provided to the subject have been approved by the IRB/IEC. The Investigator must agree to make any required progress reports to the IRB/IEC, as well as reports of SAEs, life-threatening conditions or death. The IRB/IEC will also be notified of Part 1 preliminary results.

9.2 Ethical Conduct of the study

All clinical work conducted under this protocol is subject to ICH GCP E6 (R2) guidelines. This includes an inspection by Sponsor or its designee, Health Authority or IRB/IEC representatives at any time. The Investigator must agree to the inspection of study-related records by Health Authority representatives and/or Sponsor or its designee.

The study will be conducted in accordance with Sponsor and/or designee's standards operating procedures and the following guidelines:

- GCP: Guideline for Good Clinical Practice ICH E6(R2)- ICH Harmonized Guideline Integrated Addendum to ICH E6 (R1) (International Conference on Harmonization of Technical Requirements for the Registration of Pharmaceuticals for Human Use), Step 5, 14 June 2017
- Declaration of Helsinki: Seoul, 2008
- US Code of Federal Regulations (Title 21, CFR Part 11, 50, 54, 56 and 312) and/or EU Directives; and/or local country regulations and guidelines.

9.3 Subject Information and Consent

Prior to screening for the study, each subject will be informed in detail about the study drugs to be administered and the nature of the clinical investigation with its risks and discomforts to be expected. The basic elements of informed consent as specified by the FDA (21 CFR 50.25) and ICH-GCP will be followed. The subjects will also be instructed that they are free to withdraw their consent and discontinue their participation in the study at any time without prejudice. Written consent will be obtained from each subject to be involved in the clinical trial or his/her designated power of attorney (POA) by using the IRB/IEC-approved ICF prior to the conduct of any study-related activity. A copy of the ICF will be submitted together with this protocol and must be approved by the IRB/IEC prior to study commencement. Each subject will be given a copy of the written ICF, and each subject's chart will include the signed ICF for study participation. The original subject signed and dated ICFs will be

maintained per ICH record retention requirements. Regulatory authorities may check the existence of the signed ICF in this central study folder if not having performed so during the study.

9.4 Subject Insurance

A product liability to cover against injury and damages arising from the use of investigational products in this project is provided by the Sponsor for the total duration of the study, covering the subjects and Investigators in respect of the risks involved in conducting this study according to this protocol. The insurance policy will be filed in the Investigator's site file or can be made available to the Investigator and to the IRB/IEC upon request.

Subjects will be insured through contract between an insurance company and the Sponsor.

9.5 Informing the General Practitioner

When required by location regulation, the Investigator will inform the subject's primary care physician of his/her participation in the study, by sending a letter to the physician.

9.6 Personal Data Protection

The Sponsor will comply with local regulations and with the principle of subject's right to protection against invasion of privacy. Throughout this trial, all subject data will be identified only by a subject identification number and subject initials and date of birth. The data will be blinded in all data analyses. The subject must be informed and consent to authorized personnel of the Sponsor, such as study monitor, auditor, etc. and relevant health regulatory agencies having direct access to personal medical data to assure a high-quality standard of the study. At the subject's request, medical information may be given to his or her personal physician or other appropriate medical personnel responsible for his or her welfare.

9.7 Data and Safety Monitoring

An independent DMC will be responsible for periodically reviewing safety data collected during the study. Details with regard to DMC activities will be provided with the DMC Charter.

Please refer to the Operations Manual for list of DMC members.

9.8 Protocol Exceptions and Deviations

No prospective waivers will be allowed for patients who do not fulfill the inclusion/exclusion criteria. Deviations from the protocol should be avoided, unless required for the safety of the subject. Major Protocol deviations, and, if possible, the reason for occurrence, will be documented by the study monitor for visit reports and will be included in the final clinical study report. The Investigator must report any protocol deviations to the Sponsor or the Sponsor's designee, should they occur. If required, the Investigator should also report deviations to the IRB/IEC in accordance with local regulations and within a reasonable time.

9.9 Protocol Amendments

Changes to the protocol may be made only by the Sponsor (with or without consultation with the Investigator). All protocol modifications must be submitted to the site IRB/IEC in accordance with local requirements and, if required, to the Regulatory Authority, either as an

amendment or a notification. Approval for amendments must be received before any changes can be implemented, except for changes necessary to eliminate an immediate hazard to trial subjects, or when the changes involve only logistical or administrative aspects of the trial. No approval is required for notifications.

10. QUALITY CONTROL AND QUALITY ASSURANCE

The study will be conducted according to GCP as outlined by ICH Topic E6 (R2) Step 5 guidelines. The CRO's Standard Operating Procedures (SOPs) will be followed to ensure that clinical trials are conducted and data are generated, documented and reported in compliance with the protocol, GCP and applicable regulatory requirements.

10.1 AUDITS AND INSPECTIONS

The study may be audited according to the Sponsor's or its designee's QA inspection program. The purpose of the audit is to determine whether or not the study is being conducted and monitored in compliance with the study protocol and ICH GCP guidelines. Audit visit(s) will be arranged in advance with site personnel at a mutually acceptable time.

The Investigator should understand that source documents for this trial should be made available to appropriately qualified personnel from the Sponsor or its designees or the regulatory authority inspectors, after appropriate notification. The verification of the CRF data must be made by direct inspection of source documents. The auditor may ask to visit the facilities where laboratory samples are collected, where the investigational product is stored and prepared and any other facility used during the study. These audits or inspections may take place at any time, during or after the study, and are based on the national regulations, as well as ICH guidelines.

10.2 STUDY MONITORING

The Study Monitor will be responsible for ensuring adherence to FDA, ICH guidelines and the Sponsor's or Sponsor's designee Standard Operating Procedures. Study Monitors for this trial will be provided by the Sponsor. The monitors will follow the current "Guideline for the Monitoring of Clinical Investigators", supplied by the FDA in compliance with ICH Guidelines.

Experienced independent monitors or monitors from Clinical Research Organizations (CROs) will be trained by the Sponsor in order to standardize the monitoring procedure. These monitors will be trained on ICH GCP Guidelines, the Sponsor's SOPs, study protocol and the study monitoring conventions.

Regular monitoring of study data at each site will be performed in accordance with applicable regulations. Individual sites will be monitored to ensure that enrollment rate, data recording, and protocol adherence are satisfactory. The frequency of monitoring individual sites may fluctuate depending upon enrollment rate and the quantity of data collected and will be documented in the monitoring plan.

These monitoring visits will be performed for the purposes of verifying adherence to the protocol and the completeness and exactness of data entered on the CRF. The study monitor will verify CRF entries by comparing them with the primary source documents (hospital/clinic/office records), which will be made available for this purpose. The monitor will review the maintenance of regulatory documentation and drug accountability. The

monitor will review the progress of the study with the Investigator and other site personnel on a regular basis. Case report form sections may be collected during these visits. At the end of the study, a close-out monitoring visit will be performed. Monitoring visits will be arranged in advance with site personnel at a mutually acceptable time. Sufficient time must be allowed by the site.

Periodically, some or all of the facilities used in the study (e.g., local laboratory, pharmacy) may be reviewed. Monitoring visits will be arranged in advance with site personnel at a mutually acceptable time. Sufficient time must be allowed by the site personnel for the monitor to review CRFs and relevant source documents. The Investigator should be available to answer questions or resolve data clarifications. The Investigator or appointed delegate will receive the study monitor during these on-site visits, cooperate in providing the documents for inspection and respond to enquiries.

The Investigator will ensure that the study participants are aware of and consent to their personal information being scrutinized during the data verification process, as part of study-related monitoring, inspection and/or auditing, by properly authorized persons associated with Sponsor or by domestic and/or foreign regulatory authorities. However, the subject's participation and personal information will be treated as strictly confidential to the extent that the applicable law permits and will not be made publicly available.

Upon completion of the study, the study monitor will arrange for a final review of the study files after which the files should be secured for the appropriate time period.

10.3 QUALITY LABORATORY STANDARDS

Laboratory tests or evaluations described in this protocol will be conducted in accordance with quality laboratory standards as described in the SOPs of the central and local institution laboratories.

Before the study begins, the laboratories to be used in the study will provide a list of the reference ranges for all laboratory tests to be undertaken and details of the method used for quality control. These will be held in the Investigator Site File and the Trial Master File. The methods employed for each assay should be available on request. Any change in the laboratory, its procedures, references, values, etc. during the study must be notified promptly to the Sponsor.

10.4 STUDY DOCUMENTATION

The Investigator must maintain primary source documents supporting CRF data entries. These documents, which are considered "source data", should include documentation of:

- Demographic information.
- Evidence supporting the diagnosis/condition for which the subject is being studied
- General information supporting the subject's participation in the study.
- Medical history and physical findings.
- Hospitalization or Emergency Room records (if applicable).
- Each study visit by date, including any relevant findings/notes by the investigator(s), occurrence (or lack) of adverse events, and changes in medication usage, including the date the study drug was commenced and completed.
- Any additional visits during the study

- Any relevant telephone conversations with the subject regarding the study or possible adverse events.
- Original, signed informed consent forms for study participation.

The investigator must also retain all subject-specific printouts/reports of tests/procedures performed as a requirement of the study. During monitoring visits, the monitor will need to validate data in the CRFs against these source data.

10.4.1 Source Document

The Investigator will permit study-related monitoring, audits by or on behalf of the Sponsor, IRB/IEC review and regulatory inspections providing direct access to source data documents. Source documents are original records in which raw data are first recorded. These may be office/clinic/hospital records, charts, diaries, x-rays, laboratory results, printouts, pharmacy records, care records or completed scales for each study participant. Source documents should be kept in a secure and limited access area. All source documents must be accurate, clear, unambiguous, permanent and capable of being audited. They should be made using a permanent form of recording (ink, typing, printing, optical disc etc.). They should not be obscured by correcting fluid or have temporary attachments (such as removable self-stick notes). Source documents that are computer generated and stored electronically must be printed, signed and dated by the Investigator.

Source data for subjects registered to the study should indicate the date the ICF was signed, participation in a clinical trial with the clinical protocol number and title, treatment number and evidence that inclusion/exclusion criteria have been met.

10.4.2 Recording of Data on Case Report Form (CRF)

The development of the CRF will be the responsibility of the Sponsor or its designee.

All the pertinent data will be recorded on an electronic Case Report Form (eCRF). All eCRFs will be completed in English and will be reviewed by study monitors for accuracy and completeness. The eCRFs should be completed at the time of the subject's visit, with the exception of results of tests performed outside the Investigator's office. The Investigator is responsible for verifying that all data entries in the eCRFs are accurate and correct. The Principal Investigator must sign the completed CRF prior to its submission to the Sponsor.

A representative of the Sponsor or designee will instruct the Investigator and his/her staff prior to the enrollment of the first patient and will train them on recording the findings into the electronic data capture (EDC) system.

<u>(EDC) system on the electronic CRFs (eCRFs)</u>:

After the enrollment of the first patient, a study monitor will periodically monitor the progress of the study by conducting onsite visits. This study monitor will also have the ability to review query statuses remotely, which may warrant more frequent contact with the Investigator and his/her staff. The Investigator will make available to the study monitor the computer that accesses the eCRFs, source documents, signed consent forms and all other study related documents. The Investigator will be responsible for reviewing eCRFs, providing resolution to data queries generated by the study monitor via the EDC system, providing missing or corrected data, and approving all changes performed on his/her data, and endorsing the patient data within the EDC system. This approval method will include

applying an electronic signature, a uniquely assigned username and a password, that together would represent a traditional handwritten signature.

The Investigator will agree to the inspection of study-related records by the Sponsor, external auditor and/or health authority representatives.

10.4.3 Investigator Site File

All documents required for the conduct of the study as specified in the ICH-GCP guidelines and as defined by the sponsor will be maintained by the Investigator in an orderly manner and made available for monitoring and/or auditing by the Sponsor/or designee and regulatory agencies.

10.5 Clinical Trial Supplies

The Sponsor or designee will be responsible for supplying clinical trial supplies to the sites. Standard of care treatment, including G-CSF, will be provided by each site according to their standard of care. The Principal Investigator will be responsible for the administration, inventory and accountability of all clinical trial supplies provided to the site, exercising accepted medical and pharmaceutical practices. An accurate and timely record of the disposition of all clinical supplies must be maintained. The supplies and inventory record must be made available for inspection upon request. Upon completion or termination of the study, the Investigator will return the remaining clinical supplies along with a copy of the inventory record and a record of the clinical supplies returned. A copy of these records should be maintained in the site study files. **Under no circumstances will the Investigator allow the study drugs to be used other than as directed by this protocol.**

Clinical trial supplies include, but are not limited to: CRFs, laboratory supplies, rescue medications and study drugs.

10.6 Data Management

Data Management services will be provided by the Sponsor or designee. The data management system will be specified in the Data Management Plan.

After the data have been entered and verified, various edit checks will be performed for the purpose of ensuring the accuracy, integrity and validity of the database. These edit checks may include:

- Missing value checks
- Range checks
- Consistency checks
- Sequence checks
- Protocol adherence checks

Queries generated from these checks will be sent to the investigational site for resolution, and the database will be updated to reflect query resolutions as appropriate.

Adverse events will be coded using the latest version of MedDRA (currently version 20.0). Prior and concomitant medications will be coded according to the World Health Organization (WHO) Drug Dictionary.

11. STUDY ADMINISTRATION

11.1 REQUIRED DOCUMENTS PRIOR TO STUDY INITIATION

Prior to the start of this study, all pre-investigational requirements must be met by the Investigator and study site. These may include:

- Appropriate local health authority documentation properly signed and dated by the required Investigator (i.e., documents required for submission to the local IRB/IECs or applicable regulatory authorities).
- Signed copy (original) of the approved protocol
- Completed and signed statement of Investigator
- A signed Clinical Trial Agreement
- Curriculum vitae for the Investigator and sub-Investigator (can be collected at site initiation visit)
- IRB/IEC name and address; and membership list (can be collected at site initiation visit)
- Letter of approval from the IRB/IEC for both protocol (identified by protocol title and number) and ICF (identified by protocol title and number)
- Copy of the IRB/IEC-approved written ICF to be used in the study (that has also been approved by the Sponsor)
- Provisions for direct access to source/data documents, if necessary, for trial-related monitoring, audits, IRB/IEC review and regulatory inspection
- Name and location of the laboratory utilized for laboratory assays and other facilities conducting tests, as well as a copy of the laboratory certificate and list of normal laboratory values (can be collected at site initiation visit)
- In case a laboratory certification is not available, a written statement as to how the laboratory complies with quality assurance should be provided.

Upon satisfactory receipt of all required regulatory documents, the Sponsor will arrange for study drugs to be delivered to the study site. Supply of all other study materials will be the responsibility of the Sponsor and/or designee. Subject entry should not begin until after the required regulatory documents are confirmed as received and the Investigator Meeting/Initiation Meeting has occurred. All personnel expected to be involved in the conduct of the study will undergo study initiation to include review of study protocol, instructions for CRF completion, AE reporting and overall responsibilities including those for drug accountability and study file maintenance.

The Investigator and/or designee (study monitor) will be provided with an Investigator's File. This file should be used for all trial related documents. The Investigator will be responsible for keeping the Investigator's file updated and ensuring that all required documents are filed. The file will be inspected during monitoring visits.

11.2 STUDY COMPLETION

The core study is expected to end when the last randomized subject has completed the 100 days after transplantation and all query resolutions have been completed. Further the study will continue until the last randomized subjects complete 5 years. Data and materials that are

required before the study can be considered complete and/or terminated include, but are not limited to:

- Laboratory findings, clinical data and all special test results from screening through the end of the follow-up period
- CRF properly completed by appropriate study personnel and electronically signed by the Investigator
- Completed Drug Accountability Records
- Statement of outcome for each SAE reported
- Copies of protocol amendments and IRB/IEC as well as relevant health authority approval/notification (if applicable)
- Retention of Study Documents Statement

11.3 Clinical Study Report (CSR)

Clinical Study Report (CSR) and supplements will be developed by the Sponsor according to sequence of the below-planned analyses. The CSR and supplements will be a clinical and statistical integrated report, according to the ICH E3 guideline.

The sequence of planned statistical analyses for this study given in the planned chronological order is as follows:

- The core set of the study accumulated data will be made available for statistical analyses of the primary study endpoint, engraftment-derived secondary endpoints and secondary short-term graft durability endpoint on the day the last randomized subject completes the Core Study Termination Visit (Visit 13), which will occur at his/her Day 100 following transplantation. The results and conclusions obtained from the integrative assessment of these efficacy endpoints, as well as accumulated safety data up to this cut-off date, will be summarized in a Clinical Study Report (CSR) in support of regulatory approval of BL-8040 + G-CSF for the treatment of Multiple Myeloma.

- The second set of study accumulated data will be made available for statistical analyses of the long-term graft durability endpoints on the day the last randomized subject completes Visit 16, which will occur at 12 months following transplantation. The results and conclusions obtained from the integrative assessment of these secondary efficacy endpoints, as well as accumulated safety data up to this cut-off date, will be summarized in a supplement to the CSR in further support of regulatory approval of BL-8040 + G-CSF for the treatment of Multiple Myeloma.

- The third and last set of study accumulated data will be made available for statistical analyses of the long-term exploratory endpoints at 5 years following the randomization of the last study subject. The results and conclusions obtained from the integrative assessment of these exploratory efficacy endpoints will be summarized in an additional supplement to the CSR in further support of the regulatory approval of BL-8040 + G-CSF for the mobilization for autologous stem cell transplantation in Multiple Myeloma.

11.4 Retention of Study Records

The Investigator will retain copies of the approved protocol, completed CRF, signed ICFs, relevant source documents and all other supporting documentation related to the project as defined in ICH-E6 Section 8 related to the project per ICH-E6 record retention requirements. Records will be retained for at least 2 years after the last approval of a marketing application

in an ICH region and until there are no pending or contemplated marketing applications in an ICH region or at least 2 years have elapsed since the formal discontinuation of clinical development of the investigational product, in a secure and safe facility with limited access. If the Investigator is unable to retain the study documents for the required amount of time, the Sponsor or designee must be informed of the individual who will be assuming this responsibility.

The Sponsor will notify, in writing, the Investigator when the clinical study data may be discarded. The Investigator will take measures to prevent accidental or premature destruction of these documents.

These files must be made available for inspection upon reasonable request by authorized representatives of the Sponsor and/or the relevant regulatory agencies.

11.5 CONFIDENTIALITY AND PUBLICATION OF STUDY RESULTS

11.5.1 General

All data and information supplied by or on behalf of the Sponsor or otherwise acquired or obtained by any Research Institution, the Principal Investigator and other Investigators ("Recipients") in any manner, in connection with or in performance of this study, is considered "Confidential Information". This Confidential Information includes, but is not limited to, the Investigator's brochure, this protocol and any information relating thereto, CRFs and other scientific data, information relating to Sponsor's Investigational Product and treatment methodology and information relating to Sponsor's (or its affiliates') commercial, technical and financial information, research technology, products, inventions, trade secrets and research and development. The results produced in performance of the study and any data, information or other material collected, developed, generated or prepared during and in the course of performing the study shall be promptly disclosed to Sponsor in full in writing, and are also considered Confidential Information. This Confidential Information shall be and remain the sole property of the Sponsor. Except for Publishable Results (defined below) to the extent it may be published under Section 11.6.2, throughout the duration of the study and after its completion, Recipients shall (i) not disclose Confidential Information to others without the written consent of the Sponsor, except to those of its employees who have a need to know the Confidential Information in order to Recipients' obligations hereunder, and where such employees are bound by written contractual obligations covering Confidential Information that are no less restrictive or protective than those contained herein, provided that Recipients shall remain liable for any disclosure or use of Confidential Information by such employees, (ii) use the same degree of care to preserve confidentiality of Confidential Information as they use for their own information of like nature, which shall not be less than reasonable degree of care, and (iii) not use Confidential Information for any purpose except in the performance of this study. Promptly at Sponsor's request, or upon completion of the study, Recipients will discontinue use and return to Sponsor or destroy, in accordance with Sponsor's instructions, all copies or other manifestations of Confidential Information that may be in their possession or control, except to the extent expressly required hereunder and to comply with Applicable Laws (defined below). Should a Recipient be required to disclose Confidential Information pursuant to law, regulation, judicial or administrative order or request by a governmental or other entity authorized by law to make such request, Recipient shall (i) promptly notify Sponsor prior to such disclosure, (ii) cooperate with Sponsor and provide assistance in seeking a protective order or other suitable protection with respect to the Confidential Information, and (iii) only disclose such Confidential Information to the extent

pursuant to said law, regulation, judicial or administrative order, or request by a governmental or other authorized entity.

At the subject's request, medical information may be given to his or her personal physician or other appropriate medical personnel responsible for his or her welfare. The personal physician will be notified by site personnel of subject participation in the study.

11.5.2 Published Data

The object of the study will be to publish the results of the complete study ("Publishable Results") in an appropriate peer-reviewed journal after the conclusion of the study ("Publication"). A formal Publication of the Publishable Results is planned and will be considered a joint publication by the Principal Investigator, other Investigators, Sponsor and the appropriate Sponsor personnel (and Research Institutions) (*Subject to internal review)*. Publication must be undertaken in a responsible and ethical manner, taking into account relevant external standards regarding the manner and content of scientific, technical and medical publications and in subject to applicable laws, rules, regulations, policies and guidelines ("Applicable Laws"). Authorship will be determined by mutual agreement between Sponsor and Principal Investigator. Sponsor shall be mentioned in all Publications unless contrary instruction is given by Sponsor. Review and comment by Sponsor authorized personnel on draft abstracts and manuscripts for Publication or presentation is required prior to publication or presentation. Authors shall submit a copy of any abstracts, manuscripts or other material proposed for publication or presentation ("**Draft Publications**") to the Sponsor for its approval no fewer than sixty (60) days prior to the intended date of submission of such Draft Publications to any journal, publisher, and/or third party. The Sponsor has the right, at its discretion (a) to evaluate Draft Publications for accuracy and concurrence regarding data, evaluations, and conclusions, (b) to provide an opportunity for Sponsor to share with the Investigator(s) any new or unpublished information of which he or she may be unaware, (c) to ensure that no Confidential Information or other Sponsor proprietary information is being utilized and has been included, and (d) evaluate Draft Publications to determine if patent applications need to be filed on any information disclosed therein.

If the Sponsor determines that such Draft Publication contains Confidential Information or could otherwise be detrimental to Sponsor's intellectual property interest or have other adverse effects on its business, and notifies Principal Investigator of its determination, the Principal Investigator, Research Institutions and other Investigators/authors shall remove such Confidential Information from the Draft Publication or at Sponsor's election, modify it to remove language that is detrimental to Sponsor's intellectual property or other interests, and refrain from submitting such Draft Publication to a journal, publisher and/or other third party for additional ninety (90) days from Sponsor's notification to allow for filing of patent applications or the taking of such other measures as Sponsor deems appropriate to establish, preserve and protect its intellectual property or other interests. Principal Investigator, other Investigators and Research Institutions further agree to redact or modify those sections of the draft Publication which Sponsor in good faith determines falls within (a) to (d) above.

12. REFERENCES

1. Rajkumar SV. Multiple myeloma. *Curr Probl Cancer*. 2009;33(1):7-64. doi:10.1016/j.currproblcancer.2009.01.001.
2. Raab MS, Podar K, Breitkreutz I, Richardson PG, Anderson KC. Multiple myeloma. *Lancet Lond Engl*. 2009;374(9686):324-339. doi:10.1016/S0140-6736(09)60221-X.
3. Ludwig H, Beksac M, Bladé J, et al. Current multiple myeloma treatment strategies with novel agents: a European perspective. *The Oncologist*. 2010;15(1):6-25. doi:10.1634/theoncologist.2009-0203.
4. Kyle RA, Gertz MA, Witzig TE, et al. Review of 1027 patients with newly diagnosed multiple myeloma. *Mayo Clin Proc*. 2003;78(1):21-33. doi:10.4065/78.1.21.
5. Lin P. Plasma cell myeloma. *Hematol Oncol Clin North Am*. 2009;23(4):709-727. doi:10.1016/j.hoc.2009.04.012.
6. Brenner H, Gondos A, Pulte D. Recent major improvement in long-term survival of younger patients with multiple myeloma. *Blood*. 2008;111(5):2521-2526. doi:10.1182/blood-2007-08-104984.
7. Kumar SK, Rajkumar SV, Dispenzieri A, et al. Improved survival in multiple myeloma and the impact of novel therapies. *Blood*. 2008;111(5):2516-2520. doi:10.1182/blood-2007-10-116129.
8. Ludwig H, Durie BGM, Bolejack V, et al. Myeloma in patients younger than age 50 years presents with more favorable features and shows better survival: an analysis of 10 549 patients from the International Myeloma Working Group. *Blood*. 2008;111(8):4039-4047. doi:10.1182/blood-2007-03-081018.
9. Palumbo A, Anderson K. Multiple myeloma. *N Engl J Med*. 2011;364(11):1046-1060. doi:10.1056/NEJMra1011442.
10. Attal M, Harousseau J-L, Stoppa A-M, et al. A prospective, randomized trial of autologous bone marrow transplantation and chemotherapy in multiple myeloma. *N Engl J Med*. 1996;335(2):91–97.
11. Child JA, Morgan GJ, Davies FE, et al. High-dose chemotherapy with hematopoietic stem-cell rescue for multiple myeloma. *N Engl J Med*. 2003;348(19):1875–1883.
12. Fermand J-P, Katsahian S, Divine M, et al. High-dose therapy and autologous blood stem-cell transplantation compared with conventional treatment in myeloma patients aged 55 to 65 years: long-term results of a randomized control trial from the Group Myelome-Autogreffe. *J Clin Oncol Off J Am Soc Clin Oncol*. 2005;23(36):9227-9233. doi:10.1200/JCO.2005.03.0551.
13. Blystad AK, Delabie J, Kvaløy S, et al. Infused CD34 cell dose, but not tumour cell content of peripheral blood progenitor cell grafts, predicts clinical outcome in patients with diffuse large B-cell lymphoma and follicular lymphoma grade 3 treated with high-dose therapy. *Br J Haematol*. 2004;125(5):605-612. doi:10.1111/j.1365-2141.2004.04951.x.
14. Toor AA, Ayers J, Strupeck J, et al. Favourable results with a single autologous stem cell transplant following conditioning with busulphan and cyclophosphamide in patients with multiple myeloma. *Br J Haematol*. 2004;124(6):769-776.

15. Stiff P, Gingrich R, Luger S, et al. A randomized phase 2 study of PBPC mobilization by stem cell factor and filgrastim in heavily pretreated patients with Hodgkin's disease or non-Hodgkin's lymphoma. *Bone Marrow Transplant*. 2000;26(5):471-481. doi:10.1038/sj.bmt.1702531.
16. Tricot G, Jagannath S, Vesole D, et al. Peripheral blood stem cell transplants for multiple myeloma: identification of favorable variables for rapid engraftment in 225 patients. *Blood*. 1995;85(2):588-596.
17. Demirer T, Buckner CD, Gooley T, et al. Factors influencing collection of peripheral blood stem cells in patients with multiple myeloma. *Bone Marrow Transplant*. 1996;17(6):937-941.
18. Desikan KR, Barlogie B, Jagannath S, et al. Comparable engraftment kinetics following peripheral-blood stem-cell infusion mobilized with granulocyte colony-stimulating factor with or without cyclophosphamide in multiple myeloma. *J Clin Oncol Off J Am Soc Clin Oncol*. 1998;16(4):1547-1553. doi:10.1200/JCO.1998.16.4.1547.
19. Perea G, Sureda A, Martino R, et al. Predictive factors for a successful mobilization of peripheral blood CD34+ cells in multiple myeloma. *Ann Hematol*. 2001;80(10):592-597.
20. DiPersio JF, Stadtmauer EA, Nademanee A, et al. Plerixafor and G-CSF versus placebo and G-CSF to mobilize hematopoietic stem cells for autologous stem cell transplantation in patients with multiple myeloma. *Blood*. 2009;113(23):5720-5726. doi:10.1182/blood-2008-08-174946.
21. Abraham M, Beider K, Wald H, et al. The CXCR4 antagonist 4F-benzoyl-TN14003 stimulates the recovery of the bone marrow after transplantation. *Leukemia*. 2009;23(8):1378-1388.
22. Abraham M, Biyder K, Begin M, et al. Enhanced unique pattern of hematopoietic cell mobilization induced by the CXCR4 antagonist 4F-benzoyl-TN14003. *Stem Cells Dayt Ohio*. 2007;25(9):2158-2166.
23. Abraham M, Weiss ID, Wald H, et al. Sequential administration of the high affinity CXCR4 antagonist BKT140 promotes megakaryopoiesis and platelet production. *Br J Haematol*. 2013;163(2):248-259. doi:10.1111/bjh.12501.

13. APPENDICES

Appendix A: Schedule of Assessments

Table 5: BL-8040-SCM.301 (GENESIS) Schedule of Assessments – Treatment Period

Visit Name	Screening	Baseline	Visit Day 1-3	Visit Day 4		Visit Day 5	Visit Day 6[1]		Visit Day 7[1]	Visit Day 8[1]
Visit Day (window)	Day -28 to 0	Day 0 (-3 to 0)	AM	AM	PM	AM	AM	PM	AM	AM
			08:00 ±2 hrs	08:00 ±2 hrs	08:00 ±2 hrs	08:00 ±2 hrs	08:00 ±2 hrs	08:00 ±2 hrs	08:00 ±2 hrs	08:00 ±2 hrs
**Procedures**										
ICF process	X									
Inc/Exc criteria review	X	X								
Medical History	X									
Complete Physical Exam	X									
Directed Physical Exam[2]		X			X	X		X	X	X
Demographic Data[3]	X									
ECOG status	X									
IWRS	X		On Day 1 only	X				X[4]		
SC G-CSF ~10µg/kg/day			X	X		X	X		X	X
Premedication (Antihistamine)[5]					X			X		
SC BL-8040/Placebo 1.25 mg/kg[6]					X			X		
Apheresis[7]						X	X		X	X
Bone Marrow Biopsy[8]	X									
Adverse Events[9,10]	X---→									
Concomitant Medications[11]	X---→									
12 Lead ECG[10]	X	X			X[12]	X[13]	X[14]	X[12]	X[13]	X[14]
Vital Signs[10]	X	X			X2	X	X[15]	X2	X	X[16]
Weight, Height		X				X[17]				
Resources use and cost elements[18]			X---→							

Visit Name	Screening	Baseline	Visit Day 1-3	Visit Day 4		Visit Day 5	Visit Day 6[1]		Visit Day 7[1]	Visit Day 8[1]
Visit Day (window)	Day -28 to 0	Day 0 (-3 to 0)	AM	AM	PM	AM	AM	PM	AM	AM
			08:00 ±2 hrs	08:00 ±2 hrs	08:00 ±2 hrs	08:00 ±2 hrs	08:00 ±2 hrs	08:00 ±2 hrs	08:00 ±2 hrs	08:00 ±2 hrs
**Laboratory assessments - Local**										
CBC with differential[2,10]	X	X		X	X	X	X[19]	X	X	X[20]
Blood chemistry[2,10,21]	X	X		X		X	X[22]		X	X[22]
Coagulation Test	X					X			X	
Serology assessment[23]	X									
CD34+ enumeration PB[24]		X		X		X	X		X	X
CD34+ enumeration in Apheresis Product[25]						X	X		X	X
Pregnancy Test (urine/serum)	X[26]	X								
Serum and Urine Monoclonal (M) Protein (i.e. UPEP/SPEP) w/ Immunofixation	X									
Serum Free Light Chain (FLC)	X									
Urinalysis	X									
**Laboratory Assessments - Central**										
PK Assessment[27,28]					X	X		X	X	
ADA[29]					X					
Complement & Mast Cells Activation[30]					X					
CD34+ enumeration in PB[24]		X		X		X	X		X	X
CD34+ enumeration in Apheresis Product[25]						X	X		X	X
Lymphocyte subsets in Apheresis Product **only in Part 2**						X	X		X	X
Multiple Myeloma Cells in Apheresis Product **only in Part 2**						X	X		X	X

[1] Dosing during Day 6, 7 and 8 will be conducted upon failure to collect ≥ $6x10^6$ CD34+ cells in the apheresis content.
[2] To be performed pre-dose
[3] Includes race, gender, etc.
[4] For patients who need 2nd BL-8040 dose administration
[5] Within one hour prior to BL-8040/Placebo administration if premedication is given orally, or ~15 minutes prior to study drug administration if premedication is given IV
[6] In Part 1, BL-8040 administration only
[7] Following G-CSF administration
[8] Screening biopsy will be required only for CR/sCR patients. A bone marrow biopsy sample taken up to 30 days before signing the ICF may be used for screening.
[9] All AEs will be collected starting from ICF signature and up to 30 days from the last dose of study drug
[10] For subjects with moderate or severe renal impairment with abnormal clinically significant changes after the first BL-8040/Placebo dose, this procedure will be performed weekly until recovery or stabilization. Biochemistry should be performed with GFR assessment.
[11] From Visit 11 and on only disease related medications will be recorded
[12] Triplicate ECGs Pre-dose (up to 1 hour before BL-8040 administration) and 30-45 minutes post-dose
[13] Triplicate ECGs prior to apheresis and G-CSF administration
[14] ECG for local assessment will be done ONLY for moderate/severe renal impairment subjects.
[15] Will be done ONLY for moderate/severe renal impairment subjects.
[16] Will be done for all moderate/severe renal impairment subjects or for subjects who did not achieve collection goal at Day 7.
[17] At Day 5, only weight will be recorded and will be measured prior to apheresis
[18] Only if G-CSF was administered at the site.
[19] Will be done for all moderate/severe renal impairment subjects regardless of the amount of cells collected in the apheresis and for subjects who did not achieve collection goal at Day 5.
[20] Will be done for all moderate/severe renal impairment subjects who received second dose of BL-8040/Placebo regardless of the amount of cells collected in the apheresis and for subjects who did not achieve collection goal at Day 7.
[21] Blood biochemistry includes Na+, K+, calcium, uric acid, phosphorus, glucose, total bilirubin, AST, ALT, LDH, ALP, BUN and creatinine.
[22] Will be done for all moderate/severe renal impairment subjects or for subjects who did not achieve collection goal at day 7.
[23] Serology assessment includes HIV antibodies (HIV1 and HIV2), Hepatitis B surface antigen (HBsAg) and Hepatitis C antibody (Hep C Ab).
[24] $CD34^+$ Enumeration in PB should be collected before G-CSF administration. This assessment will be done locally in Part 1 and locally and at the central laboratory in Part 2
[25] $CD34^+$ Enumeration in Apheresis Product will be done by locally in Part 1 and locally and at the central laboratory in Part 2
[26] Female subjects must be of non-childbearing potential or, if of childbearing potential, must have a negative serum pregnancy test at screening and negative urine/serum pregnancy test within 72 hours prior to G-CSF first administration.
[27] Blood samples for BL-8040 or its metabolites PK analysis will be collected during Part 1 on Day 4 and Day 6 at pre-dose of BL-8040 and 0.25, 0.5, 1, 2, 4 and 8 hrs post BL-8040 administration. Additional PK sample will be collected 12 hrs (+/-2hrs) post BL-8040 administration, before the apheresis on Days 5 and 7. Please refer to the laboratory manual for additional information.

[28] Blood samples for BL-8040 or its metabolites PK analysis will be collected during Part 2 on Days 4 and 6 at pre-dose of BL-8040 and 40-60 minutes and 2-6 hours post BL-8040 administration. Additional PK sample will be collected 10-14 hours post BL-8040 administration, before the apheresis on Days 5 and 7. Please refer to the laboratory manual for additional information.

[29] Blood samples for ADA for both parts will be collected on Day 4 up to 1 hour pre-dose of BL-8040 and on Visits 9 (pre chemotherapy) and 11 Please refer to the laboratory manual for additional information.

[30] Blood samples for Mast Cell Activation and Complement Activation in both parts of the study will be collected on Day 4 up to 1 hour pre-dose of BL-8040 and 2 hours post BL-8040 administration (Post dose defined as end time of administration) (±15 minutes). Please refer to the laboratory manual for additional information.

Table 6: BL-8040-SCM.301 (GENESIS) Schedule of Assessments – Post Treatment Period

	Core Study						Long-Term disease assessment Follow-Up post transplantation			
	Visit 9 Conditioning[1] (Window up to -3 days)	Visit 10[2]		Visit 11 Day 30 (± 3d) Post-transplant	Visit 12 Day 60 (± 7d) Post-transplant	Visit 13 Day 100 (± 7d) Post-transplant Termination/ Early Termination	Visit 14 Day 180 (± 14d)	Visit 15 Day 270 (± 14d)	Visit 16 12 Months (± 14d)	Visits 17-18 Months and up to 5 years[3] (± 30d)
		Transplant (prior to transplant)	Day 1 to 29 Post-transplant	Post-transplant	Post-transplant	Post-transplant				
**Procedures**										
Complete Physical Exam	X					X				
Directed Physical Exam		X								
ECOG status					X	X	X	X	X	
Vital Signs	X	X		X	X	X				
Concomitant Medications[4]	X---→						X-------------------------→			
Bone Marrow and Biopsy[5]						X				
OS and RFS					X	X	X	X	X	X[6]
Resources use and cost elements	X---→									
**Laboratory assessments- Local**										
CBC	X	X	X[7]	X	X	X	X	X	X	
Blood chemistry[8]	X	X		X	X	X	X	X	X	
Pregnancy Test in urine/serum	X									
Serum and Urine Monoclonal (M) Protein (i.e. UPEP[9] and SPEP) w/ Immunofixation				X	X	X	X	X	X	
Serum Free Light Chain (FLC)				X	X	X	X	X	X	

	Core Study						Long-Term disease assessment Follow-Up post transplantation			
	Visit 9 Conditioning[1] (Window up to -3 days)	Visit 10[2]		Visit 11 Day 30 (± 3d) Post-transplant	Visit 12 Day 60 (± 7d) Post-transplant	Visit 13 Day 100 (± 7d) Post-transplant Termination/ Early Termination	Visit 14 Day 180 (± 14d)	Visit 15 Day 270 (± 14d)	Visit 16 12 Months (± 14d)	Visits 17-18 Months and up to 5 years[3] (± 30d)
		Transplant (prior to transplant)	Day 1 to 29 Post-transplant	Post-transplant	Post-transplant	Post-transplant				
**Laboratory assessments- Central**										
ADA[10]	X			X						

[1] Conditioning must occur within 5 weeks after the last apheresis session

[2] Transplantation will occur ~24hrs after completion of the conditioning regimen and no later than 5 weeks after the last apheresis session.

[3] From Visit 17 (inclusive), follow up will be only through phone calls.

[4] Information on concomitant medications should be collected until Visit 10 inclusive; After Visit 10 only information on treatments of multiple myeloma are required. Beginning on Day 60 post-transplantation, special attention is requested in reporting the treatments provided for maintenance.

[5] The second bone marrow biopsy at Day 100 will only be performed if biochemical tests do not show evidence of relapse.

[6] Phone call every 6 months

[7] Daily until engraftment. For outpatient, only ANC and platelets collection is required

[8] Blood chemistry includes Na+, K+, calcium, glucose, uric acid, phosphorus total bilirubin, AST, ALT, LDH, ALP, BUN, Creatinine.

[9] UPEP will be performed as 24-hour urine collection

[10] Blood samples for ADA for both parts will be collected on Visit 9 (pre-chemotherapy) and Visit 11. Please refer to the laboratory manual for additional information.

Appendix B: Eastern Cooperative Oncology Group Performance Status

**Eastern Cooperative Oncology Group (Zubrod-ECOG)[a,b]**	
**Description**	**Grade**
Fully active, able to carry on all pre-disease activities without restriction.	0
Restricted in physically strenuous activity but ambulatory and able to carry out work of a light or sedentary nature e.g. light house work, office work.	1
Ambulatory and capable of all self-care but unable to carry out any work activities. Up and about more than 50% of waking hours.	2
Capable of only limited self-care, confirmed to bed or chair more than 50% of waking hours.	3
Completely disabled. Cannot carry on any self-care. Totally confined to bed or chair.	4

[a] Zubrod, C.G., et al. Appraisal of Methods for the Study of Chemotherapy of Cancer in Man. Journal of Chronic Diseases, 11:7-33, 1960.

[b] Oken, M.M., et al. Toxicity and response criteria of the Eastern Cooperative Oncology Group. Am J Clin Oncol (CCT) 5: 649-655, 1982

Appendix C: International Myeloma Working Group (IMWG) Uniform Response Criteria

Response criteria for the purposes of study stratification will be defined in accordance with the International Myeloma Working Group (IMWG) Uniform Response Criteria as below. Additionally, we will combine Stringent complete response (sCR) and Complete Response (CR) into one “CR” group and Very good partial response (VGPR) and Partial response (PR) into one “PR” group.

**Combined response groups defined for the study**	**IMWG Response**	**IMWG Criteria**
CR	Stringent complete response (sCR)	o CR as defined below<br>o Normal free light chain ratio (0.26-1.65)<br>o Absence of clonal cells in the bone marrow by immunohistochemistry or immunofluorescence
	Complete response (CR)	o Disappearance of monoclonal protein by both protein electrophoresis and immunofixation studies from the blood and urine<br>o If serum and urine monoclonal protein are unmeasurable, Normal free light chain ratio (0.26-1.65)<br>o <5% plasma cells in the bone marrow<br>o Disappearance of soft tissue plasmacytoma
PR	Very good partial response (VGPR)	o Serum and urine monoclonal protein detectable by immunofixation but not on electrophoresis OR ≥ 90% reduction in serum monoclonal protein with urine monoclonal protein < 100 mg per 24 hours<br>o If serum and urine monoclonal protein are unmeasurable, a > 90% decrease in difference between the involved and uninvolved free light chain levels is required in place of monoclonal protein criteria (The absolute decrease must be > 10 mg/dL)<br>o If present, > 50% reduction in the size of soft tissue plasmacytomas (by clinical or radiographic examinations)
	Partial response (PR)	o > 50% reduction in the level of the serum monoclonal protein<br>o Reduction in urine monoclonal protein by either > 90% or to < 200 mg<br>o If serum and urine monoclonal protein are unmeasurable, a >50% decrease in difference between the involved and uninvolved free light chain levels is required in place of monoclonal protein criteria (The absolute decrease must be > 10 mg/dL)<br>o If serum and urine monoclonal protein are unmeasurable and serum free light chain is unmeasurable, a > 50% reduction in plasma cells is required in place of monoclonal protein, provided that baseline bone marrow plasma cell percentage was > 30%<br>o If present, > 50% reduction in the size of soft tissue plasmacytomas (by clinical or radiographic examinations).

Appendix D: CIBMTR (Engraftment Guideline)

The following is an excerpt of CIBMTR Form 2450 Post-TED Instructions for Post-Transplant Essential Data (Post-TED), Document Number: A00425 version 2.0 (8/01/2012) (pages 9-11). The full form is available at https://www.cibmtr.org/DataManagement/TrainingReference/Manuals/DataManagement/Documents/post-ted-instruction.pdf

Special attention should be paid to **platelet engraftment day calculation examples**.

For more information regarding reporting partial or unknown dates, see General Instructions, General Guidelines for Completing Forms.

Question 10: Date of last assessment:
If ANC of ≥ 0.5 x 10^9/L was not achieved for three or more consecutive days, enter the date of the last laboratory report.

For more information regarding reporting partial or unknown dates, see General Instructions, General Guidelines for Completing Forms.

Question 11: Did *graft failure* occur?
Graft failure includes persistent neutropenia, < 5% donor chimerism, and ANC < 0.5 x 10^9/L for three or more consecutive laboratory values. Graft failure often requires an additional infusion of donor cells. Graft failure may result from the use of specific drugs, infection (especially CMV), GVHD, and other etiologies.

If the recipient meets the criteria of graft failure, check "yes."

Initial Platelet Recovery

NOTE: Transfusions
Currently there is an error on the Form 2450 regarding the date of platelet recovery. The form should read: "date platelet greater than **or equal to (≥)** 20 x 10^9/L."

The following questions refer to **initial** platelet recovery following the HSCT for which this form is being completed. All dates should reflect **no platelet transfusions administered for seven consecutive days**. Report the date of the first of three consecutive laboratory values ≥ 20 x 10^9 L obtained on different days, as shown in example 3 below. Note that platelet recovery may take place well after the recipient has returned to the referring physician for care. It is essential that information and laboratory values be obtained from the referring physician.

Transfusions temporarily increase blood cell counts. When the data is later used for analysis, it is important to be able to distinguish between a recipient whose own body was creating the cells and a recipient who required transfusions to support the counts.

The following example illustrates the procedure to follow for reporting platelet recovery.

Example 3: Reporting Platelet Recovery

	Transfusion ↓										
Day	0	1	2	3	4	5	6	7	8	9	10
Platelet Count	10,000	35,000	30,000	25,000	10,000	15,000	19,000	23,000	25,000	40,000	50,000
Date	1/1/2008	1/2/2008	1/3/2008	1/4/2008	1/5/2008	1/6/2008	1/7/2008	1/8/2008	1/9/2008	1/10/2008	1/11/2008
								↑ 1st of 3			

Report 1/8/08 as date platelet count ≥ 20 x 10^9/L

Question 12: Initial platelet recovery
Indicate whether or not there was evidence of **initial** platelet recovery following this HSCT.

Check only **one** response:

- If "yes," continue with question 13.
- If "no," continue with question 14.
- Check "never below," if the recipient's platelets never dropped below 20 x 10^9/L at any time post-HSCT and a platelet transfusion was never required. If the recipient's platelet count drops below 20 x 10^9/L and/or the recipient received a platelet transfusion even once, do not use this option.
- Check "previously reported" if this is the six-month or annual follow-up, and initial platelet recovery has already been reported on a previous form.
- Check "unknown" if there is no documentation of platelet recovery and/or laboratory reports cannot be obtained.

Question 13: Date platelet ≥ 20 x 10^9/L
Enter the **first** date of three consecutive laboratory values obtained on different days where the platelet count was ≥ 20 x 10^9/L. Ensure that no platelet transfusions were administered for seven days immediately preceding this date. Include day seven, as shown in Example 3 above, when determining the recovery date.

If three laboratory values were not obtained on consecutive days, but a sequential rise of ≥ 20 x 10^9/L is demonstrated, follow the examples below when determining an estimated date.

Examples:
A. The recipient is being seen in the outpatient clinic and receives a platelet transfusion on January 1. The platelet count is ≥ 20 x 10^9/L on January 2, January 3, and January 4. The recipient does not come into the clinic for evaluation until one month later. The recipient has not received any more platelet transfusions and the platelet count is well above 20 x 10^9/L. Report January 8 (day seven post-platelet transfusion) for the date of platelet recovery.

B. The recipient is being seen in the outpatient clinic and receives a platelet transfusion on January 1. The platelet count is ≥ 20 x 10^9/L on January 2, January 3, and January 4. The recipient is then discharged back to their primary care physician. The transplant center receives a follow-up note from the primary care physician that states "recipient recovered their platelets in January of 2011." Report the day of the month as the *15th*. If the *15th* does not make logical sense in relation to the dates of the platelet counts obtained, use either the *1st* or *30th*. Report month and year as documented.

For more information regarding reporting partial or unknown dates, see General Instructions, General Guidelines for Completing Forms.

Question 14: Date of last assessment
If a platelet count of ≥ 20 x 10^9/L was not achieved; enter the date of the last laboratory report.

For more information regarding reporting partial or unknown dates, see General Instructions, General Guidelines for Completing Forms.

Graft versus Host Disease (allogeneic only)

Graft versus Host Disease (**GVHD**) is an immunological phenomenon resulting from the reaction of donor immune cells against major or minor histocompatibility antigens of the recipient. GVHD is primarily caused by donor-derived T-cells. Very rarely, GVHD may occur due to autologous reactivity (autologous GVHD), third party transfusions, or with identical twin transplantation. Due to the rarity of this occurrence, the GVHD section should only be completed for allogeneic transplants. **For autologous HSCT, leave questions 15-18 blank.**

Factors influencing the severity of GVHD are related to three main categories: 1) donor or graft, 2) recipient, and 3) treatment. The most influential donor/graft factor is the degree of genetic disparity between the donor and the recipient (HLA match), but other risk factors include female donor to male recipient, donor parity, older donors, and T-cell dose. The occurrence of acute GVHD becomes a risk factor for the development of chronic GVHD. Recipient age and prior infections are also factors. Treatment-related factors include a myeloablative preparative regimen and inadequate post-HSCT immune suppression (GVHD prophylaxis).
