# Supplementary material for: Motixafortide and G-CSF to mobilize hematopoietic stem cells for autologous transplantation in multiple myeloma: a randomized phase 3 trial
Source: Nat Med. 2023 Apr 17;29(4):869–79. doi: 10.1038/s41591-023-02273-z (PMC10115633; doi:10.1038/s41591-023-02273-z)
Supplement: Supplementary file 2 — Reporting Summary [file 41591_2023_2273_MOESM2_ESM.pdf]

nature portfolio

Corresponding author(s): Zachary D. Crees

Last updated by author(s): Feb 18, 2023

Reporting Summary

Nature Portfolio wishes to improve the reproducibility of the work that we publish. This form provides structure for consistency and transparency in reporting. For further information on Nature Portfolio policies, see our Editorial Policies and the Editorial Policy Checklist.

Statistics

For all statistical analyses, confirm that the following items are present in the figure legend, table legend, main text, or Methods section.

n/a	Confirmed	
☐	☒	The exact sample size (*n*) for each experimental group/condition, given as a discrete number and unit of measurement
☐	☒	A statement on whether measurements were taken from distinct samples or whether the same sample was measured repeatedly
☐	☒	The statistical test(s) used AND whether they are one- or two-sided<br>*Only common tests should be described solely by name; describe more complex techniques in the Methods section.*
☐	☒	A description of all covariates tested
☐	☒	A description of any assumptions or corrections, such as tests of normality and adjustment for multiple comparisons
☐	☒	A full description of the statistical parameters including central tendency (e.g. means) or other basic estimates (e.g. regression coefficient) AND variation (e.g. standard deviation) or associated estimates of uncertainty (e.g. confidence intervals)
☐	☒	For null hypothesis testing, the test statistic (e.g. *F*, *t*, *r*) with confidence intervals, effect sizes, degrees of freedom and *P* value noted<br>*Give P values as exact values whenever suitable.*
☒	☐	For Bayesian analysis, information on the choice of priors and Markov chain Monte Carlo settings
☐	☒	For hierarchical and complex designs, identification of the appropriate level for tests and full reporting of outcomes
☐	☒	Estimates of effect sizes (e.g. Cohen's *d*, Pearson's *r*), indicating how they were calculated

Our web collection on statistics for biologists contains articles on many of the points above.

Software and code

Policy information about availability of computer code

Data collection	OnCore clinical research management software v1.0.0 was used at Washington University.
Data analysis	Commercially available R, SPSS, SAS and Xcel statistical and graphics software packages were used for data analysis and graphics.<br>Monocle3 (https://cole-trapnell-lab.github.io/monocle3/) was used for pseudotime analysis. Analysis was completed following standard tutorial for constructing single-cell trajectories (https://cole-trapnell-lab.github.io/monocle3/docs/trajectories/).<br>Bioconda, (The Bioconda Team et al., 2018), https://bioconda.github.io/<br>Bioconductor v3.9, (Huber et al., 2015), https://bioconductor.org/<br>Cell Ranger v6.0.1, 10X Genomics, https://support.10xgenomics.com/single-cell-gene-expression/software/pipelines/latest/what-is-cell-ranger<br>data.table_1.12.6 , R Development Core Team, https://cran.r-project.org/package=data.table<br>dendsort_0.3.3, (Sakai et al., 2014), https://cran.r-project.org/package=dendsort<br>dplyr_0.8.5, R Development Core Team, https://cran.r-project.org/package=dplyr<br>ggplot2_3.3.2, R Development Core Team, https://CRAN.R-project.org/package=ggplot2<br>gridExtra_2.3, R Development Core Team, https://cran.r-project.org/package=gridExtra<br>magrittr_1.5, R Development Core Team, https://cran.r-project.org/package=magrittr<br>Matrix_1.2-17 , R Development Core Team, https://CRAN.R-project.org/package=Matrix<br>pheatmap_1.0.12, R Development Core Team, https://cran.r-project.org/package=pheatmap<br>Python v3.7, Python Software Foundation, https://www.python.org/<br>R v3.6, R Development Core Team, https://www.r-project.org/<br>RColorBrewer_1.1-2, R Development Core Team, https://CRAN.R-project.org/package=RColorBrewer<br>reshape2_1.4.3, R Development Core Team, https://cran.r-project.org/package=reshape2<br>SAS v9.4 for statistical analyses

Seurat v3.1.2 and v4.0.3, (Butler et al., 2018), https://cran.r-project.org/web/packages/Seurat
single-cell analysis code, (https://github.com/reykajayasinghe/WashU_Genesis_Study).
sva v3.40.0, (Huber et al., 2015), https://bioconductor.org/packages/release/bioc/html/sva.html
stringr_1.4.0, R Development Core Team, https://cran.r-project.org/package=stringr
Subread v2.0.1, (Liao et al., 2013), https://sourceforge.net/projects/subread/
Tidyverse, (Wickham et al., 2019), https://www.tidyverse.org/
viridis_0.5.1, R Development Core Team, https://github.com/sjmgarnier/viridis
viridisLite_0.3.0, R Development Core Team, https://github.com/sjmgarnier/viridis

For manuscripts utilizing custom algorithms or software that are central to the research but not yet described in published literature, software must be made available to editors and reviewers. We strongly encourage code deposition in a community repository (e.g. GitHub). See the Nature Portfolio guidelines for submitting code & software for further information.

Data

Policy information about availability of data

All manuscripts must include a data availability statement. This statement should provide the following information, where applicable:

- Accession codes, unique identifiers, or web links for publicly available datasets
- A description of any restrictions on data availability
- For clinical datasets or third party data, please ensure that the statement adheres to our policy

All data and/or supporting documents related to the manuscript that Nature reviewers and/or editors will request for the purposes of evaluating this manuscript and verifying its contents will be provided through Egnyte system, at any time. Data will also be available to researchers and/or scientists in alignment with the ICMJE's policy on clinical data sharing. Specifically, the authors will provide access to individual deidentified participant level data that underly the data presented in this manuscript, including data dictionaries, the study protocol and other relevant information to any researcher who provides a methodologically sound proposal for academic purposes beginning 6 months and ending 5 years after article publication. These data can be requested via email to iritg@biolinerx.com, and will be made available to the requesting parties through the Egnyte system upon approval. This request and availability mechanism for accessing the clinical dataset will similarly apply to requests for the "minimum dataset" necessary to interpret, verify and extend the research in the article. All requests for clinical data will be reviewed by the sponsor (BioLineRx) to verify whether the request is subject to any intellectual property or confidentiality obligations. The gene count matrices for the single-cell RNA sequencing are available via the gene expression omnibus (accession #: GSE223972). References (GRCh38 genome reference namely refdata-gex-GRCh38-2020-A) used for single-cell analysis of the human genomes are available from public sources: https://support.10xgenomics.com/single-cell-gene-expression/software/release-notes/build.

Human research participants

Policy information about studies involving human research participants and Sex and Gender in Research.

Reporting on sex and gender

The findings in this study should be applicable to all sexes and genders. Neither sex nor gender were criteria for participation in this clinical trial and there were no pre-specified or post-hoc analyses of these data based solely on sex or gender. Patient sex was recorded for demographic purposes and was self-reported by study participants. Multiple myeloma occurs more commonly in male sex as compared to female sex, thus the slightly higher proportion of males enrolled on study is expected and is representative of the overall demographics of the study population.

Population characteristics

Eligible patients to be enrolled on this study included those ages 18-78 years with a diagnosis of multiple myeloma in their 1st or 2nd complete or partial remission (per IMWG criteria) with an ECOG performance status of 0-1 and adequate organ function (defined in the protocol) who were otherwise considered to be eligible for autologous hematopoietic stem cell transplantation.

Recruitment

Research participant recruitment may vary based on institutional and individual provider practices. Therefore, there is the possibility for recruitment heterogeneity and selection-bias to occur on multi-institutional studies such as the GENESIS Trial. Patients on the GENESIS Trial were recruited at participating institutions based on the presence of objective criteria, including the requisite diagnosis of biopsy proven multiple myeloma and a set of pre-defined eligibility criteria in order to minimize selection bias. To the authors knowledge, all patients meeting these objective criteria were offered participation in the study by their treating providers. All patients who were willing to participate and who provided informed consented were formally screened and, if determined to be eligible, were enrolled on study. Therefore, while the authors cannot entirely rule out the possibility of selection bias and other forms of recruitment bias from impacting the results of this study, it is likely that the objective nature of the inclusion/exclusion criteria applied uniformly across participating sites along with the randomized, placebo-controlled, double-blinded nature of the study may have minimized the impact of such potential biases.

Ethics oversight

Local IRB/IEC as well as central IRB and Central EC approval of the study protocol and amendments, investigator brochure and amendments, informed consent and any other documents provided to the subject was required at all participating sites (See list below of site-specific IRB details).
Site 1001 IRB:
Washington University Institutional Review Board (IRB)
4590 Children's Place, Suite 2300
St. Louis, MO 63110

Site 1002 IRB:
University of Miami IRB
Sylvester Comprehensive Cancer Center
Fox Building, Suite 300, 1550 NW 10th Avenue
Miami, Florida 33136

Site 1003:
Mayo Clinic IRB
200 First St SW
Rochester, MN 55905

Site 1005:
University of Oregon Oregon Health & Science University IRB
Knight Clinical Research
3485 SW Bond Ave, Mailcode OC13CT
Portland, OR 97239

Site 1006:
Human Research Protection Program
University of Kansas Medical Center IRB
Fairway North Office Building at 4330 Shawnee Mission Parkway, Suite 3170,
Fairway, KS 66205

Site 1007:
University of Maryland IRB
620 W. Lexington St., Second Floor
Baltimore, MD 21201

Site 1008:
Western Cooperative Group IRB
1019 39th Ave SE, Suite 120
Puyallup, WA 98374

Site 1009:
The Loyola University Chicago Health Sciences Campus IRB
1101 Wootton Parkway, Suite 200
Rockville, MD 20852

Site 1011:
Mayo Clinic IRB
200 First Street SW
Rochester, MN, USA

Site 1012:
UCLA IRB
10889 Wilshire Blvd, Suite 830
Los Angeles, CA 90095-1406

Site 1013:
University of Utah IRB
Research Administration building
75 South 2000 East
Salt Lake City, UT 84112

Site 1014:
Western Cooperative Group IRB
1019 39th Ave SE, Suite 120
Puyallup, WA 98374-2115

Site 1015:
University of Cincinnati IRB
University Hall, Suite 300 P.O. Box 210567
Cincinnati, OH 45221-0567

Site 1016:
The Weill Cornell Medical College IRB
1300 York Avenue, Box 89
New York, NY 10065

Site 1017:
UT M.D. Anderson Cancer Center IRB
7007 Bertner Avenue, Unit 1637
Houston, TX 77030

Site 4041:
Ethics Committee Catania 1 Azienda Ospedaliero-Universitaria “Policlinico-Vittorio Emanuele” Catania
Via Santa Sofia 78 – 95123 Catania

Site 4042:
Ethics Committee South Reggio Calabria Division Grande Ospedale Metropolitano “Bianchi-Melacrino- Morelli”
Via Provinciale Spirito Santo, Palazzo Gangeri, 24 89128 - Reggio Calabria

Site 5051:
University of Cologne Ethics Committee
50931 Cologne

Site 8081:
University of Debrecen, Clinical Center, Clinic of Internal Medicine, Hematology
Scientific Council for Health - Ethical Committee for Clinical Pharmacology
Széchenyi István tér 7-8.
Roosevelt Office Building II. floor 214 office H-1051
Budapest, Hungary

Site 8082:
Central Hospital of Southern Pest, National Institute of Hematology and Infectious Diseases
Scientific Council for Health - Ethical Committee for Clinical Pharmacology
Széchenyi István tér 7-8.
Roosevelt Office Building II. floor 214 office H-1051
Budapest, Hungary

All patients provided written informed consent prior to enrollment on the study. The study was conducted in accordance with the Guideline for Good Clinical Practice ICH E6(R2)- ICH Harmonized Guideline Integrated Addendum to ICH E6 (R1) (International Conference on Harmonization of Technical Requirements for the Registration of Pharmaceuticals for Human Use), Step 5, 14 June 2017; the Declaration of Helsinki: Seoul, 2008; the US Code of Federal Regulations (Title 21, CFR Part 11, 50, 54, 56 and 312) and/or EU Directives; and/or local country regulations and guidelines.

Note that full information on the approval of the study protocol must also be provided in the manuscript.

Field-specific reporting

Please select the one below that is the best fit for your research. If you are not sure, read the appropriate sections before making your selection.

☒ Life sciences ☐ Behavioural & social sciences ☐ Ecological, evolutionary & environmental sciences

For a reference copy of the document with all sections, see nature.com/documents/nr-reporting-summary-flat.pdf

Life sciences study design

All studies must disclose on these points even when the disclosure is negative.

Sample size	Sample size determination for Part 2 of the GENESIS study was based on plerixafor public domain data (DiPersio et al 2009, DOI 10.1182/blood-2008-08-174946). The primary study endpoint of the study was the proportion (%) of subjects mobilizing ≥6.0 x 10^6 CD34+ cells/kg with up to 2 apheresis sessions in preparation for auto-HCT after G-CSF + single administration of motixafortide/placebo. Subjects were randomized to treatment with motixafortide+G-CSF or to placebo+G-CSF using a 2:1 randomization ratio, respectively. According to DiPersio et al, it is assumed that the response rate of subjects randomized to treatment with G-CSF+Placebo will be 35%. It was also assumed that the minimal effect size required, in terms of proportions difference, is 35%. Accordingly, it was assumed that the response rate of motixafortide treated subjects will be 70% or more. In addition, a conservative assumption was made that 20% of the subjects randomized to treatment with BL-8040 will not adhere to treatment for whatever reason (e.g. early termination, lack of compliance) and therefore will have a placebo-like response rate of 35%. This assumption was made to ensure that the study will not be underpowered. Accordingly, the assumed combined response rate of 63% for subjects randomized to treatment with BL-8040 reflects the assumption that 80% of the BL-8040 treated subjects will have a response rate of 70%, while the remaining 20% of subjects (i.e., those who did not adhere to the planned treatment regimen) will have a response rate of 35%. According to these assumptions, a total of 147 subjects provides 89.8% power to at a two-sided alpha of 0.0466256. Incorporating an additional withdrawal rate of 20%, the sample size of Part 2 of the GENESIS study is inflated by 20% and therefore the study was designed to randomize a total of 177 subjects. The power at interim analysis (to be conducted at alpha=0.0108678), assuming an attrition rate of 20% and success rates of 70% and 35% for BL-8040 + G-CSF or placebo + G-CSF, respectively, is 85.9%. SAS® PROC POWER for two proportions was used to determine the sample size.
Data exclusions	The data was analyzed on an intent-to-treat principle, therefore no data were excluded after patients were enrolled and randomized to treatment on protocol. The correlative studies were performed only on samples collected from patients treated at Washington University, therefore patients treated on the GENESIS Trial protocol at other institutions were excluded from these analyses, by definition.
Replication	Replication was not performed on clinical specimens from the GENESIS Trial nor correlative studies involving human subjects. This was due to the finite number of HSPCs per sample, the limited number of aliquots of apheresis product available from each patient and the need to reserve the maximum possible amount of apheresis product for clinical use for patients HSPC infusion.
Randomization	Randomization was performed in a 2:1 fashion on the GENESIS Trial. Patients mobilized on the correlative protocol were treated according to standard of care, with no randomization needed/performed. However, patients mobilized on the correlative protocol were demographically similar to patients in the GENESIS Trial in terms of co-variates relevant to HSPC mobilization, including age, diagnosis, number of prior lines of therapy and lenalidomide exposure. In this way, co-variates between the randomized, double-blind placebo-controlled cohorts on the GENESIS Trial and the parallel correlative study were controlled for to the degree possible given the experimental design.
Blinding	This was a double-blinded, placebo-controlled clinical trial, with both investigators and patients blinded to study treatment allocation until completion of the trial and database lock, as per the supplemental GENESIS Trial Protocol and SAP.

Reporting for specific materials, systems and methods

We require information from authors about some types of materials, experimental systems and methods used in many studies. Here, indicate whether each material, system or method listed is relevant to your study. If you are not sure if a list item applies to your research, read the appropriate section before selecting a response.

Materials & experimental systems

n/a	Involved in the study
☐	☒ Antibodies
☒	☐ Eukaryotic cell lines
☒	☐ Palaeontology and archaeology
☒	☐ Animals and other organisms
☐	☒ Clinical data
☒	☐ Dual use research of concern

Methods

n/a	Involved in the study
☒	☐ ChIP-seq
☐	☒ Flow cytometry
☒	☐ MRI-based neuroimaging

Antibodies

Antibodies used	For local laboratory CD34+ enumeration at each participating clinical site, CD34+ enumeration was performed in accordance with standard clinical protocols at the local institution, which commonly involve adherence to ISHAGE guidelines (PMID: 8817388) in a CLIA-certified or a commensurately credentialed lab based on the specific site location/country. For central laboratory CD34+ enumeration at each participating clinical site, CD34+ enumeration of apheresis and peripheral whole blood samples was performed using validated methods in accordance with standard clinical protocols using ISHAGE guidelines (PMID: 8817388) in two CLIA-certified central labs based on site location (EU or USA). In these assays, the following fluorochrome-labeled surface-marker antibodies were used: The BD Stem Cell Enumeration Kit from BD Biosciences (catalog #344563) uses antibodies to CD45 (clone 2D1) and CD34 (clone 8G12). For the correlative extended CD34+ immunophenotyping by flow, the following fluorochrome-labeled antibodies were used (clone, source designated and catalog number in parenthesis): CD45-BUV395 (HI30, BD Biosciences, catalog #563792), CD123-BUV737 (7G3, Biosciences, catalog #741769), CD49f-BV421 (GoH3, BioLegend, catalog #313624), CD14-BV650 (M5E2, BioLegend, catalog #301836), CD45RA-BV785 (HI100, BioLegend, catalog #304140), CD34-VioBright515 (REA1164, Miltenyi, 1:120), CD10-PECF594 (HI10a, Biosciences, catalog #562396), CD38-PE-Cy7 (HIT2, BioLegend, catalog #303516), CD90-APC (5E10, BioLegend, catalog #328114), CD303-APCVIO770 (REA693, Miltenyi Biotech, catalog #130-120-517), CD184-PE (1D9, BD, catalog #551510). and CD184-PE (12G5, Biosciences, catalog #555974). Also see Supplemental Table 1: Antibodies for correlative CD34+ HSPC immunophenotyping.
Validation	The BD Stem Cell Enumeration Kit is a direct immunofluorescence-based three-color flow cytometric in vitro diagnostic assay (https://www.bdbiosciences.com/content/dam/bdb/products/global/reagents/flow-cytometry-reagents/clinical-diagnostics/multicolor-cocktails-and-kits-ivd-ce-ivds/344563_base/pdf/23-22014.pdf). All antibodies for correlative studies were purchased from commercial vendors (BD Biosciences, BioLegend, or Miltenyi Biotech) and used prior to their expiration date. All commercial antibodies underwent quality control testing before distribution as detailed online (https://www.bdbiosciences.com/en-us/products/reagents/flow-cytometry-reagents/research-reagents/quality-and-reproducibility, https://www.biolegend.com/en-us/quality/quality-control, https://www.miltenyibiotec.com/US-en/products/macs-antibodies/Antibody-production-development-and-quality-control.html#gref). Lot specific Certificates of Analyses are available online for each antibody listed in Supplemental Table 1 (https://regdocs.bd.com/regdocs/qcinfo, https://www.biolegend.com/en-us/certificate-of-analysis, https://www.miltenyibiotec.com/US-en/resources/technical-documents/certificates.html#gref).

Clinical data

Policy information about clinical studies

All manuscripts should comply with the ICMJE guidelines for publication of clinical research and a completed CONSORT checklist must be included with all submissions.

Clinical trial registration	NCT03246529
Study protocol	The full GENESIS Trial Protocol has been submitted as a supplemental document with this manuscript.
Data collection	Patients were enrolled from January 22, 2018 through October 30, 2020 at 18 sites in 5 countries (USA, Italy, Hungary, Spain and Germany). These sites include: Washington University School of Medicine in St. Louis, Siteman Cancer Center; University of Miami, Sylvester Comprehensive Cancer Center; Mayo Clinic, including Rochester, MN and Jacksonville, FL sites; University of Oregon Oregon Health & Science University; University of Kansas Medical Center; University of Maryland, Marlene and Stewart Greenebaum Comprehensive Cancer Center; University of Florida, Loyola University Chicago Health Sciences Campus, University of California Los Angeles, School of Medicine; University of Utah, Huntsman Cancer Institute; University of Rochester Medical Center, University of Cincinnati, Weill Cornell Medical College, UT M.D. Anderson Cancer Center, Azienda Ospedaliero-Universitaria "Policlinico-San Marco"; Grande Ospedale Metropolitano "Bianchi-Melacrino- Morelli"; University Hospital of Cologne; Ramon y Cajal University Hospital; 12Hospital University 12 De Octubre; Hospital de la Santa Creu i Sant Pau; University of Debrecen, Clinical Center; and Central Hospital of Southern Pest.
Outcomes	The primary objective of the study was to demonstrate the superiority of one dose of motixafortide+G-CSF over placebo+G-CSF to mobilize ≥6.0x10^6 CD34+ cells/kg in up to 2 apheresis sessions in preparation for autologous hematopoietic cell transplantation (auto-HCT) in multiple myeloma subjects. This was assessed by the primary endpoint of the proportion of subjects mobilizing

≥6.0x10^6 CD34+ cells/kg with up to 2 apheresis sessions in preparation for auto-HCT after G-CSF + single administration of BL-8040 or placebo + GCSF. The secondary objectives were to demonstrate the superiority of one dose of motixafortide+G-CSF over placebo +G-CSF to mobilize ≥2.0x10^6 CD34+ cells/kg in 1 apheresis session; to demonstrate the superiority of one dose of motixafortide+G-CSF over placebo+G-CSF to mobilize ≥6.0x10^6 CD34+ cells/kg in 1 apheresis session; to descriptively assess the comparability between the effects of motixafortide+G-CSF and placebo+G-CSF in time to neutrophil engraftment, platelet engraftment and the later of the two; and to descriptively assess the comparability between the effects of motixafortide+G-CSF and placebo+G-CSF on graft durability at 60 days, 100 days, as well as 6 and 12 months post-transplantation. These secondary endpoints were assessed by the proportion of subjects who collect ≥2.0 x 106 CD34+ cells/kg in 1 apheresis session; the proportion of subjects who collect ≥6.0 x 106 CD34+ cells/kg in 1 apheresis session; the time from transplantation to neutrophil engraftment defined as ANC ≥0.5x10^9/L for 3 consecutive days or ≥1.0 x 109/L for 1 day following the conditioning regimen associated nadir; the time from transplantation to platelet engraftment defined as the first of 3 consecutive measurements of platelet count ≥20x10^9/L without platelet transfusion support for 7 days following the conditioning regimen associated nadir; the time from transplantation to engraftment defined as the time to neutrophils and platelets engraftment, whichever comes later; and graft durability at 60 days, 100 days 6 months and 12 months post-transplantation, respectively.

Flow Cytometry

Plots

Confirm that:

☒ The axis labels state the marker and fluorochrome used (e.g. CD4-FITC).

☒ The axis scales are clearly visible. Include numbers along axes only for bottom left plot of group (a 'group' is an analysis of identical markers).

☒ All plots are contour plots with outliers or pseudocolor plots.

☒ A numerical value for number of cells or percentage (with statistics) is provided.

Methodology

Sample preparation

For the local lab-based CD34+ enumeration, sample preparation was performed according to standard operating procedures. For the central lab-based CD34+ enumeration, sample preparation was performed according to CLIA-based standard operating procedures, including a lyse-no wash single platform flow cytometry method allowing for the direct measurement of absolute cell counts using BD TruCount™ tubes. In the flow cytometry panel stem cells were identified based on CD34+ and CD45 expression. For the correlative flow cytometry studies, CD34+ HSPCs from N=51 patients (placebo+G-CSF N=13, plerixafor+G-CSF N=14, motixafortide+G-CSF N=24) were purified from apheresis product from apheresis day 1 via CD34+ immunomagnetic selection using an AutoMACS device (Miltenyi Biotech, Auburn, CA). CD34+ HSPCs were washed in phosphate-buffered saline (PBS) and stained 15 minutes at room temperature with a LIVE/DEAD Fixable Aqua Dead Cell Stain kit (Invitrogen, Carlsbad, CA). Cells were then washed in PBS supplemented with 0.5% bovine serum albumin and 2 mM EDTA and incubated for 10 min at room temperature with human Fc Block and Brilliant Stain Buffer (BD Biosciences; San Jose, CA). Samples were then incubated for 30 min at room temperature with pre-titrated saturating dilutions of the following fluorochrome-labeled antibodies (see Antibodies section for details). Fluorescence minus one controls were used to assess background fluorescence intensity and set gates for negative populations. After washing twice, samples were analyzed on a ZE5 (Bio-Rad, Hercules, CA) flow cytometer. Single stain compensation controls were obtained using UltraComp eBeads (Thermo Fisher Scientific) and data were analyzed using FCS Express (DeNovo Software, Pasadena, CA). The antibody-binding capacity (ABC) per cell of the different CD34+ HSPC subsets was determined for CD184 clones 12G5 and 1D9 using saturating concentrations of antibody and the Quantum Simply Cellular (QSC; Bangs Laboratories) system for fluorescence quantitation per the manufacturers recommendations.

Instrument

Local Lab studies: local lab dependent. Central Lab studies: BD FACS Canto II Flow Cytometer (Becton Dickinson, Franklin Lakes, NJ, USA). Correlative studies: ZE5 flow cytometer (Bio-Rad, Hercules, CA)

Software

Local Lab studies: local lab dependent. Central lab studies: Sample Acquisition (BD FACSDiVa Software version 8 program of the instrument) analysis was performed by counting 150,000 CD45+ events or 300 seconds. Correlative studies: FCS Express (DeNovo Software, Pasadena, CA).

Cell population abundance

Cells were CD34 selected as detailed above via immunomagnetic selection. During the subsequent flow experiments on CD34 selected cells, CD34+ was gated on to exclude any potential remaining CD34 negative cells. The gating strategy for the correlative studies is further detailed in Extended Data Figure 3 within the submitted manuscript and as below. Cell population abundance for each CD34+ fraction is detailed in both absolute numbers and percentage of total in Figure 3, Extended Data Figure 3 and Extended Data Figure 4.

Gating strategy

Local lab studies: gating strategy for local lab-based CD34+ enumeration was performed per local standard operating procedures. Central lab studies: gating strategy for central lab-based CD34+ enumeration was performed per validated method protocol. After acquisition, samples were gated appropriately in adherence with ISHAGE and CLIA protocols/ guidelines. Correlative studies: ISHAGE guidlines were followed. In addition, fluorescence minus one controls were used to assess background fluorescence intensity and set gates for negative populations. After washing twice, samples were analyzed on a ZE5 (Bio-Rad, Hercules, CA) flow cytometer. Single stain compensation controls were obtained using UltraComp eBeads (Thermo Fisher Scientific) and data were analyzed using FCS Express (DeNovo Software, Pasadena, CA). The gating strategy for the correlative studies is further detailed in Extended Data Figure 3 within the submitted manuscript.

☒ Tick this box to confirm that a figure exemplifying the gating strategy is provided in the Supplementary Information.
