# Supplementary material for: Motixafortide and G-CSF to mobilize hematopoietic stem cells for autologous transplantation in multiple myeloma: a randomized phase 3 trial
Source: Nat Med. 2023 Apr 17;29(4):869–79. doi: 10.1038/s41591-023-02273-z (PMC10115633; doi:10.1038/s41591-023-02273-z)
Supplement: Supplementary file 3 — Antibodies for correlative CD34+ HSPC immunophenotyping. BDB, BD Biosciences; BL, BioLegend; MB, Miltenyi Biotech; PBMCs, peripheral blood mononuclear cells; hPBMCs, human PBMCs; mPBMCs, murine PBMCs; hALL, human acute lymphoblastic leukemia; hAML, human acute myeloid leukemia. [file 41591_2023_2273_MOESM3_ESM.pdf]

Supplemental Information

Table 1. Antibodies for correlative CD34+ HSPC immunophenotyping.

Antigen	Fluorophore	Vendor	Catalog no.	Clone	Lot No.	Expiration date	Cells used for titering mAb	Volume per test (μL)
CD45	BUV395	BDB	563792	HI30	9269807	7/31/22	hPBMCs + mPBMCs	2.5
CD123	BUV737	BDB	741769	7G3	1119420	9/30/21	hPBMCs	1.25
CD49f	BV421	BL	313624	GoH3	B274314	9/30/21	hPBMCs	0.5
CD14	BV650	BL	301836	M5E2	B253716	12/31/20	hPBMCs	1.25
CD45RA	BV785	BL	304140	HI100	B246562	7/31/20	hPBMCs	1.25
CD34	VioBright515	MB	130-120-517	REA1164	1320060639	6/30/21	hAML	1.25
CD184	PE	BDB	555974	12G5	9280259	4/30/25	hPBMCs+hG2ALL	1
CD184	PE	BDB	551510	1D9	0051842	9/30/25	hPBMCs+hG2ALL	0.62
CD10	PECF594	BDB	562396	HI10a	0064623	12/31/20	hPBMCs + hALL	0.31
CD38	PC7	BL	303516	HIT2	B285552	3/31/23	hPBMCs	1.25
CD90	APC	BL	328114	5E10	B291361	6/30/24	hPBMCs + hALL	2.5
CD303	APC-Vio770	MB	130-113-652	REA693	1320120240	12/2/21	hPBMCs	1

Abbreviations: BDB, BD Biosciences; BL, BioLegend; MB, Miltenyi Biotech; PBMC, peripheral blood mononuclear cells; hPBMCs, human PBMCs, mPBMC, murine PBMCs, hALL, human acute lymphoblastic leukemia; hAML, human acute myeloid leukemia
